# Gotistobart or docetaxel in metastatic squamous non-small cell lung cancer: stage 1 of the randomized phase 3 PRESERVE-003 trial

**DOI:** 10.1038/s41591-026-04323-8

**Published:** 2026-03-27

**Authors:** Byoung Chul Cho, Rama Balaraman, Hua-Jun Chen, Xinmin Yu, Adewale Fawole, Zhi-gang Liu, Jiliang Zhang, Long Wu, Bin Yang, Jennifer L. Leddon, John Hamm, Yanjing Huang, Lin Wu, Pinhua Pan, Preet Singh, Andrew Beardsley, Fadi Kayali, Ardalan Davarifar, Ki Hyeong Lee, Keon-Uk Park, Youngjoo Lee, Luchun Li, Xicheng Wang, Meili Sun, Yan Yu, Vikram Jain, Svetlana Shpyro, Qiong Wang, Michael Wenger, Uğur Şahin, Sergey Efuni, Shiling Song, Kun He, Pan Zheng, Yang Liu, Kai He, Tianhong Li, Mark A. Socinski, Yi-Long Wu

**Affiliations:** 1 Division of Medical Oncology, Yonsei Cancer Center, Seoul, Republic of Korea; 2 Ocala Oncology Center PL, Ocala, FL USA; 3 Guangdong Lung Cancer Institute, Guangdong Provincial People’s Hospital, Guangdong Academy of Medical Sciences, Southern Medical University, Guangzhou, China; 4 Zhejiang Cancer Hospital, Hangzhou, China; 5 Florida Cancer Specialists and Research Institute, The Villages, FL USA; 6 Cancer Center, The Tenth Affiliated Hospital of Southern Medical University (Dongguan People’s Hospital), Guangzhou, China; 7 Chengdu Seventh People’s Hospital, Sichuan, China; 8 Renmin Hospital of Wuhan University, Wuhan, China; 9 Hubei Cancer Hospital, Wuhan, China; 10 University of Cincinnati, Cincinnati, OH USA; 11 Norton Cancer Institute, Louisville, KY USA; 12 Hainan General Hospital, Haikou, China; 13 Hunan Cancer Hospital, Changsha, China; 14 Xiangya Hospital, Central South University, Changsha, China; 15 Springfield Clinic Cancer Center, Springfield, IL USA; 16 Messino Cancer Center, Asheville, NC USA; 17 Florida Cancer Specialists, Sarasota, FL USA; 18 The University of Tennessee Medical Center, Knoxville, TN USA; 19 Chungbuk National University Hospital, Cheongju, Republic of Korea; 20 Keimyung University Dongsan Hospital, Daegu, Republic of Korea; 21 National Cancer Center, Goyang, Republic of Korea; 22 Chongqing Cancer Hospital, Chongqing, China; 23 The First Affiliated Hospital of Guangdong Pharmaceutical University, Guangzhou, China; 24 Jinan Central Hospital, Jinan, China; 25 Harbin Medical University–Tumor Hospital (The Third Affiliated Hospital), Harbin, China; 26 Mater Hospital, South Brisbane, Queensland Australia; 27 BioNTech SE, Mainz, Germany; 28 BioNTech US Inc., Cambridge, MA USA; 29 OncoC4 Inc., Rockville, MD USA; 30 R&G US, Inc., North Potomac, MD USA; 31 The Ohio State University James Cancer Hospital, Pelotonia Institute for Immuno-Oncology, Columbus, OH USA; 32 UC Davis Comprehensive Cancer Center, Sacramento, CA USA; 33 AdventHealth Cancer Institute, Orlando, FL USA

**Keywords:** Non-small-cell lung cancer, Randomized controlled trials

## Abstract

PRESERVE-003 is a two-stage phase 3 trial evaluating gotistobart (BNT316/ONC-392), a novel pH-sensitive anti-cytotoxic T lymphocyte-associated protein 4 (CTLA-4) antibody that selectively depletes regulatory T cells within the tumor microenvironment, in patients with metastatic squamous non-small cell lung cancer (sqNSCLC) without actionable genomic alterations who progressed on programmed cell death protein/programmed death ligand 1 inhibitor/platinum-based chemotherapy—a population with a poor prognosis. Here we report on stage 1, which aimed to confirm the dose and assess the preliminary efficacy (primary outcome: overall survival; secondary outcomes: progression‑free survival, objective response rate and duration of response) and safety of gotistobart compared to docetaxel. Patients with sqNSCLC were randomized (1:1) to gotistobart (6 mg kg^−1^ with two 10 mg kg^−1^ loading doses every 3 weeks (*N* = 45)) or docetaxel (75 mg m^−^^2^ every 3 weeks (*N* = 42)). After a median follow-up of 14.5 months, median overall survival was not reached with gotistobart (95% confidence interval (CI) 9.3 to not evaluable) versus 10.0 months (95% CI 6.2 to 11.9 months) with docetaxel (hazard ratio 0.46, 95% CI 0.25 to 0.84, nominal two-sided *P* = 0.0102). Safety was manageable, with grade ≥3 treatment-related adverse events in 42% and 49% of patients receiving gotistobart and docetaxel, respectively. Stage 1 results suggest that gotistobart monotherapy can provide clinically meaningful benefit for patients with programmed cell death protein/programmed death ligand 1-resistant and chemotherapy-resistant metastatic sqNSCLC. ClinicalTrials.gov identifier: NCT05671510.

## Abstract (web summary)

In the nonpivotal stage 1 of the randomized phase 3 PRESERVE-003 trial, patients with immunochemotherapy-resistant metastatic squamous non-small cell lung cancer without actionable genomic alterations treated with the next-generation, pH-sensitive anti-CTLA-4 agent gotistobart had encouraging overall survival outcomes compared to docetaxel.

## Main

Lung cancer remains the leading cause of cancer-related mortality worldwide, despite advances in targeted treatments and immunotherapy that have revolutionized the treatment of advanced non-small cell lung cancer (NSCLC), the most common lung cancer subtype^1–3^. NSCLC is further classified by histology, with squamous cell NSCLC (sqNSCLC) having a poorer prognosis than non-sqNSCLC^2^. Immune checkpoint inhibition with a programmed cell death protein/programmed death ligand 1 (PD-(L)1) inhibitor is standard of care in patients with metastatic sqNSCLC without actionable driver mutations, often in combination with or sequential to platinum-based chemotherapy (PBC)^4^. Once the disease progresses, treatment options are limited and have remained relatively unchanged over time^4,5^. Chemotherapy with docetaxel, with or without the vascular endothelial growth factor receptor 2 (VEGFR2) antibody ramucirumab, remains a standard of care. Docetaxel has modest efficacy, as patients with PD-(L)1-resistant sqNSCLC have a median survival ranging from 8.0 to 9.4 months, and treatment is associated with considerable toxicity^6–8^. These patients represent a lung cancer population with substantial unmet need.

Gotistobart (BNT316/ONC-392) is a novel tumor microenvironment-selective regulatory T cell (T_reg_) depletion antibody targeting cytotoxic T lymphocyte-associated protein 4 (CTLA-4)^9^. Unlike other clinically used anti-CTLA-4 antibodies, the acidic pH-sensitive mechanism of action of gotistobart avoids lysosomal degradation of both CTLA-4 and the anti-CTLA-4 antibody and thus preserves both the CTLA-4 molecules and the antibody^9^. By preserving high surface CTLA-4 density for more effective T_reg_ depletion in the tumor microenvironment, gotistobart exhibited superior antitumor activity and fewer immune-related adverse events (irAEs) in mice compared to the first-generation anti-CTLA-4 antibody ipilimumab^9^. In the first-in-human phase 1/2 PRESERVE-001 study in patients with advanced solid tumors in different tumor indications, gotistobart at doses of up to 10 mg kg^−1^ administered once every 3 weeks (Q3W) was clinically active with a manageable safety profile^10,11^. A preliminary analysis of 27 evaluable patients with metastatic NSCLC in PRESERVE-001 whose disease had progressed on prior PD-(L)1 inhibitors and PBC showed encouraging antitumor activity with gotistobart (10 mg kg^−1^ Q3W for four doses (*n* = 2; escalation cohort); 6 mg kg^−1^ with two loading doses of 10 mg kg^−1^ Q3W (*n* = 25; expansion cohort)). The response rate was 29.6% (one complete response and seven partial responses) and the disease control rate was 70.4% (refs. ^12,13^). A gotistobart dose of 6 mg kg^−1^ Q3W plus two loading doses of 10 mg kg^−1^ Q3W is further supported by pharmacokinetic analyses^13^. Data from the PRESERVE-001 study can be found in the protocol (included in the Supplementary Information) and full results from the study will be published after the prespecified follow-up period has been reached. The global phase 3 PRESERVE-003 study is an ongoing, two-stage, open-label, active-controlled trial evaluating the safety and efficacy of gotistobart versus docetaxel in patients with metastatic NSCLC without actionable genomic alterations, whose disease has progressed on one or more lines of prior therapy, including PD-(L)1 inhibitors and other immunotherapies after or in combination with PBC. The primary endpoint of PRESERVE-003 is overall survival (OS), and secondary endpoints include investigator-assessed progression-free survival (PFS), objective response rate (ORR) and safety. Here we report the efficacy and safety from the nonpivotal stage 1, with a focus on patients with metastatic sqNSCLC. Stage 1 is exploratory and was not powered to demonstrate efficacy.

## Results

### Patient disposition

**Fig. 1:**
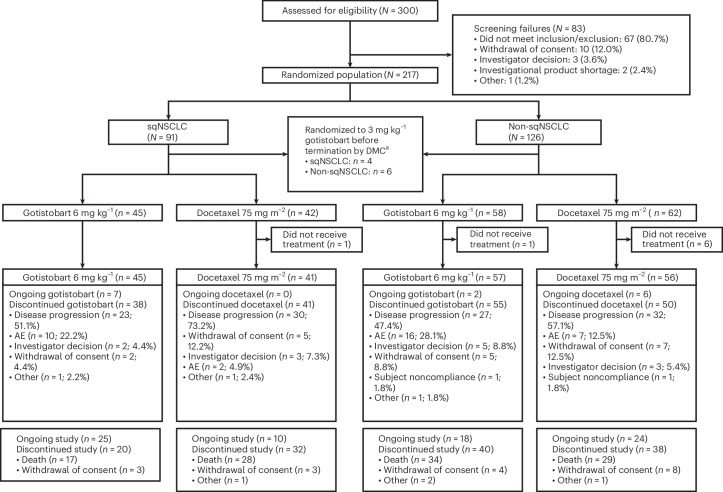
Patient disposition. ^a^Four patients with sqNSCLC and six patients with non-sqNSCLC were randomized to gotistobart 3 mg kg^−1^ before termination by the DMC.

Patients with metastatic NSCLC were randomized into stage 1 of the study from 27 June 2023 to 3 September 2024, in study centers in the USA, Australia, China, Korea and the UK. Three hundred patients were screened and 217 patients were enrolled in stage 1 of the study, of which 91 patients had sqNSCLC and 126 had non-sqNSCLC (Fig. 1). Four of the 91 patients with sqNSCLC were randomized to the gotistobart 3 mg kg^−1^ arm before its termination following the recommendation of the Data Monitoring Committee (DMC); the remaining 87 patients form the basis of the efficacy and safety analysis for patients with sqNSCLC presented here (data cutoff 8 August 2025). Forty-five patients were randomized to receive gotistobart at the selected dose of 6 mg kg^−1^, with two loading doses of 10 mg kg^−1^ Q3W, and 42 patients were randomized to receive docetaxel at 75 mg m^−^^2^ Q3W. The reasons for treatment discontinuation are listed in Fig. 1.

Patient demographics and baseline characteristics in patients with sqNSCLC were generally well balanced between treatment arms (Table 1). The median age across both arms was 65 years; approximately two-thirds of patients (69.0%) had received one prior line of systemic treatment for metastatic disease. All patients had prior treatment with a PD-(L)1 inhibitor and PBC per study inclusion criteria (80.5% of all patients had received concurrent PBC and anti-PD-(L)1). Two patients, both in the gotistobart treatment arm, had documented Kirsten rat sarcoma viral oncogene homolog (*KRAS*) mutations (although the exact mutation was not documented). Three patients in the gotistobart arm had prior treatment with an anti-CTLA-4, whereas no patients in the docetaxel arm had prior treatment with an anti-CTLA-4. More patients had liver or brain metastases at baseline in the gotistobart arm than in the docetaxel arm. The enrolled population was considered representative of a patient group whose disease has progressed on PD-(L)1 inhibitor and PBC.

**Table 1:** Baseline patient demographics and disease characteristics in patients with sqNSCLC

Characteristic		Gotistobart (*n* = 45)	Docetaxel (*n* = 42)
Median age (range) in years		64.0 (39–86)	68.5 (43–84)
Sex, no. (%)	Male	36 (80.0)	38 (90.5)
	Female	9 (20.0)	4 (9.5)
Race, no. (%)	Asian	32 (71.1)	30 (71.4)
	White	11 (24.4)	11 (26.2)
	Black or African American	2 (4.4)	0
	Other	0	1 (2.4)
Region, no. (%)	USA	11 (24.4)	11 (26.2)
	Non-USA^a^	34 (75.6)	31 (73.8)
ECOG PS score, no. (%)	0	9 (20.0)	7 (16.7)
	1	36 (80.0)	35 (83.3)
Smoking status, no. (%)	Never	3 (6.7)	4 (9.5)
	Current	8 (17.8)	11 (26.2)
	Former	34 (75.6)	27 (64.3)
Median (range) time from initial diagnosis to randomization in months		14.5 (5.9–65.6)	12.3 (4.0–72.5)
PD-(L)1 tumor proportion score, no. (%)	PD-(L)1 <1%	14 (31.1)	11 (26.2)
	PD-(L)1 1–49%	8 (17.8)	3 (7.1)
	PD-(L)1 ≥50%	7 (15.6)	12 (28.6)
	Unknown	16 (35.6)	16 (38.1)
Metastases at baseline, no. (%)	Yes	45 (100.0)	42 (100.0)
	Liver	6 (13.3)	2 (4.8)
	Brain	6 (13.3)	4 (9.5)
Number of prior lines of therapy in the advanced/metastatic setting, no. (%)	1	30 (66.7)	30 (71.4)
	2	14 (31.1)	8 (19.0)
	≥3	1 (2.2)	4 (9.5)
Prior anticancer therapy, no. (%)	Radiotherapy	27 (60.0)	18 (42.9)
	Surgery	14 (31.1)	9 (21.4)
	Platinum chemotherapy	45 (100.0)	42 (100.0)
	Anti-PD-(L)1	45 (100.0)	42 (100.0)
	CTLA-4 therapy	3 (6.7)	0

^a^Non-USA included patients recruited from Australia, China, Korea and the UK.

Baseline patient demographics and disease characteristics in patients with non-sqNSCLC are reported in Supplementary Table 1.

### Primary outcomes in patients with sqNSCLC

**Fig. 2:**
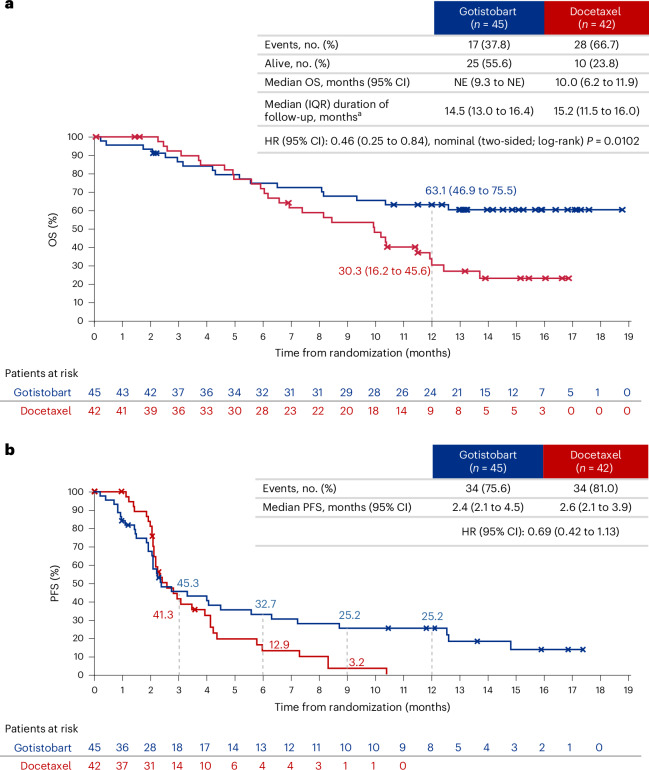
OS and PFS. **a**,**b**, Kaplan–Meier estimates of OS (**a**) and PFS (investigator assessment) (**b**) in patients with sqNSCLC. The numbers and percentages of patients with an OS event and those alive as of the data cutoff are listed in the tables. The dashed line indicates the 12-month OS rate, and the crosses indicate censored patients. Seven patients withdrew from the study before death and were censored at their last known alive date: three patients in the gotistobart arm and four patients in the docetaxel arm. The *P* value was nominal, that is, not from formal hypothesis testing. ^a^The duration of follow-up in months was calculated using a reversed Kaplan–Meier analysis, with OS events censored and deaths treated as events. IQR, interquartile range.

The primary endpoint for the PRESERVE-003 study is OS. As shown in Fig. 2a, gotistobart demonstrated a clinically meaningful survival benefit (primary endpoint), with a 54% reduction in the risk of mortality compared to docetaxel in patients with sqNSCLC. At a median follow-up of 14.5 months (range 0.1–18.8 months) and 45/87 (51.7%) events, median OS was not reached (95% confidence interval (CI) 9.3 months to not evaluable (NE)) with gotistobart and 10.0 months (95% CI 6.2 to 11.9 months) with docetaxel (hazard ratio (HR) 0.46, 95% CI 0.25 to 0.84, two-sided *P* value = 0.0102). The OS curves separated after 6 months, with improvement throughout the observation period. At 12 months, the OS rate doubled with gotistobart (63.1%; 95% CI 46.9% to 75.5%) compared to docetaxel (30.3%; 95% CI 16.2% to 45.6%). Forty percent of all patients (18/45) in the gotistobart arm and 61.9% of patients (26/42) in the docetaxel arm received subsequent anticancer systemic therapy (Supplementary Table 2), and the median time to initiation of subsequent cancer treatment was approximately 1 month after the last treatment dose. Neither patient in the gotistobart arm with a documented *KRAS* mutation received prior or subsequent KRAS-targeted therapy.

### Secondary outcomes in patients with sqNSCLC

**Fig. 3:**
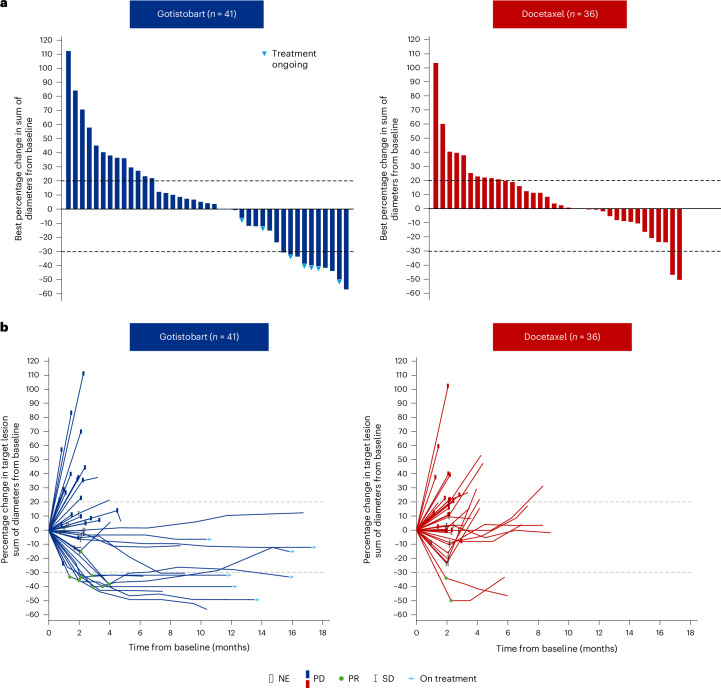
Antitumor efficacy. **a**,**b**, Tumor response: the best percentage change from baseline by treatment (investigator assessment) (**a**) and percentage change from baseline over time by treatment (investigator assessment) (**b**) in patients with sqNSCLC. PD, progressive disease; PR, partial response; SD, stable disease.

Secondary efficacy endpoints of the study include PFS and ORR per investigator assessment based on Response Evaluation Criteria in Solid Tumours (RECIST) 1.1. Despite similar median PFS of 2.4 months (95% CI 2.1 to 4.5 months) with gotistobart and 2.6 months (95% CI 2.1 to 3.9 months) with docetaxel, a trend with improved PFS was observed in the gotistobart arm over docetaxel (HR 0.69, 95% CI 0.42 to 1.13) (Fig. 2b). The confirmed ORR was 20.0% (95% CI 9.6% to 34.6%) with gotistobart compared to 4.8% (95% CI 0.6% to 16.2%) with docetaxel (Table 2). Tumor response in terms of best percentage change from baseline by treatment is shown in Fig. 3a. Responses to gotistobart were durable, with a median duration of response (DoR) of 11.0 months (95% CI 3.5 months to NE) compared to 3.8 months (95% CI 3.6 months to NE) with docetaxel (Table 2 and Fig. 3b). Seven patients (15.6%) had received gotistobart for 12 months or longer.

**Table 2:** Efficacy summary, PFS, ORR and DoR for patients with sqNSCLC: tumor assessments were evaluated by investigators according to RECIST 1.1

Endpoint	Gotistobart (*n* = 45)	Docetaxel (*n* = 42)
Median OS, months (95% CI)	NE (9.3 to NE)	10.0 (6.2 to 11.9)
HR (95% CI)	0.46 (0.25 to 0.84)	
Nominal *P* value (two-sided log rank)	0.0102	
12-month OS rate, % (95% CI)	63.1 (46.9 to 75.5)	30.3 (16.2 to 45.6)
Median PFS, months (95% CI)	2.4 (2.1 to 4.5)	2.6 (2.1 to 3.9)
HR (95% CI)	0.69 (0.42 to 1.13)	
12-month PFS rate, % (95% CI)	25.2 (13.2 to 39.2)	0 (NE to NE)
Confirmed ORR, no. (%; 95% CI)	9 (20.0; 9.6 to 34.6)	2 (4.8; 0.6 to 16.2)
Best overall response, no. (%)		
Complete response	0	0
Partial response	9 (20.0)	2 (4.8)
Stable disease	7 (15.6)	14 (33.3)
Progressive disease	22 (48.9)	19 (45.2)
NE	4 (8.9)	1 (2.4)
Missing	3 (6.7)	6 (14.3)
Median DoR, months (95% CI)	11.0 (3.5 to NE)	3.8 (3.5 to NE)

### Efficacy outcomes in patients with non-sqNSCLC and in patients treated with gotistobart 3 mg kg^−1^ Q3W

For patients with non‑sqNSCLC, the median OS, 12-month survival rates and median PFS were numerically better in the docetaxel arm than in the gotistobart arm (6 mg kg^−1^ Q3W with two loading doses of 10 mg kg^−1^) (Supplementary Table 3). These data supported the DMC’s recommendation to pause clinical development for patients with non-sqNSCLC. Poor clinical outcomes in patients with sqNSCLC or non‑sqNSCLC who received gotistobart 3 mg kg^−1^ Q3W (Supplementary Table 3) led the DMC to recommend terminating the low-dose cohort after the first ten patients were randomized to the 3 mg kg^−1^ arm.

### Safety

In patients with sqNSCLC, at least one any-grade adverse event (AE) was experienced by all patients treated with gotistobart and 40/41 patients (97.6%) treated with docetaxel, and the rate of grade ≥3 AEs was similar (66.7% with gotistobart and 63.4% with docetaxel). Ten patients treated with gotistobart (22.2%) and two patients treated with docetaxel (4.9%) discontinued treatment owing to AEs (Supplementary Table 4); these AEs were judged to be treatment related in six patients in the gotistobart arm and in both patients in the docetaxel arm.

The rate of treatment-related AEs (TRAEs) with gotistobart was similar to that with docetaxel (84.4% versus 90.2%, respectively), as was the rate of grade ≥3 TRAEs (42.2% versus 48.8%, respectively) (Table 3). Diarrhea and increased alanine aminotransferase (ALT) were the most frequent TRAEs with gotistobart (both in 28.9% of patients), whereas anemia (36.6%) and decreased neutrophil count (24.4%) were the most frequent TRAEs with docetaxel. The most frequent grade ≥3 TRAEs were colitis (8.9%) and increased ALT (6.7%) with gotistobart and decreased neutrophil count (24.4%) and decreased white blood cell count (14.6%) with docetaxel. The most frequently reported TRAEs with gotistobart were in line with the expected safety profile of immune checkpoint inhibitors. No fatal TRAEs were reported; the two patient deaths due to AEs in the gotistobart arm (hemoptysis and pneumonia) were not considered to be treatment related by the investigators.

**Table 3:** Summary of TRAEs assessed by investigators in patients with sqNSCLC

AEs, no. (%)	Gotistobart (*n* = 45)		Docetaxel (*n* = 41)	
	Any grade	Grade ≥3	Any grade	Grade ≥3
Any	38 (84.4)	19 (42.2)	37 (90.2)	20 (48.8)
Any serious AE	19 (42.2)	–	12 (29.3)	–
AEs in at least 10% of the patients in either treatment group				
Increased ALT	13 (28.9)	3 (6.7)	4 (9.8)	0
Diarrhea	13 (28.9)	2 (4.4)	4 (9.8)	0
Increased AST	12 (26.7)	2 (4.4)	2 (4.9)	0
Chills	10 (22.2)	1 (2.2)	0	0
Decreased platelet count	8 (17.8)	1 (2.2)	4 (9.8)	0
Infusion-related reaction	8 (17.8)	0	0	0
Anemia	7 (15.6)	0	15 (36.6)	1 (2.4)
Decreased appetite	7 (15.6)	0	6 (14.6)	1 (2.4)
Decreased weight	7 (15.6)	0	2 (4.9)	0
Nausea	6 (13.3)	0	6 (14.6)	0
Vomiting	6 (13.3)	0	2 (4.9)	0
Pyrexia	6 (13.3)	0	0	0
Rash	6 (13.3)	1 (2.2)	1 (2.4)	0
Decreased neutrophil count	5 (11.1)	0	10 (24.4)	10 (24.4)
Decreased blood lactate dehydrogenase	5 (11.1)	0	1 (2.4)	0
Colitis	5 (11.1)	4 (8.9)	0	0
Pruritus	5 (11.1)	0	0	0
Decreased white blood cell count	4 (8.9)	0	9 (22.0)	6 (14.6)
Fatigue	3 (6.7)	1 (2.2)	6 (14.6)	0
Pneumonia	3 (6.7)	2 (4.4)	5 (12.2)	1 (2.4)
Alopecia	0	NA	7 (17.1)	NA

AST, aspartate aminotransferase; NA, not applicable.

Overall, 60.0% of patients (*n* = 27) in the gotistobart arm experienced at least one irAE of any grade compared to 9.8% of patients (*n* = 4) in the docetaxel arm (Supplementary Table 5). Seventeen of the 27 patients (63.0%) who experienced an irAE in the gotistobart arm required steroids for the management of irAEs. Grade ≥3 irAEs were experienced by 33.3% of patients (*n* = 15) in the gotistobart arm and 4.9% of patients (*n* = 2) in the docetaxel arm. The most frequent irAE with gotistobart was diarrhea, reported in 22.2% of patients, with 4.4% experiencing grade ≥3 severity.

The median time to onset of diarrhea or colitis was 24.5 days (range 1–201 days) after the first dose of gotistobart and 5.5 days (range 1–37 days) after the first dose of docetaxel. Immune-mediated hepatic adverse reactions had a median time to onset of 62 days (range 20–316 days) after the first dose of gotistobart (no events recorded in the docetaxel arm). Immune-mediated pneumonitis had a median time to onset of 83 days (range 43–191 days) after the first dose of gotistobart (no events recorded in the docetaxel arm).

The rate of serious AEs considered by the investigators to be treatment related was 42.2% in patients treated with gotistobart and 29.3% in patients treated with docetaxel. Serious TRAEs occurring in more than one patient included colitis (11.1%), immune-mediated lung disease (6.7%) and pneumonia (4.4%) in the gotistobart arm and pneumonia (9.8%) and febrile neutropenia (7.3%) in the docetaxel arm.

The safety profile of gotistobart in patients with non-sqNSCLC (Supplementary Table 6) and in patients with mixed histology who received gotistobart 3 mg kg^−1^ Q3W (Supplementary Table 7) was consistent with that reported in patients with sqNSCLC.

## Discussion

The stage 1 results of PRESERVE‑003 demonstrate a clinically meaningful advantage of gotistobart over docetaxel in patients with PD‑(L)1- and chemotherapy‑resistant metastatic sqNSCLC. In patients with sqNSCLC, OS, as the primary endpoint, showed the most pronounced benefit, with gotistobart achieving a 54% reduction in the risk of death and a median OS not yet reached at the time of analysis, compared to 10.0 months for docetaxel. The doubling of the 12‑month OS rate highlights the durability of benefit in a population with historically poor outcomes.

The delayed separation of the OS curves observed in this study is consistent with the known kinetics of immune checkpoint inhibition, which requires time for immune activation before antitumor effects are observed radiographically, in contrast to the immediate effects of cytotoxic chemotherapy^14^.

Although median PFS was similar between treatment arms, the HR (0.69) indicated a trend toward improved disease control with gotistobart. The PFS tail favored gotistobart, with a 12‑month PFS rate of 25.2% (95% CI 13.2% to 39.2%) compared to 0% for docetaxel. This late separation of the PFS curves parallels the OS pattern and suggests that a subset of patients derives sustained immunological benefit, a hallmark of effective checkpoint modulation. Immunotherapy, such as gotistobart, may not immediately prevent disease progression but can lead to long-term survival benefits, for example, through durable immune responses. The ORR was 20% (95% CI 9.6% to 34.6%) with gotistobart and 4% (95% CI 0.6% to 16.2%) with docetaxel, and responses were notably durable, with a median (95% CI) DoR of 11.0 months (3.5 months to NE) with gotistobart versus 3.8 months (3.5 months to NE) with docetaxel. In the TROPION-Lung01 trial, the docetaxel ORR was 12.7% (95% CI 6.0% to 22.7%) in PD-1-resistant patients with sqNSCLC (*N* = 71 sqNSCLC treated with docetaxel)^7^.

Improving on the modest efficacy seen with docetaxel, the standard of care for over 20 years, has been an elusive goal in previously treated sqNSCLC. Multiple studies assessing novel therapeutic approaches in the post-platinum, post-PD-(L)1 inhibitor setting have failed to demonstrate an OS benefit over docetaxel, including tyrosine kinase inhibitors plus PD-(L)1 inhibitor in the CONTACT-01 (atezolizumab plus cabozantinib)^6^, LEAP-008 (lenvatinib with or without pembrolizumab)^15^ and SAFFRON-301 (tislelizumab plus sitravatinib)^16^ studies, and trophoblast cell surface antigen 2-directed antibody–drug conjugates in the TROPION-Lung01 (datopotamab deruxtecan)^7^ and EVOKE-01 (sacituzumab govitecan)^8^ studies. These studies had docetaxel as an active control arm. The PRAGMATICA-LUNG study evaluating pembrolizumab plus ramucirumab also failed to demonstrate an OS benefit over standard of care treatment (investigator’s choice, including docetaxel or combinations thereof)^17^. The above studies had about 20–30% patients with sqNSCLC, and there was no significant improvement in OS. These previous failures highlight the significance of our findings in this setting. Furthermore, the median OS reported with docetaxel in our study is relatively higher than the upper end of the range reported by other studies in a second-line sqNSCLC population (8.0 to 9.4 months)^6–8^. While other anti-CTLA-4 antibodies have not been extensively studied in this treatment setting, tremelimumab did not induce an antitumor response in PD-(L)1-resistant sqNSCLC, even when used in combination with the anti-PD-L1 antibody durvalumab^18^.

PRESERVE-003 originally enrolled patients with non-sqNSCLC and sqNSCLC, but a futility analysis by NSCLC histology subgroups led to an independent recommendation from the DMC to limit enrollment to patients with sqNSCLC. Tumors of squamous histology are often characterized by higher levels of tumor mutational burden and increased neoantigen expression compared to nonsquamous tumors^10,11^. They also tend to have a more inflamed immune microenvironment, with higher levels of immune cell infiltration, including T cells^19^. Furthermore, while sqNSCLC is known to have a complex genomic profile and a very low prevalence of actionable genomic alterations^20^, recent studies demonstrated that >55% of sqNSCLC samples have either homozygous or heterozygous deletion of the phosphatase and tensin homolog (*PTEN*) tumor suppressor gene. Downregulation of *PTEN* was observed in 62% of sqNSCLC samples^21^. Preclinical and clinical studies have shown that *PTEN* defects are associated with increased T_reg_ infiltration^21^, which in turn correlates with poor prognosis^22^. These higher levels of immune cell infiltration mean that T_reg_-depleting/inactivating antibodies, including anti-CTLA-4 antibodies, may be more likely to provide clinical benefit in patients with sqNSCLC. Gotistobart has been shown to be more effective than other approved anti-CTLA-4 antibodies in depleting T_regs_ in the tumor microenvironment in preclinical studies^9,23^, perhaps underpinning the clinical activity observed in patients with sqNSCLC. Physical depletion of T_regs_ is expected to yield superior antitumor efficacy compared to CTLA-4 blockade alone, as T_regs_ suppress effector T cell responses through multiple redundant mechanisms beyond the CTLA-4 axis^24^. These mechanisms include acting as a high-affinity ‘sink’ for interleukin (IL)-2 in the tumor microenvironment, generating immunosuppressive adenosine via the cluster of differentiation (CD)39/CD73 pathway, and secreting inhibitory cytokines such as transforming growth factor beta and IL-10. By selectively depleting the T_reg_ population, the cellular driver of multiple suppression pathways within the tumor microenvironment, gotistobart offers a more profound restoration of antitumor immunity than CTLA-4 receptor blockade alone. Consistent with these biological distinctions, the clinical results from stage 1 of PRESERVE-003 demonstrated a differential treatment effect between the two histologies as only patients with sqNSCLC derived meaningful clinical benefit from gotistobart when compared to those who received docetaxel.

Preclinical studies demonstrate that gotistobart exhibits greater antitumor activity and a lower risk of irAE than other anti-CTLA-4 antibodies tested owing to its unique mechanism of action^9^. Gotistobart binds well to CTLA-4 at pH 6.0 or above but disassociates from its target at pH 6.0 or lower^9^. This pH dependency allows it to avoid antibody-induced lysosomal degradation of CTLA-4, the root cause of toxicity and reduced antitumor activities of other anti-CTLA-4 antibodies in preclinical models^9^. Further preclinical studies demonstrated that engineering acidic pH sensitivity into ipilimumab conferred improved antitumor activity in a preclinical tumor model while reducing the risk of irAEs^25^. The clinical data presented herein provide clinical proof of concept for preserving the CTLA-4 immune checkpoint to achieve safer and more effective therapeutic targeting of CTLA-4 (ref. ^26^).

The AE profile of gotistobart was consistent with previous data from the PRESERVE-001 study^10^ and is aligned with the well-characterized immune-related toxicities reported for ipilimumab, including gastrointestinal, hepatic, endocrine and pulmonary immune-mediated events, which dominate the toxicity spectrum of CTLA-4-directed therapies^27–29^. The time to onset of irAEs was also consistent with the literature^30^. No new safety signals were identified. Mitigation strategies include close monitoring (particularly for events such as colitis and immune-mediated lung disease), dose interruptions and management with corticosteroids and/or supportive care, according to American Society of Clinical Oncology and National Comprehensive Cancer Network guidelines^5,30^.

Overall, the data from stage 1 of the PRESERVE-003 trial showed that gotistobart at 6 mg kg^−1^ following two loading doses of 10 mg kg^−1^ Q3W conferred therapeutic efficacy in patients with sqNSCLC while maintaining a manageable safety profile. For context, ipilimumab—another CTLA‑4-directed antibody—is approved at substantially lower doses (typically 3 mg kg^−1^) (refs. ^31–34^), which limits the appropriateness of direct safety or efficacy comparisons between the two agents. Moreover, ipilimumab has not been evaluated in this specific post-PD‑(L)1, chemotherapy‑resistant sqNSCLC population, further constraining cross‑trial interpretation. The results presented here indicate that the selected dose of gotistobart yields a clear clinical benefit in this setting.

The use of OS as the primary endpoint, considered the gold standard in randomized controlled trials, is a strength of this study. Also, the inclusion of histology as a stratification factor for randomization enabled balanced treatment allocation in the sqNSCLC population. A limitation of the study is that the Asian patient population was overrepresented, and Hispanic and Black/African American patients were underrepresented in stage 1. The use of investigator assessment rather than blinded, independent central reviewer assessment of PFS and ORR may be limited by the open-label design. It should also be considered that the stage 1 part of this trial reported here is exploratory; the ongoing pivotal stage 2 of PRESERVE-003 is designed to validate the role of gotistobart in this population of patients with a high unmet need for new treatment options.

This randomized study demonstrates a clinically meaningful improvement in OS with a novel immunotherapy monotherapy compared to docetaxel in patients with sqNSCLC who experience disease progression on or after anti-PD-(L)1 treatment, given either in combination with or after PBC. In conclusion, with a 54% reduction in the risk of death over docetaxel and manageable toxicity, the results from the nonpivotal stage 1 of this phase 3 randomized study suggest that gotistobart may offer a chemotherapy-free treatment option to transform the treatment paradigm of sqNSCLC with better outcomes for patients.

## Methods

### Ethics approval and consent

The study was conducted in accordance with local and national regulations, as well as consensus ethical principles derived from the Declaration of Helsinki, the Council for International Organizations of Medical Sciences International Ethical Guidelines and the International Council for Harmonisation Good Clinical Practice Guidelines. All study procedures, protocols and other relevant documents were approved by the institutional review board/ethics committee and national regulatory authority. The central Western Institutional Review Board provided approval in the USA. In Australia, China, Korea and the UK, the independent review boards or ethics committees at each study site provided approval (Supplementary Information). The independent DMC provided oversight of the study, reviewing the data and providing recommendations. The study investigators recruited patients and obtained consent. All patients provided written informed consent before enrollment. Patients were not compensated for participation.

### Study design and patients

PRESERVE-003 (ClinicalTrials.gov registration: NCT05671510; date of registration: 4 January 2023) is a two-stage (nonpivotal stage 1A and 1B, pivotal stage 2), randomized, open-label, active-controlled phase 3 trial evaluating the safety and efficacy of gotistobart versus docetaxel in patients with metastatic NSCLC after progression on a PD-(L)1 inhibitor. Nonpivotal stage 1A was designed to confirm the gotistobart dose in patients with metastatic non-sqNSCLC and sqNSCLC. A total of 120 patients were randomized (1:1:1) to gotistobart 3 mg kg^−1^ Q3W, gotistobart 6 mg kg^−1^ with two loading doses of 10 mg kg^−1^ Q3W or docetaxel 75 mg m^−^^2^ Q3W. However, the 3 mg kg^−1^ gotistobart dose arm was discontinued after ten patients (six patients with non-sqNSCLC and four patients with sqNSCLC) had been treated following an independent DMC recommendation (version 2.0 of the protocol was issued on 27 June 2024). Nonpivotal stage 1B continued with 1:1 randomization to gotistobart 6 mg kg^−1^ with two loading doses of 10 mg kg^−1^ Q3W or docetaxel 75 mg m^−^^2^ Q3W. A subsequent futility analysis by NSCLC histology subgroups led to an independent recommendation from the DMC to focus the PRESERVE-003 population on patients with sqNSCLC (version 3.0 of the protocol was issued on 26 September 2024 and is included in the Supplementary Information). Patients with nonsquamous histology who were randomized to the gotistobart arm were encouraged to withdraw from the study.

The pivotal stage 2 is ongoing to evaluate the safety and efficacy of gotistobart versus docetaxel (1:1 randomization to gotistobart 6 mg kg^−1^ with two loading doses of 10 mg kg^−1^ Q3W or docetaxel 75 mg m^−^^2^ Q3W) in patients with sqNSCLC.

Except for histology, stage 1 and stage 2 of the study have the same eligibility criteria. Eligible patients were ≥18 years of age with a histologically or cytologically confirmed diagnosis of metastatic non-sqNSCLC (stage 1) or sqNSCLC (stage 1 and stage 2). NSCLC with targetable mutations or genomic alterations in epidermal growth factor receptor (EGFR), proto-oncogene tyrosine-protein kinase-1 (ROS1), mesenchymal-to-epithelial transition protein (MET), B-raf proto-oncogene (BRAF), rearranged during transfection (RET), neurotrophic tyrosine receptor kinase (NTRK), anaplastic lymphoma kinase protein (ALK) or human epidermal growth factor receptor 2 (HER2) was not permitted. Patients must have had radiographic disease progression after the most recent treatment with either a standard-dose PD-(L)1 inhibitor (for ≥12 weeks) in combination with PBC, or ≥2 cycles of PBC followed by a standard-dose PD-(L)1 inhibitor (for ≥12 weeks). Prior treatment with PD-(L)1 inhibitor combinations with anti-CTLA-4, anti-lymphocyte activation gene-3 (LAG-3), T cell immunoreceptor with immunoglobulin and immunoreceptor tyrosine-based inhibitory motif domains (TIGIT), VEGF or VEGFR antibodies was permitted. Patients must have had measurable disease per RECIST 1.1 and Eastern Cooperative Oncology Group performance status (ECOG PS) 0 or 1, with adequate organ function and a life expectancy of at least 3 months. The presence of active or symptomatic brain metastases or evidence of progression within 4 weeks before study drug dosing excluded patients from the study. Current interstitial lung disease or noninfectious pneumonitis, or clinically relevant pleural effusion within 28 days before the study drug dosing, excluded patients from the study, as did a history of interstitial lung disease or noninfectious pneumonitis associated with prior therapy that required management with a high-dose steroid. Other exclusion criteria included AEs due to cancer treatment that had not recovered to National Cancer Institute Common Terminology for Adverse Events v5.0 grade ≤1 except endocrinopathy or peripheral neuropathy in which recovery to grade ≤2 was allowed, last anti-PD-1/PD-L1 dose within 28 days of first dose of study treatment and receipt of systemic steroid therapy with >10 mg day^−1^ prednisone or equivalent within 7 days of the first dose of study treatment. Sex was patient reported and based on medical records. No analyses based on sex were conducted owing to the exploratory nature of stage 1.

### Randomization and stratification factors

Eligible patients were randomized using the Interactive Web Response System to receive gotistobart or docetaxel treatment stratified by factors that could impact prognosis: histology (squamous versus nonsquamous (stage 1 only)), presence of brain metastases (yes or no), ECOG PS score (0 versus 1) and region (USA and ex-USA).

### Treatments

On day 1 of each 3-week cycle, patients were administered either gotistobart or docetaxel by intravenous infusion. In stage 1A, gotistobart was administered at 3 mg kg^−1^ or 6 mg kg^−1^ (with two loading doses of 10 mg kg^−1^), and in stage 1B, gotistobart was administered at 6 mg kg^−1^ (with two loading doses of 10 mg kg^−1^). Docetaxel was dosed at 75 mg m^−^^2^.

Patients could receive up to 17 cycles (approximately 1 year) or until disease progression, unacceptable toxicity, withdrawal of consent or other reasons. Continuation of treatment beyond disease progression was permitted if the investigator considered there to be potential clinical benefit for the patient. Where disease control was achieved and maintained through the end of the 17 cycles, the option to continue treatment beyond 1 year was available. Patients who discontinued docetaxel were not permitted to cross over to the gotistobart arm.

Dose adjustments in the event of TRAEs were permitted, with up to one dose adjustment allowed in the gotistobart arm (10 mg kg^−1^ could be reduced to 6 mg kg^−1^ and 6 mg kg^−1^ could be reduced to 3 mg kg^−1^) with the option to return to the original dose at the investigator’s discretion. Up to three dose reductions were permitted in the docetaxel arm. Dose delay was also permitted.

Gotistobart dosing was delayed for any AE considered immune related and was restarted at the investigator’s discretion once toxicity had resolved, with dose modifications per protocol-specified management recommendations for irAEs. Gotistobart-related diarrhea or colitis AEs were managed in accordance with the protocol-recommended management plan, based on published guideline recommendations, including close monitoring, dose interruptions and administration of corticosteroids and/or supportive care (Supplementary Table 8). No prophylaxis for diarrhea was specified in the study protocol. Gotistobart-related liver function abnormalities and hepatitis were also managed in accordance with the protocol-recommended management plan (close monitoring, dose interruptions and the administration of corticosteroids and/or supportive care; Supplementary Table 8). Grade 2 pneumonitis AEs were managed with corticosteroids and close monitoring; gotistobart was permanently discontinued for AEs of grade ≥3. For the management of other irAEs, the protocol specified that investigators should follow published guidelines^5,30^.

### Endpoints

The study’s primary endpoint was OS, and secondary endpoints included investigator-assessed PFS and ORR per RECIST 1.1, and safety in terms of the incidence of AEs (treatment emergent), TRAEs, irAEs and rates of treatment discontinuation due to AEs.

### Assessments

OS was monitored for the duration of the study. Tumor response and PFS per RECIST 1.1 were determined by conducting computed tomography or magnetic resonance imaging at baseline (within 28 days before day 1 of cycle 1) and every 9 ± 1 weeks for the first year and every 16 ± 3 weeks after the first year until confirmed progressive disease. Upon observation of response or progression, a confirmatory scan was conducted within 4–6 weeks of the initial evaluation. Patients discontinuing treatment for any reason other than disease progression were scanned every 9 ± 1 weeks until disease progression or initiation of another anticancer treatment to determine PFS.

AEs were reported and graded according to National Cancer Institute Common Terminology for Adverse Events v5.0 by the study investigator, who also assessed potential relatedness to the study treatment and whether they were considered immune related. Serious AEs and irAEs were collected from the start of study treatment through 90 days after the last dose of the treatment or the date the patient initiated new anticancer therapy, whichever occurred first. Other safety evaluations (vital signs, physical examination, ECOG PS, medical history and laboratory tests) were performed at baseline and at regular intervals throughout the study or whenever medically indicated.

### Statistical analysis

Efficacy analyses were conducted in all patients with sqNSCLC randomized to either gotistobart 6 mg kg^−1^ with two loading doses of 10 mg kg^−1^ Q3W or docetaxel 75 mg m^−^^2^ Q3W in stage 1 and was based on observed data only. The effective sample size at each assessment of different analyses was grounded in the total number of patients with nonmissing data in the parameter of interest at that specific visit.

OS was defined as the time from randomization to death due to any cause. Patients without reported death were censored at the last date known to be alive. OS curves were generated using Kaplan–Meier estimates. HRs and associated 95% CIs were calculated from a Cox proportional-hazards model. The duration of follow-up in months was calculated using a reversed Kaplan–Meier analysis, with OS events censored and deaths treated as events^35^. ORR was determined per RECIST 1.1 and the corresponding 95% CIs were calculated using the exact binomial distribution. PFS was defined as the time from the date of randomization until the date of the first documentation of progressive disease or death by any cause (whichever occurred first). For patients who had no disease progression and were alive at the time of analysis, the date of the last tumor assessment was taken as the censored date. For patients who started new anticancer therapy before disease progression or death, the date of the last tumor assessment on or before initiation of new anticancer therapy was taken as the censored date. For patients with disease progression or who died after more than one missed visit, the date of the last tumor assessment before the missed visit was taken as the censored date. PFS was analyzed using the same method as OS.

Safety analyses were conducted in all patients with sqNSCLC who received at least one dose of study medication and were summarized using descriptive statistics. Data collection and query handling was managed using an electronic data capture system (CONFORM, EDETEK). Statistical analyses were conducted using SAS (version 9.4 or later).

### Reporting summary

Further information on research design is available in the Nature Portfolio Reporting Summary linked to this article.

## Online content

Any methods, additional references, Nature Portfolio reporting summaries, source data, extended data, supplementary information, acknowledgements, peer review information; details of author contributions and competing interests; and statements of data and code availability are available at 10.1038/s41591-026-04323-8.

## Supplementary information

Supplementary Information List of institutional review board centers, Supplementary Tables 1–8 and protocol document.

Reporting Summary

## Data Availability

This trial is currently ongoing. Upon completion of this clinical trial, the data that support the findings of this study will be made available to qualified researchers. Proposals should be directed to pzheng@oncoc4.com. To gain access, data requestors will need to sign a data access agreement. All data provided will be anonymized to respect the privacy of patients who have participated in the trial, in line with applicable laws and regulations. Data will be made available; to qualified researchers upon request, within 12 months of the study completion data and for a period of 2 years thereafter.
