# Supplementary material for: Gotistobart or docetaxel in metastatic squamous non-small cell lung cancer: stage 1 of the randomized phase 3 PRESERVE-003 trial
Source: Nat Med. 2026 Mar 27;32(6):2245–53. doi: 10.1038/s41591-026-04323-8 (PMC13278949; doi:10.1038/s41591-026-04323-8)
Supplement: Supplementary file 1 — List of institutional review board centers, Supplementary Tables 1–8 and protocol document. [file 41591_2026_4323_MOESM1_ESM.pdf]

nature medicine

Supplementary information https://doi.org/10.1038/s41591-026-04323-8

Gotistobart or docetaxel in metastatic squamous non-small cell lung cancer: stage 1 of the randomized phase 3 PRESERVE-003 trial

In the format provided by the authors and unedited

List of Institutional Review Board Centres

Australian Centres

- Mater Hospital
- Newcastle Private Hospital
- Cancer Research SA
- Bankstown-Lidcombe Hospital
- Campbelltown, NSW
- Sunshine Hospital
- Hollywood Private Hospital, WA

Canadian Centres

- Saskatchewan Cancer Agency (SCA)
- Sasquatches-wan Cancer Agency- Saskatoon Cancer Centre

China Centres

- Affiliated Hospital of Hebei University
- Anhui Medical University - The Second Hospital
- Cancer Hospital Chinese Academy of Medical Sciences
- Chengdu Seventh People's Hospital
- China-Japan Friendship hospital
- Chongqing University Cancer Hospital (Chongqing Cancer Institute)
- Dongguan People's Hospital
- Fujian Medical University Union Hospital
- Guangdong Provincial People's Hospital
- Hainan General Hospital (Hainan Provincial People's Hospital)
- Henan Cancer Hospital
- Hubei Cancer Hospital
- Hunan Cancer Hospital
- Jinan Central Hospital
- Renmin Hospital of Wuhan University
- Shanxi Bethune Hospital
- Sichuan Cancer Hospital
- Sir Run Run Shaw Hospital, Zhejiang University, School of Medicine
- The Affiliated Hospital of Xuzhou Medical University
- The Affiliated Tumor Hospital of Harbin Medical University
- The First Affiliated Hospital of Anhui Medical University
- The First Affiliated Hospital of Guangdong Pharmaceutical University
- The First Affiliated Hospital of Xi'an Jiaotong University
- The First Affiliated Hospital of Zhengzhou University
- The People's Hospital of Guangxi Zhuang Autonomous Region
- The Second Affiliated Hospital of Nanchang University
- The Second Affiliated Hospital of Soochow University
- The Second Affiliated hospital of Zhejiang University School of Medicine
- Tianjin Medical University Cancer Institute & Hospital
- Tongji University - Shanghai Oriental Hospital (Shanghai East Hospital)
- Union Hosp. Tongji Medical College Huazhong Uni. of Sci. & Tech.
- Weifang Second People's Hospital
- West China Hospital of Sichuan University
- Xiamen University - The First Affiliated Hospital
- Xiangya Hospital, Central South University
- Zhejiang Cancer Hospital
- Zhujiang Hospital of Southern Medical University

South Korean Centres

- Asan Medical Centre
- Chungbuk National University Hospital
- Keimyung University Dongsan Hospital
- Korea University Guro Hospital
- National Cancer Center
- Severance Hospital, Yonsei University Health System
- The Catholic University of Korea, St. Vincent's Hospital

United Kingdom Centres

- Barts Health NHS Trust
- Cambridge University Hospitals NHS Foundation Trust
- Royal Devon University Healthcare NHS Foundation Trust
- South Tees Hospital
- The Christie NHS Foundation Trust
- Torbay Hospital – Horizon Centre
- University Hospitals Birmingham NHS Foundation Trust (Queen Elizabeth Hospital)

United States of America Centres (covered by central WCG IRB Study Number: 20230079)

- Advent Health
- AMR LLC
- Astera Cancer Care
- Bass Medical Group
- Cancer Care Associates of York
- Cotton O'Neil Clinical Research Center
-
- D & H Cancer Research Center
- Deaconess Research Institute
- Donald Guthrie Foundation
- Emad Ibrahim M.D. Inc.
- Florida Cancer Center North
- Florida Cancer Specialists – East
- Florida Cancer Specialists – South
- Fort Wayne Medical Oncology
- Genesis Cancer & Blood (LA) SCRI
- Genesis Cancer Ctr
- Jefferson City MG
- Messino Cancer Centers
- Miami Valley Hospital
- MultiCare Institute for Research and Innovation Tacoma
- NHO-Revive Research Institute, LLC
- Norton Cancer Institute
- Norwalk Hospital/Nuvance Health
- Ocala Oncology
- Ohio State University
- Oklahoma Cancer Specialists
- Oncology Associates of Oregon
- Oncology Consultants, P.A.
- Oncology Research, HealthPartner Institute
- Orchard Healthcare Research Inc.
- Orlando Health
- Pennsylvania Cancer Specialists Research Institute
- SCRI - Tennessee Oncology
- Springfield Clinic, LLP
- Texas Oncology
- Texas Oncology – Northeast Texas-Tyler
- Texas Oncology – San Antonio
- Texas Oncology- McAllen /USOR
- The Oncology Institute (Cerrito, CA)
- Tri County Hematology-Oncology
- U of Cincinnati
- UC Davis
- University of Kentucky
- University of Tennessee
- Virginia Cancer Institute
- Virginia Cancer Specialists, PC
- Virginia Oncology Associates
- XCancer/ Dothan Hematology & Oncology

Supplementary Table 1. Baseline patient demographics and disease characteristics in patients with non-sqNSCLC

Characteristic		Gotistobart (n=58)	Docetaxel (n=62)
Median age (range) — years		68 (36–86)	64 (45–91)
Sex — no. (%)	Male	38 (65.5)	44 (71.0)
	Female	20 (34.5)	18 (29.0)
Race — no. (%)	Asian	30 (51.7)	37 (59.7)
	White	22 (37.9)	19 (30.6)
	Black or African American	4 (6.9)	5 (8.1)
	Other	2 (3.4)	1 (1.6)
Region — no. (%)	US	21 (36.2)	21 (33.9)
	Non-US*	37 (63.8)	41 (66.1)
ECOG PS score — no. (%)	0	14 (24.1)	16 (25.8)
	1	44 (75.9)	46 (74.2)
Smoking status — no. (%)	Never	12 (20.7)	16 (25.8)
	Current	7 (12.1)	6 (9.7)
	Former	39 (67.2)	40 (64.5)
Median (range) time from initial diagnosis to randomization — months		10.4 (0.5–59.7)	11.9 (0.8–75.3)
PD-(L)1 TPS — no. (%)	PD-(L)1 <1%	23 (39.7)	24 (38.7)
	PD-(L)1 1–49%	14 (24.1)	13 (21.0)
	PD-(L)1 ≥50%	8 (13.8)	13 (21.0)
	Unknown	13 (22.4)	12 (19.4)
Metastases at baseline — no. (%)	Liver	16 (27.6)	6 (9.7)
	Brain	17 (29.3)	20 (32.3)
Number of prior lines of therapy in the advanced/metastatic setting — no. (%)	1	42 (72.4)	45 (72.6)
	2	13 (22.4)	12 (19.4)
	≥3	3 (5.2)	5 (8.1)
Prior anticancer therapy — no. (%)	Radiotherapy	32 (55.2)	24 (38.7)
	Surgery	18 (31.0)	25 (40.3)
	Platinum chemotherapy	58 (100.0)	60 (100.0)
	Anti-PD-(L)1	58 (100.0)	62 (100.0)
	CTLA-4 therapy	1 (1.7)	1 (1.6)

*Non-US included patients recruited from Australia, China, Korea, and the United Kingdom.
CTLA-4, cytotoxic T-lymphocyte-associated protein 4; ECOG PS, Eastern Cooperative Oncology Group performance status; non-sqNSCLC, non-squamous non-small cell lung cancer; PD-(L)1, programmed cell death protein/programmed death ligand 1; TPS, tumor proportion score; US, United States.

Supplementary Table 2. Subsequent anticancer systemic therapy in patients with sqNSCLC

**Anticancer therapy — no. (%)**		**Gotistobart (n=45)**	**Docetaxel (n=42)**
Any anticancer systemic therapy		18 (40.0)	26 (61.9)
PD-(L)1 inhibitors (pembrolizumab, sugemalimab, serplulimab, camrelizumab, sintilimab, tislelizumab)		3 (6.7)	10 (23.8)
Chemotherapy	Pyrimidine analogues	7 (15.6)	8 (19.0)
	Platinum compounds	6 (13.3)	9 (21.4)
	Taxanes	9 (20.0)	3 (7.1)
	Combinations	1 (2.2)	0
	Podophyllotoxin derivatives	0	1 (2.4)
	Vinca alkaloids	0	1 (2.4)
	Topoisomerase I inhibitor	0	1 (2.4)
Other protein kinase inhibitors and EGFR inhibitors (catequentinib, afatinib, surufatinib)		8 (20.0)	13 (31.0)
Monoclonal antibodies and antibody–drug conjugates		2 (4.4)	2 (4.8)
VEGF/VEGFR inhibitors		2 (4.4)	0
Other investigational or unspecified traditional medicines		0	4 (9.5)
Other antineoplastic agents		1 (2.2)	3 (7.1)
Interleukins		0	1 (2.4)
CD2 targeted monoclonal antibody		0	1 (2.4)

CD2, cluster of differentiation 2; EGFR, epidermal growth factor receptor; PD-(L)1, programmed cell death protein/programmed death ligand 1; sqNSCLC, squamous non-small cell lung cancer; VEGF, vascular endothelial growth factor; VEGFR, vascular endothelial growth factor receptor.

Supplementary Table 3. Efficacy summary for patients with non-sqNSCLC (n=120) receiving gotistobart vs. docetaxel and patients with mixed histology who received gotistobart 3 mg/kg (n=10)

**Endpoint**	**Patients with non-sqNSCLC (n=120)**		**Mixed histology**
	Gotistobart 6 mg/kg with 2 × 10 mg/kg loading doses, Q3W (n=58)	Docetaxel (n=62)	Gotistobart 3 mg/kg Q3W (N=10)*
OS events — no.	34	29	9
Median OS — months [95% CI]	8.9 [4.0, 12.6]	12.9 [10.4, NE]	3.8 [1.9, 15.8]
HR [95% CI]	1.7 [1.0, 2.7]		N/A
12-month OS rate — % [95% CI]	38.5 [25.3, 51.6]	58.2 [43.7, 70.2]	30.0 [7.1, 57.8]
PFS events — no.	46	40	9
Median PFS — months [95% CI]	2.6 [2.1, 3.6]	5.0 [3.8, 8.0]	1.9 [1.3, 2.2]
HR [95% CI]	2.3 [1.4, 3.7]		N/A
12-month PFS rate — % [95% CI]	0.0 [NE, NE]	21.2 [10.3, 34.6]	0
Confirmed ORR — no. [%; 95% CI]	5 [8.6; 2.9, 19.0]	5 [8.1; 2.7, 17.8]	0
Best overall response — no. (%)			
Complete response	0	0	0
Partial response	5 (8.6)	5 (8.1)	0
Stable disease	22 (37.9)	31 (50.0)	2 (20.0)
Progressive disease	17 (29.3)	13 (21.0)	5 (50.0)
NE	0	0	1 (10.0)
Missing	14 (24.1)	13 (21.0)	2 20.0)
Median DoR — months [95% CI]	5.5 [3.8, NE]	6.6 [2.7, NE]	N/A

*Six patients with non-sqNSCLC and four patients with sqNSCLC.
CI, confidence interval; DoR, duration of response; HR, hazard ratio; N/A, not applicable; NE, not evaluable; non-sqNSCLC, non-squamous non-small cell lung cancer; ORR, objective response rate; OS, overall survival; PFS, progression-free survival;
Q3W, every 3 weeks; sqNSCLC, squamous non-small cell lung cancer.

Supplementary Table 4. Summary of treatment-emergent adverse events in patients with sqNSCLC

Event — no. (%)	Gotistobart (N=45)		Docetaxel(N=41)	
Serious adverse events	34 (75.6)		16 (39.0)	
TEAEs leading to discontinuation	10 (22.2)		2 (4.9)	
Pneumonia	3 (6.7)		0	
Hemoptysis	2 (4.4)		0	
Pulmonary tuberculosis	1 (2.2)		0	
Colitis	1 (2.2)		0	
Diarrhea	1 (2.2)		0	
Immune-mediated enterocolitis	1 (2.2)		0	
Immune-mediated lung disease	1 (2.2)		0	
Cardiac failure	1 (2.2)		0	
ALT increased	1 (2.2)		0	
AST increased	1 (2.2)		0	
Blood alkaline phosphatase increased	1 (2.2)		0	
Fatigue	0		1 (2.4)	
Febrile neutropenia	0		1 (2.4)	
Decreased appetite	0		1 (2.4)	
Poor quality sleep	0		1 (2.4)	
TEAE leading to death	2 (4.4)		0	
	Any grade	Grade ≥3	Any grade	Grade ≥3
Any TEAEs	45 (100.0)	30 (66.7)	40 (97.6)	26 (63.4)
Most common TEAEs				
Diarrhea	17 (37.8)	2 (4.4)	6 (14.6)	0 (0.0)
ALT increased	16 (35.6)	3 (6.7)	4 (9.8)	0 (0.0)
AST increased	15 (33.3)	2 (4.4)	2 (4.9)	0 (0.0)
Pyrexia	13 (28.9)	0 (0.0)	1 (2.4)	0 (0.0)
Anemia	12 (26.7)	1 (2.2)	17 (41.5)	2 (4.9)
Weight decreased	11 (24.4)	0 (0.0)	3 (7.3)	0 (0.0)
Pneumonia	11 (24.4)	7 (15.6)	8 (19.5)	2 (4.9)
Decreased appetite	11 (24.4)	0 (0.0)	7 (17.1)	1 (2.4)
Nausea	10 (22.2)	0 (0.0)	6 (14.6)	0 (0.0)
Hypoalbuminemia	10 (22.2)	0 (0.0)	6 (14.6)	0 (0.0)
Chills	10 (22.2)	1 (2.2)	0 (0.0)	0 (0.0)
Platelet count decreased	9 (20.0)	1 (2.2)	4 (9.8)	0 (0.0)
Vomiting	9 (20.0)	0	3 (7.3)	0
Hyponatremia	9 (20.0)	0	5 (12.2)	0
Blood lactate dehydrogenase increased	8 (17.8)	0	1 (2.4)	0
Upper respiratory tract infection	8 (17.8)	0	4 (9.8)	0
Cough	8 (17.8)	0	10 (24.4)	0
Dyspnea	8 (17.8)	0	4 (9.8)	0
Infusion-related reaction	8 (17.8)	0	0	0
Constipation	7 (15.6)	0	6 (14.6)	0
Pruritus	7 (15.6)	0	0	0
Insomnia	7 (15.6)	0	0	0
Fatigue	6 (13.3)	1 (2.2)	7 (17.1)	0
Rash	6 (13.3)	1 (2.2)	1 (2.4)	0
Neutrophil count decreased	5 (11.1)	0	11 (26.8)	10 (24.4)
Blood alkaline phosphatase increased	5 (11.1)	0	0	0
Colitis	5 (11.1)	4 (8.9)	0	0

White blood cell count decreased	4 (8.9)	0	10 (24.4)	7 (17.1)
Alopecia	0	0	7 (17.1)	0

ALT, alanine aminotransferase; AST, aspartate aminotransferase; sqNSCLC, squamous non-small cell lung cancer; TEAE, treatment-emergent adverse event.

Supplementary Table 5. Summary of immune-related adverse events in patients with sqNSCLC (>5% of patients)

Immune-related adverse events — no. (%)	Gotistobart (n=45)		Docetaxel (n=41)	
	Any grade	Grade ≥3	Any grade	Grade ≥3
Any	27 (60.0)	15 (33.3)	4 (9.8)	2 (4.9)
Diarrhea	10 (22.2)	2 (4.4)	0	0
Colitis	5 (11.1)	4 (8.9)	0	0
Increased ALT	5 (11.1)	2 (4.4)	0	0
Increased AST	4 (8.9)	2 (4.4)	0	0
Immune-mediated lung disease	3 (6.7)	2 (4.4)	0	0
Rash	3 (6.7)	1 (2.2)	0	0
Decreased appetite	3 (6.7)	0	0	0
Nausea	3 (6.7)	0	0	0
Pruritis	3 (6.7)	0	0	0

ALT, alanine aminotransferase; AST, aspartate aminotransferase; sqNSCLC, squamous non-small cell lung cancer.

Supplementary Table 6. Summary of TRAEs assessed by investigators in patients with non-sqNSCLC

Adverse events — no. (%)	Gotistobart (n=57)		Docetaxel (n=56)	
	Any grade	Grade ≥3	Any grade	Grade ≥3
Any	47 (82.5)	24 (42.1)	45 (80.4)	18 (32.1)
Any serious adverse event	25 (43.9)	-	7 (12.5)	-
Adverse events in ≥10% of patients in either treatment group				
Diarrhea	13 (22.8)	5 (8.8)	8 (14.3)	2 (3.6)
Infusion-related reaction	13 (22.8)	2 (3.5)	1 (1.8)	0
ALT increased	10 (17.5)	1 (1.8)	2 (3.6)	0
Anemia	9 (15.8)	2 (3.5)	13 (23.2)	3 (5.4)
Chills	9 (15.8)	0	1 (1.8)	0
Colitis	9 (15.8)	7 (12.3)	0	0
AST increased	8 (14.0)	0	0	0
Pruritus	8 (14.0)	0	2 (3.6)	0
Pyrexia	7 (12.3)	0	0	0
Decreased appetite	6 (10.5)	0	4 (7.1)	0
Nausea	6 (10.5)	0	9 (16.1)	0
Rash	6 (10.5)	0	5 (8.9)	0
Fatigue	5 (8.8)	0	11 (19.6)	2 (3.6)
Neutrophil count decreased	3 (5.3)	1 (1.8)	9 (16.1)	8 (14.3)
Edema peripheral	2 (3.5)	0	10 (17.9)	1 (1.8)
White blood cell count decreased	1 (1.8)	0	7 (12.5)	3 (5.4)
Alopecia	0	NA	19 (33.9)	NA
Paraesthesia	0	0	7 (12.5)	0
Peripheral sensory neuropathy	0	0	6 (10.7)	1 (1.8)

ALT, alanine aminotransferase; AST, aspartate aminotransferase; NA, not applicable; non-sqNSCLC, non-squamous non-small cell lung cancer; TRAE, treatment-related adverse event.

Supplementary Table 7. Summary of TEAEs in patients with mixed histology who received a 3 mg/kg

**Event — no. (%)**	**Gotistobart 3 mg/kg**	
	Any grade	Grade ≥3
Any serious adverse events	8 (80.0)	
Any serious TRAEs	3 (30.0)	
TEAEs	10 (100.0)	8 (80.0)
TRAEs	8 (80.0)	3 (30.0)
TEAEs in at least two patients		
Weight decreased	4 (40.0)	0
Tri-iodothyronine decreased	3 (30.0)	0
Chronic obstructive pulmonary disease	3 (30.0)	3 (30.0)
Anemia	3 (30.0)	1 (10.0)
ALT increased	3 (30.0)	0
AST increased	3 (30.0)	0
Dyspnea	2 (20.0)	2 (20.0)
Pneumonia	2 (20.0)	2 (20.0)
Decreased appetite	2 (20.0)	1 (10.0)
Hyperglycemia	2 (20.0)	1 (10.0)
Blood creatinine increased	2 (20.0)	0
Cough	2 (20.0)	0
Diarrhea	2 (20.0)	0
Hypoalbuminemia	2 (20.0)	0
Hyponatremia	2 (20.0)	0
Platelet count decreased	2 (20.0)	0
Pyrexia	2 (20.0)	0

ALT, alanine aminotransferase; AST, aspartate aminotransferase; TEAE, treatment-emergent adverse event; TRAE, treatment-related adverse event.

Supplementary Table 8. Protocol-recommended management plan for selected gotistobart-related adverse events

<table>
<tr><th></th><th>Grade 1</th><th>Grade 2</th><th>Grade 3</th><th>Grade 4</th></tr>
<tr><td colspan="5">Diarrhea/colitis</td></tr>
<tr><td>Grade</td><td>Diarrhea: <4 stools/day over baseline
Colitis: asymptomatic</td><td>Diarrhea: 4–6 stools per day over baseline; IV fluids indicated <24 hours; limiting instrumental ADL
Colitis: abdominal pain; blood in stool</td><td>Diarrhea: ≥7 stools per day over baseline; IV fluids ≥24 hours; limiting self-care ADL
Colitis: severe abdominal pain; peritoneal signs</td><td>Life-threatening, perforation</td></tr>
<tr><td>Management</td><td>• Continue gotistobart therapy per protocol
• Initiate symptomatic treatment
• Close monitoring for worsening symptoms
• Educate the patient to report worsening immediately
If worsens:
• Treat as Grade 2 or Grade 3–4</td><td>• Delay gotistobart therapy per protocol
• Initiate symptomatic treatment
• 1–2 mg/kg/day methylprednisolone or oral equivalent
• When symptoms improve to Grade 1, taper steroids over at least 1 month, consider prophylactic antibiotics for opportunistic infections, and resume gotistobart therapy per protocol
If worsens or persists >2– 3 days with oral steroids:
• Treat as Grade 3–4</td><td colspan="2">• Withhold gotistobart therapy per protocol
• 1–2 mg/kg/day methylprednisolone IV or IV equivalent
• Add prophylactic antibiotics for opportunistic infections
• Consider lower endoscopy
If persists >3–5 days, or recurs after improvement*
• Add 300 mg/dose of vedolizumab at 0, 2, 6, and 14 weeks
• Consider testing CMV for steroid-refractory colitis
If improves:
• Continue steroids until Grade 1, then taper over at least 1 month
• Add one dose of 300 mg vedolizumab within one week of resuming gotistobart treatment per protocol</td></tr>
</table>

	Grade 1	Grade 2	Grade 3	Grade 4
**Liver function abnormalities and hepatitis**				
**Grade[†]**	ALT >ULN to 3 × ULN and/or total bilirubin >ULN to 1.5 × ULN	ALT >3 × to 5 × ULN and/or total bilirubin >1.5 × to 3 × ULN	ALT >5 × ULN to 20 × ULN and/or total bilirubin >3 × ULN to 10 × ULN	ALT >20 × ULN and/or total bilirubin >10 × ULN
**Management**	• Continue gotistobart therapy per protocol<br>• Consider increasing the frequency of monitoring<br>• Continue LFT monitoring per protocol<br>If worsens:<br>• Treat as Grade 2 or Grade 3–4	• Withhold gotistobart<br>• Increase frequency of monitoring to every 3 days<br>If returns to baseline:<br>• Resume routine monitoring and gotistobart therapy per protocol<br>If elevations persist >5–7 days or worsen:<br>• 0.5–1 mg/kg/day methylprednisolone or oral equivalent<br>• When ALT returns to <3 × ULN and bilirubin <1.5 × ULN, taper steroids over at least 1 month, consider prophylactic antibiotics for opportunistic infections, and resume gotistobart therapy per protocol	• Withhold gotistobart<br>• Increase frequency of monitoring to every 1–2 days<br>• 1–2 mg/kg/day methylprednisolone IV or IV equivalent[‡]<br>• Add prophylactic antibiotics for opportunistic infections<br>• Consult a gastroenterologist or hepatologist<br>If ALT returns to <5 × ULN and bilirubin <3 × ULN:<br>• Taper steroids over at least 1 month and follow the Grade 2 management.<br>If it does not improve in >3–5 days, worsens, or rebounds:<br>• Add MMF 1 g BID<br>• If no response within an additional 3–5 days, perform liver biopsy and consider tacrolimus or other immunosuppressants per local guidelines<br>Note: Avoid infliximab due to the potential risk of liver failure	

*Per Zou F, et al. *J Immunother Cancer,* 2021;9:e003277, doi:10.1136/jitc-2021-003277; [†]Although standard serum liver enzyme panels should be performed, the treatment guideline is based on serum enzyme tests of ALT, as AST elevation is less specific to hepatocyte injuries; [‡]The recommended starting dose for ALT >20 × ULN or bilirubin >10 × ULN is 2 mg/kg/day methylprednisolone IV.

ALT, alanine aminotransferase; ADL, activities of daily living; AST, aspartate aminotransferase; BID, twice-daily; CMV, cytomegalovirus; IV, intravenous; LFT, liver function test; MMF, mycophenolate mofetil; ULN, upper limit of normal.

Phase 3, Two-stage, Randomized Study of ONC-392 Versus Docetaxel in Metastatic Non-Small Cell Lung Cancers that Progressed on PD-1/PD-L1 Inhibitors

Protocol Number: PRESERVE-003

Posted on ClinicalTrials.gov as NCT05671510

Sponsor: **OncoC4, Inc.**
9640 Medical Center Drive
Rockville, MD 20850

IND Number: 143461
WCG IRB Study Number: 20230079

Protocol Version / Date: 3.2 / 16 DEC 2025

Final Protocol Approval

Signed by Pan Zheng

Signature with date: PPD

Name: Pan Zheng, MD, PhD

Designation: Chief Medical Officer

Confidentiality Statement

The information in this document is confidential and is not to be disclosed without the written consent of OncoC4 Inc. except to the extent that disclosure would be required by law and for the purpose of evaluating and/or conducting a clinical trial for OncoC4 Inc. You are allowed to disclose the contents of this document only to your Institutional Review Board or Ethics Committee and study personnel directly involved with conducting this protocol. Persons to whom the information is disclosed must be informed that the information is confidential and proprietary to OncoC4 Inc. and that it may not be further disclosed to third parties.

Study Principal Investigator:

Mark A. Socinski, MD
Executive Director
Advent Health Cancer Institute
2501 N. Orange Ave, Suite 689
Orlando, FL 32804
Email: Mark.Socinski.MD@AdventHealth.com

Study Co-Principal Investigators:

Tianhong Li, MD, PhD
UC Davis Comprehensive Cancer Center
Division of Hematology & Oncology
4501 X Street, Suite 3016
Sacramento, CA 95817
Email: thli@ucdavis.edu

Kai He, MD, PhD
The Ohio State University Comprehensive Cancer Center
Division of Hematology and Oncology
460 W. 10th Avenue
Columbus, OH 43210
Email: kai.he@osumc.edu

Protocol Acceptance Form

Protocol Title: Phase 3, Two-stage, Randomized Study of ONC-392 Versus Docetaxel in Metastatic Non-Small Cell Lung Cancers that Progressed on PD-1/PD-L1 Inhibitors

Protocol Number: PRESERVE-003

Posted on ClinicalTrials.gov as NCT05671510

Sponsor: OncoC4, Inc.
9640 Medical Center Drive
Rockville, MD 20850

IND NUMBER: 143461

WCG IRB Study Number: 20230079

Protocol Version / Date: 3.2 / 16 DEC 2025

I agree to conduct the study in accordance with the current protocol.

______________________	______________________	________________
Principal Investigator's name	Signature	Date

DOCUMENT HISTORY

CCI

[redacted]

1 STUDY SYNOPSIS

1.1 Title

Phase 3, Two-stage, Randomized Study of ONC-392 Versus Docetaxel in Metastatic Non-Small Cell Lung Cancers that Progressed on PD-1/PD-L1 Inhibitors

1.2 Indication

Patients with metastatic squamous non-small cell lung cancer (sqNSCLC) who have progressed after at least 12 weeks of PD-1 or PD-L1 inhibitor-based therapy and the therapy is the most recent line of treatment.

1.3 Summary of Rationale

There has been a paradigm shift for the treatment of metastatic NSCLC with the new targeted therapies and immune checkpoint inhibitors. For NSCLC without driver mutation, the PD-1/PD-L1 inhibitor as the first-line (monotherapy or in combination with platinum-based doublet agents) or second-line therapy after chemotherapy significantly improved overall survival. Patients who respond to the PD-1/PD-L1 inhibitor-based therapy tend to have a longer duration of response. However, there remains a significant number of patients who do not have response or have an initial response followed by disease progression to PD-1/PD-L1 inhibitor therapy. For patients with progression on PD-1/PD-L1 inhibitor, continuing a PD-1/PD-L1 inhibitor is not recommended. The options are either a platinum doublet chemotherapy if the first-line is PD-1/PD-L1 inhibitor monotherapy, or single-agent chemotherapy with or without VEGF inhibitor if they had prior platinum-based chemotherapy. The remaining options are best supportive care or enrolling in clinical trials of investigational drug candidates. Thus, the shift in the early line treatment to immunotherapy (IO) or chemoimmunotherapy also exposes the largest unmet medical need in metastatic NSCLC.

ONC-392 is a highly selective, humanized monoclonal immunoglobulin G1 (IgG1)-kappa isotype antibody against CTLA-4 with a robust anti-tumor activity and lower autoimmune toxicity in animal studies in comparison to ipilimumab. ONC-392 dissociates from CTLA-4 under low pH in endosomes to allow both CTLA-4 and ONC-392 to escape from lysosomal degradation and recycle to the cell surface. Unlike ipilimumab that down-regulates CTLA-4 expression on Treg cells, ONC-392 keeps high level CTLA-4 on Treg cells through this recycling mechanism and makes Treg cells a better target for antibody-dependent cellular cytotoxicity, particularly in the tumor microenvironment (TME) where macrophages are more abundant. The selective elimination of Treg cells in the tumor microenvironment and maintenance of CTLA-4 expression in Treg cells in the peripheral tissues by ONC-392 form the cellular and molecular basis for more potent tumor rejection and low toxicity in animal studies.

In the ongoing Study PRESERVE-001, 184 patients had at least one dose of ONC-392 treatment as of 31AUG2022. There were 56 NSCLC patients enrolled. The tumor response has been observed in patients with NSCLC, ovarian cancer, anal cancer, adenoid cystic carcinoma (ACC), and HNSCC after receiving ONC-392 monotherapy. Tumor response was also observed in patients with triple negative breast cancer, cervical cancer, NSCLC and melanoma after receiving ONC-392 in combination with pembrolizumab. [redacted]

In the monotherapy expansion cohort part of the study (N=143), 36 (25%) patients experienced grade 3 treatment-related AEs (TRAEs) including diarrhea/colitis/immune-mediated enterocolitis (10.5%) and hepatitis/immune-mediated hepatitis (2.1%). Other grade 3 TRAEs were recorded in one or two patients

(<2%). One patient had a grade 4 TRAE of shock (0.7%), and none had grade 5 TRAEs. Seven patients (5.9%) discontinued the treatment due to TRAEs.

Taken together, the data from pre-clinical and clinical studies as well as the significant unmet medical need lend support of continued development of ONC-392 in patients with NSCLC who progressed on PD-1/PD-L1 inhibitor-based therapy.

1.4 Study Design

**ABBREVIATED TITLE**	ONC-392 vs Docetaxel in PD-1 resistant NSCLC
**TRIAL PHASE**	Phase 3
**CLINICAL INDICATION**	Patients with metastatic squamous NSCLC who have progressed after at least 12 weeks of PD-1 or PD-L1 inhibitor-based therapy and the therapy is the most recent line of treatment.
**TRIAL TYPE**	Interventional
**TYPE OF CONTROL**	Active control with chemotherapy agent Docetaxel
**ROUTE OF ADMINISTRATION**	Intravenous
**TRIAL BLINDING**	Unblinded, Open-label
**TREATMENT ARMS**	Arm 1: ONC-392 6 mg/kg Q3W with two loading doses of 10 mg/kg Q3W<br>Arm 2: Docetaxel 75 mg/m^2 Q3W
**TREATMENT PERIOD**	Up to 17 cycles (approximately 1 year) with the option to continue treatment beyond 1 year.
**NUMBER OF TRIAL SUBJECTS**	CCI [redacted]
**RANDOMIZATION RATIO**	1:1
**ESTIMATE DURATION OF TRIAL**	From first patient first visit to last patient last visit: approximately 52 months.<br>Subject accrual period: 37 months. Each patient will be treated up to 17 cycles, approximately 1 year.
**PRIMARY OBJECTIVES**<br>1. To assess the efficacy of ONC-392 vs. docetaxel as measured by overall survival (OS).	**PRIMARY ENDPOINT**<br>1. Overall survival (OS)
**SECONDARY OBJECTIVES**<br>1. To assess the efficacy of ONC-392 vs. docetaxel by objective response rate (ORR) and progression-free survival (PFS).	**SECONDARY ENDPOINT**<br>1. Objective response rate (ORR) as assessed by Investigator per RECIST 1.1<br>2. Progression-free survival (PFS) as assessed by Investigator per RECIST 1.1

2. To assess safety and tolerability of ONC-392 vs. docetaxel.	3. Incidence of TEAEs, TRAEs, irAEs, and AEs leading to treatment discontinuation.
**EXPLORATORY OBJECTIVES**	**EXPLORATORY ENDPOINTS**
1. To compare DCR, DOR, and other efficacy parameters between ONC-392 and docetaxel.<br>2. To characterize ONC-392 PK and exposure-relationship.<br>3. To characterize Anti-ONC-392 antibody and its effect on ONC-392 PK, efficacy and safety.<br>4. To evaluate health-related quality-of-life (HRQoL) using the European Organisation for Research and Treatment of Cancer (EORTC) QoL-Core 30 (QLQ-C30), EORTC Lung Cancer-Specific Quality-of-Life Questionnaire (QLQ-LC29), Non-small Cell Lung Cancer Symptom Assessment Questionnaire (NSCLC-SAQ), and EuroQol-5 dimension-5 level (EQ-5D-5L).	1. Disease control rate (DCR), duration of response (DOR) and best overall response (BOR) by Investigator per RECIST 1.1<br>2. Population PK of ONC-392<br>3. Exposure-response relationship for efficacy and safety<br>4. ADA and its effect on ONC-392 PK, efficacy, and safety<br>5. HRQoL endpoints:<br>a. Change from baseline in EORTC QLQ-C30 Global Health Status/Quality-of-Life scale, functional scales, and symptom scales/item score.<br>b. Change from baseline in coughing, shortness of breath, side-effects, tumor progression/existential issues, surgery-related symptoms scales and single-item scores of the QLC-LC29.<br>c. Change from baseline in NSCLC-SAQ total score and domain score (pain, dyspnea, cough, appetite, fatigue).<br>d. Change from baseline in EQ Visual Analog Scale Score and EQ 5D-5L index score.
**TRIAL DESIGN**	After the DMC meeting in August 2024, the study design has been updated to adapt the recommendation from DMC (see **Section 2.2.8** for detail). The primary analysis population of the trial will include all squamous NSCLC patients enrolled in Stage 2, which is defined as patients not included in Stages 1A or 1B.<br><br>This is a randomized, open-label, active-controlled, Phase 3 study. The study population consists of patients with metastatic squamous NSCLC who progressed on PD-1/PD-L1 inhibitor. Approximately [redacted] patients will be enrolled in the entire study (**Figure 1**), [redacted]. Stage 1A patients are defined as those who were randomized in the 1:1:1 three-arm randomization stage for dose selection. Stage 1B includes a) patients that were randomized after Stage 1A and included in Aug 2024 DMC-initiated unplanned futility analysis, and b) non-squamous NSCLC patients randomized after Stage 1A. Data from squamous NSCLC patients in Stage 1A and 1B will be used as supportive evidence to strengthen the primary analysis results.<br><br>[redacted]

	CCI [redacted]<br><br>Unless otherwise specified, number of patients and number of events specified in this protocol are for Stage 2 patients.<br><br>All enrolled patients who are randomized to the ONC-392 arm will receive ONC-392 for up to 17 cycles in approximately 1 year or until discontinuation criteria are met. Patients who are randomized to the docetaxel arm will receive docetaxel 75 mg/m^2 Q3W for up to 17 cycles in approximately 1 year or until disease progression per RECIST 1.1.<br><br>Treatment response will be evaluated with radiographic imaging by investigator based on RECIST 1.1. The complete response (CR), partial response (PR), or disease progression (PD) should have a confirmative imaging 4 - 6 weeks from initial imaging. Post-PD treatment is allowed if investigator considers there is potential clinical benefit to the patient.<br><br>Safety including adverse events (AEs) will be monitored throughout the trial. Patients who complete the 17-cycle treatment or discontinue study treatment will remain in the study and enter the follow-up period to be monitored for disease progression (PD) and survival. For patients who are off the treatment due to reasons other than PD, every effort should be made to continue monitoring disease status by tumor imaging Q9±1W in first year and Q16±3W in second year until PD event occurs. Patients will be contacted by telemedicine Q16±3W until the patient's death or the end of the study, whichever occurs first.<br><br>The health-related quality-of-life (HRQoL) will be evaluated using the European Organisation for Research and Treatment of Cancer (EORTC) QoL-Core 30 (QLQ-C30), EORTC Lung Cancer-Specific Quality-of-Life Questionnaire (QLQ-LC29), Non-small Cell Lung Cancer Symptom Assessment Questionnaire (NSCLC-SAQ), and EuroQol-5 dimension-5 level (EQ-5D-5L).
**ELIGIBILITY**<br><br>**<u>Major Inclusion Criteria (refer to Section 4.1 for details and additional criteria)</u>**<br><br>1) Adult (≥ 18 years), all genders, capable of signing informed consent.<br><br>2) Histologically- or cytologically-confirmed diagnosis of metastatic squamous NSCLC, metastasis can	**<u>Major Exclusion Criteria (refer to Section 4.2 for details and additional criteria)</u>**<br><br>1) Cancer treatment-related AEs have not recovered to NCI CTCAE grade ≤ 1 except endocrinopathy.<br><br>2) Last anti-PD-1/PD-L1 dosing within 28 days prior to first dose of study treatment.<br><br>3) Receiving systemic steroid therapy with >10 mg/day prednisone or equivalent within 7 days prior to the first dose of study treatment.

be regional lymph nodes or distant organs.<br>3) Radiographic progression after treatment with the most recent line of treatment being either 3a or 3b:<br>a. At least 12 weeks of PD-1/PD-L1 inhibitor in combination with platinum-based chemotherapy;<br>b. Prior treatment with at least 2 cycles of a platinum-based chemotherapy, followed by at least 12 weeks of standard doses of PD-1 or PD-L1 inhibitor-based immunotherapy.<br>Antibodies against CTLA-4, LAG-3, TIGIT, VEGF or VEGFR in combination with PD-1/PD-L1 inhibitor are allowed.<br>4) At least one measurable tumor lesion according to RECIST 1.1.<br>5) ECOG score of 0 or 1.<br>6) Adequate organ functions. Serum LDH level ⩽ 2xULN.<br>7) Life expectancy ⩾ 3 months.	4) Having non-squamous histology type or documented targetable mutations or genomic alterations in any of the following genes: EGFR, ALK, ROS1, HER2, MET, BRAF, RET or NTRK. Exception: KRAS mutations are not excluded.<br>5) Patients who have symptomatic brain metastasis. Palliative radiotherapy or radiosurgery to brain metastasis within 14 days of the first dose of study drug.<br>6) Active GI disease, including peptic ulcer disease, pancreatitis, diverticulitis, or inflammatory bowel disease.<br>7) Active interstitial lung disease (ILD) or non-infectious pneumonitis.<br>8) Active infections with IV antibiotics within 14 days prior to first dose of study treatment.<br>9) Impaired heart function.
**STATISTICAL CONSIDERATION**	**Sample size justification:**<br>The study plans to enroll [redacted] patients, including non-squamous patients and squamous patients that have already been randomized to the ONC-392 3 mg/kg arm, ONC 392 6 mg/kg arm with two loading doses of 10 mg/kg, or docetaxel arm in Stage 1A, Stage 1B and Stage 2. [redacted].<br>In this study, unless otherwise specified, all analyses specified in this document are based on the sqNSCLC patients that are randomized in Stage 2. Details for the analyses including patients randomized in Stage 1A and 1B will be specified in the SAP.

	[redacted]
**DATA MONITORING COMMITTEE**	An independent data monitoring committee (DMC) will review the results [redacted] and make recommendations to the Sponsor. The composition and procedures of the DMC will be described in detail in the DMC Charter.

1.5 Study Schedule

Table 1. Schedule of Assessments

**Schedule of Events**	**Screening [1]**	**Cycle 1**	**Cycles 2-17**	**EOT [2]**	**Post Treatment**
**Cycle Day**	**Up to Day -28**	**Day 1 [3]**	**Day 1 [3]**	**At Treatment Discontinuation**	**Survival FU [2]**
**Visit Number**	**V1**	**V2**	**V3 to V18**		**Q16W**
**Visit interval ± Window (Days)**	**-28 to -1**	**0±3**	**21±3**	**1-30 (+7)**	**112±21**
Informed consent [1]	X				
Inclusion/Exclusion Criteria	X				
Demographics, Medical History, smoking history, Prior Medication [4]	X				
Weight and Height[5]	X	X	X		
Vital Signs [5]	X	X (infusion day)	X (infusion day)	X	
Directed Physical Examination	X	As clinically needed.		X	
ECOG Performance Status	X			X	
ECG	X			X	
Review Adverse Events [6]		X	X	X	
Review Concomitant Medications [6]	X		X	X	
CBC with differential [7]	X	X[1]	X	X	
Comprehensive Serum Chemistry Panel [7]	X	X[1]	X	X	
Thyroid Function [7,8]	X		X		
LDH [7]	X	X[1]	X		
β-HCG test for WOCBP [7]	X		X		
PK, ADA, biomarker, and ctDNA sampling [9]		X	X		
Fresh Tumor Biopsies (optional) [10]	X		X (Week 8)		
Randomization [11]	X				
**Study Drug Administration (Note)**		X	X		
Tumor Imaging and RECIST Assessment[12]	X		X		X[2]
Brain MRI [12]	X		As needed		

Post-EOT new anti-cancer treatment					X[2]
Survival assessment					X[2]
EORTC QLQ-C30, EORTC QLQ-LC29, NSCLC-SAQ, EQ 5D-5L[13]		X	X	X	X

C#D# = Cycle # Day # (e.g., C1D1 = Cycle 1 Day 1); ADA = anti-drug antibody; CBC = complete blood count; CT = computerized tomography; ctDNA = circulating tumor DNA; ECG= electrocardiogram; ECOG = Eastern Cooperative Oncology Group; EORTC = European Organisation for Research and Treatment of Cancer; EOT = end of treatment; FU= Follow-up; IV = intravenous; LDH = lactate dehydrogenase; MRI = magnetic resonance imaging; NCI CTCAE = National Cancer Institute Common Terminology Criteria for Adverse Events; NSCLC-SAQ = Non-Small Cell Lung Cancer Symptom Assessment Questionnaire; OS = overall survival; PD= disease progression; PFS = progression-free survival; PK =pharmacokinetic; T3 = triiodothyronine; T4 = thyroxine; TSH = thyroid stimulating hormone; WOCBP = women of childbearing potential; EORTC QLQ-C30 = EORTC Core Quality of Life questionnaire; EORTC QLQ-LC29 = EORTC Quality of Life Questionnaire-Lung Cancer 29; NSCLC-SAQ = NSCLC Symptom Assessment Questionnaire; EQ 5D-5L = EuroQol 5 Dimension 5 Level.

NOTE: ONC-392 IV infusion is given over a period of approximately 60 minutes. Pre-medication should be given 30 to 60 minutes before the first dosing. For cycle 1, the post dosing observation time is 60 ± 20 minutes after the end of infusion. For Cycle 2 to Cycle 17, if there is no infusion reaction observed in prior cycle, the observation time is a minimum of 15 minutes. A cycle is 21 ± 3 days. National holidays and long weekends may extend the cycle days. Patients may be required to withhold treatment due to adverse events as clinically needed. Docetaxel IV infusion is given over a period of approximately 60 minutes. Please follow the docetaxel product insert for premedication regimen and administration procedure.

1. Screening: Written consent must be obtained prior to performing any protocol-specific procedure. Screening tests should be performed within 28 days of C1D1. Results of a test performed as part of routine clinical management are acceptable in lieu of a screening test if performed within 28 days prior to C1D1. If serum chemistry and CBC with differential are done within 10 days to C1D1, the results can be used for both screening and C1D1. If these two tests are done more than 10 days to C1D1, the results should be entered as screening and C1D1 pre-dosing blood samples should be collected for serum chemistry and CBC with differential. If local institution guideline allows, C1D1 hematology, chemistry, LDH, pregnancy test can be done within 3 days to C1D1. NOTE: Hematology, chemistry, LDH, and serum HCG samples can be performed locally or sent to the study central lab for testing for the screening and on treatment visit timepoints.
2. EOT visit should be conducted within 30 + 7 days when the decision to discontinue the study treatment is made. Next line of anti-cancer treatment regimen should be recorded. Survival follow-up includes both progression-free survival (PFS) and overall survival (OS). (1) Patients who discontinue treatment due to PD should enter the survival follow-up (Q16 ±3W) by chart review or telemedicine or visit. Next line anti-cancer treatment regimen should be recorded. (2) Patients who complete 17 cycles (approximately 1 year) of study treatment, their EOT visit will be performed within 30 days after Cycle 17 followed by PFS/survival follow-up (Q16 ±3W) by CT scans, chart review or telemedicine or visit. (3) Patients who discontinue the study treatment due to an AE (without documented PD) will have safety follow-up until the resolution of the AE to Grade 0-1 or baseline level and enter the PFS/survival follow-up period. Best effort should be made to follow the patients for PFS (by tumor imaging Q9±1W through 1 year and Q16±3W after 1 year) and for survival (Q16 ±3W) by chart review or telemedicine or visit. (4) Best effort should be made to follow the survival (Q16 ±3W) for patients who discontinue study treatment and select hospice service. All patients discontinue treatment without PD should be followed for PFS (by tumor imaging Q9±1W through 1 year and Q16±3W after 1 year) until disease progression or any new anti-cancer therapy starts. All patients will be followed for overall survival until death, withdrawal of consent to survival follow-up or the end of the study.
3. On Day 1 of every cycle, all assessments indicated should be done pre-dosing. Clinic visit and tests can be done within 3 days to drug infusion day. Vital signs and PK/ADA/Biomarker sampling should be done before the drug infusion on the same day.

4. Includes history of treatment for NSCLC, including prior systemic, radiation, and surgical treatment. Time of last PD-(L)1-based treatment period and outcome must be documented. Record smoking history. Report medication history for both cancer therapy and non-cancer therapy for 30 days prior to the screening visit (Visit 1).
5. Height will be collected at screening and weight will be collected predose every cycle. ONC-392 dose is determined by screening body weight unless there are more than 10% changes in body weight, which requires dose adjustment. Docetaxel dose is determined by screening body surface area by calculation through body weight and height. The BSA should be calculated based on the Stevenson formula [BSA (m^2) = 0.0061 x height (cm) + 0.0128 x weight (kg) -0.1529] or using locally approved formulas for BSA calculation. Vital signs including temperature, pulse, respiratory rate, and blood pressure should be recorded pre-dosing on the same day of drug infusion and at EOT. Additional vital signs including pulse and blood pressure should be recorded if there is any infusion reaction.
6. AEs and laboratory safety measurements will be graded per NCI CTCAE version 5.0. All AEs will also be evaluated for seriousness and relationship to study drug(s). AEs will be collected from the start of the study treatment through the end of treatment. All SAEs and irAEs will be collected from the start of the study treatment to 90 days after the last dose of the treatment or to the date that the patient initiates new anti-cancer therapy, whichever comes first.
7. Routine laboratory tests (CBC with differential; serum chemistry panel; thyroid function tests; LDH, serum β-HCG) should be performed by the central laboratory. Please refer to **Section 5.6** for the list of required items in each test. CBC with differential, chemistry and LDH will be performed at screening, prior to C1D1 (if tested >10 days before C1D1) and in every cycle from Cycles 2 to 6, and every even numbered cycle afterward for Cycles 8, 10, 12, 14, and 16. Serum Pregnancy test should be performed at screening (see **Appendix 1** for Pregnancy Testing). Pregnancy test will be performed whenever a menstrual cycle is missed or when pregnancy is otherwise suspected; serum β-HCG is mandatory to confirm an indeterminate test or to confirm a positive urine pregnancy test. The samples for routine lab tests should be collected and shipped to the Sponsor designated central laboratory by following the instructions in the Laboratory Manual. Local laboratory may be used for any clinically needed tests for safety monitoring.
8. TSH should be done every four cycles (screening, Cycles 4, 8, 12, 16) or Cycle 20 and every 4 cycles afterward if patient continues treatment beyond 1 year.
9. PK, ADA, and Biomarker samples should be collected at pre-dose of Cycle 1, 2, 4, 8, 12, and 16 from patients who are randomized to ONC-392 treatment arms only. ctDNA samples should be collected at pre-dose of cycle 1 from all patients. The samples should be collected and shipped to the Sponsor designated central laboratory by following the instructions in the Laboratory Manual.
10. A fresh biopsy of a tumor lesion is desirable but not mandatory. Optional consent for tumor biopsy procedure is required. Fresh biopsies should be limited to readily accessible tumor lesions (e.g., skin; peripheral lymph nodes; liver metastases which can be readily accessed using CT guidance). The study investigators must ensure that patients will not be subject to a significant risk procedure. Only core biopsy is allowed. When feasible, another tumor biopsy should be taken approximately 8 weeks after start of study therapy, to be able to compare the expression or profile of biomarkers while on study therapy versus baseline. Ideally, the follow-up biopsies should be taken from the same tumor lesion as the baseline biopsy. Tumor biopsy is preferred from a different location than target lesion(s).
11. Randomization through IWRS should only be done after the screening to confirm the eligibility. Patients should be randomized within 7 days prior to C1D1.
12. Tumor imaging (either CT or MRI, with preference for CT) is performed within 28 days prior to C1D1; and while on study, every 9±1 weeks post C1D1 in first year, and every 16±3 weeks from the most recent scan after one year. The screening images and the follow-up images should be submitted to BICR vendor for study image collection. The investigator's assessment per RECIST 1.1 should be recorded in EDC. The same imaging technique should be used for each patient throughout the study. Tumor imaging schedule follows calendar days and should not be adjusted for delays in cycle starts. The first tumor assessment should be scheduled 9±1 weeks from C1D1. CR, PR, and PD should be confirmed by repeat scan performed at 4-6 weeks after the prior

imaging. The subsequent CT scan will follow the new Q9±1W schedule from the last CT scan date in first year, and every 16±3 weeks after one year. Brain MRI is performed at screening. Brain MRI from routine medical management up to 45 days prior to C1D1 can be used as screening MRI.

13. Patients randomized under Protocol Version 2.0 and later should complete the HRQoL questionnaires in their entirety at specified timepoints throughout the study. All the questionnaires should be given to randomized patients prior to initiation of study procedures and AE evaluation.

1.6 Study Schema

Figure 1. [redacted]

[redacted]

LIST OF ABBREVIATIONS AND TERMS

Abbreviation/Term	Definition
ADA	Anti-Drug Antibody
ADCC	Antibody Dependent Cellular Cytotoxicity
AE	Adverse Event
ALT	Alanine Transaminase
ALK	Anaplastic Lymphoma Kinase
ALP	Alkaline Phosphatase
BoR	Best Overall Response
AST	Aspartate Transaminase
BICR	Blinded Independent Central Review
CBC	Complete Blood Count
CITE	Cancer Immunotherapeutic Effects
CHO	Chinese Hamster Ovary
CR	Complete Response
CSR	Clinical Study Report
CRF	Case Report Form
CT	Computed Tomography
CT/CAP	Computed Tomography on Chest, Abdomen and Pelvis.
CTLA-4	Cytotoxic T-Lymphocyte-Associated Protein 4
DLT	Dose-Limiting Toxicity
DNA	Deoxyribonucleic Acid
DoR	Duration of Response
eCRF	Electronic Case Report Form
ECG	Electrocardiogram
ECOG	Eastern Cooperative Oncology Group
EDC	Electronic Data Capture
EGFR	Epidermal Growth Factor Receptor
EOT	End of Treatment
EORTC	European Organisation for Research and Treatment of Cancer
EQ-5D-5L	EuroQoL-5 Dimension-5 Level
eTMF	Electronic Trial Master File
FDA	Food and Drug Administration
FSH	Follicle-Stimulating Hormone
Gr	Grade
HNSTD	Highest Non-Severely Toxic Dose
ICH	International Council for Harmonisation
IHC	Immunohistochemistry
ICF	Informed Consent Form
IND	Investigational New Drug
INR	International Normalized Ratio
IO	Immunotherapy
irAEs	Immunotherapy-Related Adverse Effects
IP	Investigational Product
IRB	Institutional Review Board
IV	Intravenous
IWRS	Interactive Web Response System
LDH	Lactate Dehydrogenase
mAbs	Monoclonal Antibodies

Abbreviation/Term	Definition
MMF	Mycophenolate mofetil
MRI	Magnetic Resonance Imaging
MTD	Maximum Tolerated Dose
NCCN	National Comprehensive Cancer Network
NCI	National Cancer Institute
NCI CTCAE	National Cancer Institute Common Terminology Criteria for Adverse Events
NSCLC	Non-Small Cell Lung Cancer
NSCLC-SAQ	Non-Small Cell Lung Cancer Symptom Assessment Questionnaire
ORR	Objective Response Rate
OS	Overall Survival
PBMC	Peripheral Blood Mononuclear Cell
pRBC	Packed Red Blood Cell
PD	Progressive Disease
PD-1	Programmed Cell Death Protein 1
PD-L1	Programmed Death-Ligand 1
PFS	Progression-Free Survival
PI	Principal Investigator
PK	Pharmacokinetics
PR	Partial Response
RA/EC	Regulatory authority or ethics committee
Q3W	(Once) Every 3 Weeks
RECIST	Response Evaluation Criteria in Solid Tumors
RNA	Ribonucleic Acid
RP2D-M	Recommended Phase 2 Dose for Monotherapy
SAE	Serious Adverse Event
SJS	Stevens-Johnson Syndrome
SOC	Standard of Care
sqNSCLC	Squamous Non-Small Cell Lung Cancer
T3	Triiodothyronine
T4	Thyroxine
TEAE	Treatment Emergent Adverse Event
TEN	Toxic Epidermal Necrolysis
TRAE	Treatment-Related Adverse Event
TPS	Tumor Proportion Score
TSH	Thyroid Stimulating Hormone
TME	Tumor Microenvironment
β-HCG	Beta Human Chorionic Gonadotropin
WOCBP	Women of Childbearing Potential

TABLE OF CONTENTS

1 STUDY SYNOPSIS 5
1.1 Title 5
1.2 Indication 5
1.3 Summary of Rationale 5
1.4 Study Design 6
1.5 Study Schedule 11
1.6 Study Schema 15
2 BACKGROUND AND RATIONALE 22
2.1 CTLA-4 As Target for Cancer Therapy and ONC-392 22
2.1.1 Anti-CTLA-4 Antibodies 22
2.1.2 ONC-392 22
2.1.3 ONC-392-Specific Mechanism of Action 22
2.1.4 Preliminary Safety and Efficacy Data of ONC-392 in Patients with Solid Tumors, including NSCLC 24
2.1.5 Clinical Pharmacology Analyses of Exposure/response Relationship 25
[redacted] [redacted] 25
[redacted] [redacted] 26
2.2 Rationale 28
2.2.1 Overall Study Design [redacted] 28
2.2.2 Rationale for the Trial and Selected Patient Population: Unmet Medical Need in PD-(L)1-resistant NSCLC 29
2.2.3 Rational for Use of Docetaxel as the Active Control 31
2.2.4 Rationale for Stratification Factors 31
2.2.5 Rationale for Treatment Duration 32
2.2.6 Rationale for Testing 6 mg/kg Q3W with 2 Loading Doses of 10 mg/kg Q3W 32
2.2.7 [redacted] 33
2.2.8 [redacted] 38
2.2.9 [redacted] 38
3 STUDY OBJECTIVES 40
3.1 Primary Objectives and Endpoint 40
3.2 Secondary Objectives and Endpoints 40
3.3 Exploratory Objectives and Endpoints: 40
4 ELIGIBILITY CRITERIA 42
4.1 Inclusion Criteria 42
4.2 Exclusion Criteria 43
5 STUDY METHODOLOGY, TREATMENT PLAN AND PROCEDURES 45
5.1 Study Interventions 45
5.2 Composition of the Drug Product 45
5.3 Drug Product Preparation/Handling/Storage 45
5.3.1 ONC-392 45
5.3.2 Docetaxel 46
5.4 Concomitant and Prohibited Medications/Vaccinations 46
5.4.1 Acceptable Concomitant Medications 46

5.4.2 Prohibited Concomitant Medications ... 46
5.5 Study Procedures ... 47
5.6 Enrollment/Screening Period ... 47
5.6.1 Enrollment ... 47
5.6.2 Screening Period ... 48
5.7 Treatment Allocation/Randomization ... 49
5.8 Treatment Period ... 49
5.8.1 ONC-392 IV Infusion and Post-infusion Observation ... 49
5.8.2 PK/ADA/Biomarker/ctDNA Sampling Schedule ... 49
5.9 ONC-392 Dose Adjustment ... 49
5.10 Docetaxel IV Infusion and Dose Adjustment ... 50
5.11 Local Cancer Treatment ... 50
5.12 End of Treatment (EOT) Visit and Follow-up Period ... 51
5.13 Optional Treatment after One Year ... 51
5.14 Discontinuation of Investigational Product (IP) ... 52
5.15 Lost to Follow-up ... 52
5.16 Subject Replacement Strategy ... 53
5.17 Beginning and End of the Study ... 53
5.18 Study Completion ... 53
5.19 Options for Patients Enrolled Prior to Protocol Amendment v3.0 ... 53
6 STUDY ASSESSMENTS ... 54
6.1 Efficacy Measurements ... 54
6.2 List of Safety Measurements ... 54
6.3 Efficacy Assessments ... 54
6.4 Pharmacokinetics ... 55
6.4.1 Collection of PK/ADA/Biomarker Samples and ctDNA Samples ... 55
6.4.2 Storage and Destruction of PK/ADA/Biomarker Samples ... 55
6.4.3 Chain of Custody of Biological Samples ... 55
6.4.4 Withdrawal of Informed Consent for Donated Biological Samples ... 56
6.5 Health-related Quality-of-life Questionnaires ... 56
7 ADVERSE EVENTS ... 58
7.1 Definition of Adverse Events (AEs) ... 58
7.2 Definition of Serious Adverse Events (SAEs) ... 58
7.3 Grading Adverse Events (Serious and Non-serious) ... 58
7.4 Collecting and Recording Adverse Events ... 58
7.5 Serious Adverse Event Reporting ... 60
7.6 Assessing Causality ... 61
7.7 Disease Progression ... 61
7.8 Deaths ... 61
7.9 Immune-related Adverse Event (irAE) Management Plan ... 61
7.9.1 General Management of irAEs ... 61
7.9.2 Management Recommendations of the Most Common ONC-392-related irAEs ... 62
7.9.3 Other irAEs ... 65
7.10 Recommended Prophylactic and Acute Management of Infusion Reactions ... 66
8 STATISTICAL CONSIDERATIONS ... 69
8.1 Sample Size Rationale ... 69
8.2 Statistical Analyses ... 69
8.2.1 General Considerations ... 69
8.2.2 Definition of Baseline ... 69

8.2.3 Analysis Sets 69
8.2.4 Primary Efficacy Analyses 70
8.2.5 [redacted] 70
8.2.6 Secondary Efficacy Analysis 70
8.2.6.1 Analysis of ORR 71
8.2.6.2 Analysis of PFS 71
8.2.7 Exploratory Efficacy Analyses and Subgroup Analyses 71
8.3 Safety Analyses 71
8.3.1 Adverse Event Analysis 71
8.3.2 Other Safety Analyses 72
8.4 Exploratory Analyses 72
8.4.1 Pharmacokinetic Analysis 72
8.4.2 Population Pharmacokinetics and Exposure-Response/Safety Analyses 72
8.4.3 Immunogenicity Analysis 72
8.4.4 Health-related Quality-of-life Questionnaires 72
8.4.5 Other Exploratory Analyses 72
9 STUDY OVERSIGHT AND DATA REPORTING/REGULATORY REQUIREMENTS 73
9.1 Regulatory and Ethical Considerations 73
9.2 Financial Disclosure 73
9.3 Informed Consent Process 73
9.4 Data Protection 74
9.5 Committees Structure 74
9.6 Dissemination of Clinical Study Data 74
9.7 Data Quality Assurance 75
9.8 Source Documents 75
9.9 Study and Site Start and Closure 76
9.9.1 First Act of Recruitment 76
9.9.2 Study/Site Termination 76
9.10 Publication Policy 76
9.11 Study Medication Accountability 77
10 REFERENCES 78
11 Appendix 1 84
12 Appendix 2 88
13 Summary of Changes 89

LIST OF FIGURES

Figure 1. [redacted] 15
Figure 2. ONC-392 Mechanism of Action.......... 23
Figure 3. [redacted] 27
Figure 4. [redacted] 28

LIST OF TABLES

Table 1. Schedule of Assessments 11
Table 2. Part C ONC-392 Monotherapy Treatment Regimen and Enrollment Status 24
Table 3. Summary of TEAEs [redacted] in Patients Receiving ONC-392 Monotherapy (Part A and Part C) 25
Table 4. Real-world Treatment Patterns and Outcomes in PD-1/PD-L1 Resistant NSCLC 30
Table 5. Summary of TEAEs in NSCLC Patients with ONC-392 Monotherapy 33
Table 6. [redacted] .. 35
Table 7. [redacted] 36
Table 8. [redacted] 37
Table 9. Laboratory Values Indicative of Adequate Organ Function 42
Table 10. Study Interventions 45
Table 11. Laboratory Tests 48
Table 12. ONC-392 Dose Adjustments 50
Table 13. Dose Modification Standards for Adverse Events Related to Docetaxel.......... 50
Table 14. Recommended Management Plan for ONC-392-related Diarrhea/Colitis 63
Table 15. Recommended Management Plan for ONC-392-related Liver Function Abnormalities and Hepatitis 64
Table 16. Recommended Dose Modifications for irAEs 65
Table 17. Recommended Dose Modification and Toxicity Management Guidelines on ONC-392 Associated Infusion Reactions 67

2 BACKGROUND AND RATIONALE

2.1 CTLA-4 As Target for Cancer Therapy and ONC-392

2.1.1 Anti-CTLA-4 Antibodies

Cytotoxic T-lymphocyte-associated protein 4 (CTLA-4), also known as CD152 (cluster of differentiation 152), is a cell surface protein receptor that interacts with B7-1 (CD80) and B7-2 (CD86) [1-3] to ensure proper function of regulatory T cells (Tregs) [4] and protect host against autoinflammatory diseases [5-8]. CTLA-4 is shown to be an immune checkpoint and a good target for cancer therapy in animal models [9-11].

Unlike other checkpoint inhibitors such as anti-PD-1/PD-L1 antibodies, the anti-CTLA-4 antibody, ipilimumab (YERVOY®), has gained market approval for one indication (melanoma) as a monotherapy [12, 13]. The toxicity profile significantly limits its dose and exposure that are required for achieving higher efficacy benefit [14]. The less optimal dose may explain the consistently lower response rate than an anti-PD-1 antibody in head-to-head comparison studies in melanoma [15-17] and its failure as a monotherapy in multiple Phase 3 clinical trials in other cancer indications [16]. Despite its approval for multiple cancer indication as a combination therapy with nivolumab (Opdivo®, an anti-PD-1 antibody) [12,13,15,18-20], the incidence of grade 3/4 irAEs (e.g., up to 73-90% of patients with melanoma receiving ipilimumab/nivolumab as a neo-adjuvant therapy [12,16]) remains high. CTLA-4 remains a valid and attractive immunotherapy target and anti-CTLA-4 antibodies induce long-lasting immunity in cancer patients [17,21], however, the less favorable safety profile significantly limits its clinical usage.

The molecular basis underlying irAEs and CITE of anti-CTLA-4 antibodies is traditionally viewed as antagonizing the endogenous function of CTLA-4 [14,22]. In both mice and humans, genetic inactivation of CTLA-4 caused severe autoimmune diseases [5-7]; therefore, an effective antagonist of CTLA-4 is likely to induce autoimmune diseases. If inactivation of CTLA-4 is necessary, then irAEs would be a necessary price for cancer immunity.

Recent studies have questioned whether inactivation of CTLA-4 function contributes to cancer immunity. It was reported that blocking the interaction between CTLA-4 and its ligands, CD80 and CD86, is neither necessary nor sufficient for CITE of anti-CTLA-4 antibodies [23]. Furthermore, studies from several laboratories established that selective depletion of Tregs in the tumor microenvironment (TME) but not in normal tissues is the primary mechanism of action (MOA) of CITE [14,23-26]. This new understanding of selective Treg depletion explains why it is possible to uncouple irAEs from CITE [14,27].

2.1.2 ONC-392

The chemical name of ONC-392 is Immunoglobulin G1 (IgG1), anti-human CTLA-4 (antigen), human γ1-chain, disulfide with human κ-chain, dimer [28]. It is a humanized recombinant IgG1 kappa immunoglobulin. The IgG1 isotype was chosen because of its strong ADCC activity. Additional mutations of the Fc were introduced to further enhance the ADCC activity and *in vivo* half-life [29].

The International Non-proprietary Name (INN) and the proposed United States Adopted Name (USAN) is gotistobart. Gotistobart is jointly developed by OncoC4 and BioNTech.

2.1.3 ONC-392-Specific Mechanism of Action

In human *CTLA4* knock-in mice, ipilimumab induced more severe autoimmune disease with the pathology highly similar to patients treated with the antibody, whereas ONC-392 was associated with much reduced toxicity [23]. In mouse with large tumor burden, the mouse version of ONC-392 was more effective in tumor rejection than ipilimumab [30].

It is well-established that CTLA-4 recycles between the cell surface and endosomes [31], where it is prevented from lysosomal degradation and recycles back to the cell surface by binding to the lipopolysaccharide-responsive and beige-like anchor (LRBA) protein [8,32]. Genetic mutations in either *CTLA4* [8,32] or *LRBA* [8,32] cause autoimmune diseases in human. As illustrated in **Figure 2**, the irAE-prone, pH-insensitive anti-CTLA-4 antibodies (e.g., ipilimumab) induce lysosomal degradation of CTLA-4, disrupt CTLA-4 recycling to the surface, and lead to development of autoimmune toxicity. In contrast, ONC-392 as a pH-sensitive anti-CTLA-4 antibody dissociates from CTLA-4 in endosomes, allows normal recycling of both antibodies and CTLA-4, which lead to a much-reduced autoimmune toxicity [30]. The preservation of the recycling of both CTLA-4 and ONC-392 facilitates more potent ADCC to eliminate Treg cells in the tumor microenvironment and induces strong CITE in mice that bear large established tumors.

Figure 2. ONC-392 Mechanism of Action

[redacted]

The properties of clinically used antibodies are shown on the left: the pH-insensitive antibodies cause down-regulation of cytotoxic T-lymphocyte-associated protein 4 (CTLA-4) through lysosomal degradation. In host tissues, CTLA-4 down-regulation causes autoimmune diseases; while in the tumor, CTLA-4 down-regulation reduces ADCC activity and thus anti-cancer efficacy. In contract, as depicted on the right, pH-sensitive antibodies do not cause CTLA-4 degradation as it allows recycling of both antibodies and CTLA-4 molecules. Preserving the CTLA-4 checkpoint in host tissues prevents autoimmune diseases, while preserving CTLA-4 in the tumor ensures high ADCC ligand density, and thus better regulatory T cell (Treg) depletion and anti-tumor activity.

2.1.4 Preliminary Safety and Efficacy Data of ONC-392 in Patients with Solid Tumors, including NSCLC

Study PRESERVE-001 is a Phase I/II study that consists of Part A monotherapy dose escalation study, Part B combination therapy with pembrolizumab dose escalation study, Part C cohort expansion study, and Part D a monotherapy study of patients with adenoid cystic carcinoma.

In Part A, 4 patients received ONC-392 at 3 mg/kg dose (including 2 with rapid-intra-patient dose escalation to the 3 mg/kg dose) and 6 patients received ONC-392 at 10 mg/kg dose. No DLTs or grade 3 or 4 AEs were observed in the DLT observation period at any doses tested. Based on the study findings, the RP2D for the ONC-392 monotherapy was determined to be 10 mg/kg q3w.

In Part C monotherapy cohort expansion of the study, a total of 143 patients received at least one dose of ONC-392 monotherapy as of the data cutoff date of 31AUG2022 in 8 treatment arms, including pancreatic cancer, triple negative breast cancer, NSCLC w/ targetable mutations, PD-(L)1-resistant NSCLC, melanoma, head and neck squamous cell carcinoma (HNSCC), ovarian cancer, and other solid tumors. The treatment regimen and enrollment status are listed in **Table 2**.

Table 2. Part C ONC-392 Monotherapy Treatment Regimen and Enrollment Status

[redacted]

A total of 153 patients in combined Part A and Part C of Study PRESERVE-001 received at least 1 dose of ONC-392 monotherapy at the data cutoff of 31AUG2022 (**Table 3**). Most patients (88%) experienced TEAEs and 67% experienced treatment-related TEAEs (TRAEs). The most common TRAEs (≥10%) were diarrhea/colitis/immune-mediated enterocolitis (22%), infusion-related reaction (21%), rash (15%), pruritus (12%), and fatigue (12%). Grade 3 TRAEs were reported in 26% of the patients. The most frequent Grade 3 TRAEs were diarrhea/colitis/immune-mediated enterocolitis (11%) and hepatitis/immune-mediated hepatitis (2.0%). Other significant TRAEs that observed in 1 patient (0.7%) each were: acute kidney injury, adrenal insufficiency, Guillain-Barre syndrome, immune-mediated hepatitis, immune-mediated pancreatitis, myocarditis, pneumonitis, and tubulointerstitial nephritis.

Across treatment arms in Part C, the incidence of grade 3 TRAEs was lowest in the PD-(L)1-resistant NSCLC cohort (Arm I: 13%). The primary tumors in abdomen had higher incidence of grade 3 TRAEs, such as ovarian cancer (Arm L: 43%) and pancreatic cancer (Arm A: 43%). A total of 20 (13%) patients

discontinued the study treatment due to TEAEs, of whom 9 (6%) due to TRAEs. 10 patients (6.5%) died of TEAEs. No death occurred due to TRAEs.

Of the 153 patients enrolled in Part A and Part C, 47 were NSCLC patients and 24 (2 from Part A and 22 from Part C Arm I) had PD-1/PD-L1 treatment as last line of treatment before enrollment who would have met the eligibility criteria for this study. As of the data cutoff date, a total of 12 patients were evaluable. Of note, 2 of the evaluable patients participated in Part A of the study and received ONC-392 at the 10 mg/kg dose q3w for 4 cycles, and the rest 10 patients in Part C received ONC-392 at the 10 mg/kg dose q3w for the first 2 cycles, followed by 6 mg/kg q3w for the rest treatment. [redacted]

Table 3. Summary of TEAEs [redacted] in Patients Receiving ONC-392 Monotherapy (Part A and Part C)

		Tx #1	Tx #2	Tx #3	Tx #4	
	**3 mg/kg**	**6 mg/kg Q3W**	**10 mg/kg Q3w**	**10 mg/kg Q4W**	**10 mg/kgx2 + 6 mg/kg**	**Total**
	**(N=4)**	**(N=1)**	**(N=41)**	**(N=34)**	**(N=73)**	**(N=153)**
Any Grade TEAEs	4 (100.0%)	1 (100.0%)	36 (87.8%)	31 (91.2%)	62 (84.9%)	134 (87.6%)
≥G3 TEAEs	1 (25%)	1 (100.0%)	27 (65.9%)	21 (61.8%)	43 (58.9%)	93 (60.8%)
Any Grade Related to ONC-392	2 (50.0%)	1 (100.0%)	34 (82.9%)	22 (64.7%)	43 (58.9%)	102 (66.7%)
G3 TRAEs	0	1 (100.0%)	15 (36.6%)	7 (20.6%)	16 (21.9%)	39 (25.5%)
G4 TRAEs	0	0	1 (2.4%)	0	1 (1.4%)	2 (1.3%)
Any SAEs	1 (25%)	1 (100.0%)	24 (58.5%)	19 (55.9%)	42 (57.5%)	87 (56.9%)
SAEs Related to ONC-392	0	1 (100.0%)	14 (34.1%)	4 (11.8%)	15 (20.5%)	34 (22.2%)
TRAEs leading to Dose Reduction	0	0	2 (4.9%)	1 (2.9%)	4 (5.5%)	7 (4.6%)
TRAEs leading to Permanent Discontinuation	0	1 (100.0%)	5 (12.2%)	1 (2.9%)	2 (2.7%)	9 (5.9%)
Death Related to ONC-392	0	0	0	0	0	0
[redacted]	[redacted]	[redacted]	[redacted]	[redacted]	[redacted]	[redacted]

The safety and clinical activity of ONC-392 from the ongoing Study PRESERVE-001 support continued development of ONC-392 for the treatment of PD-(L)1-resistant NSCLC.

2.1.5 Clinical Pharmacology Analyses of Exposure/response Relationship

CCI [redacted]

[redacted]

[redacted] [redacted]

[redacted]

[redacted]

CCI

[redacted]

[redacted]

2.2 Rationale

2.2.1 Overall Study Design [redacted]

[redacted]

This is a multi-center, randomized, open-label, active-controlled, Phase 3 study assessing the efficacy and safety of ONC-392 vs docetaxel in patients with metastatic squamous NSCLC who progressed on PD-(L)1-based therapy. This study is designed in accordance with ICH E6(R2) and E10 guidelines, and FDA guidance on "Cancer clinical trial eligibility criteria: available therapy in non-curative settings" [33]. The study will assess the safety and efficacy of ONC-392 versus docetaxel in treatment of PD-1 resistant squamous NSCLC. CCI [redacted]

All enrolled patients who are randomized to the ONC-392 arms will receive ONC-392 for up to 17 cycles in approximately 1 year or until discontinuation criteria are met. Patients who are randomized to the docetaxel arm will receive docetaxel 75 mg/m^2 Q3W for up to 17 cycles in approximately 1 year or until disease progression per RECIST 1.1.

Treatment response will be evaluated every 9±1 weeks post C1D1 with radiographic imaging by investigators. Investigator assessment will determine overall response rate (ORR) based on RECIST 1.1. The complete response (CR), partial response (PR), or disease progression (PD) should have a confirmative imaging 4 – 6 weeks from initial imaging.

Safety including adverse events (AEs) will be monitored throughout the trial. Patients who complete the 17-cycle treatment or discontinue study treatment will remain in the study and enter the follow-up period to be monitored for disease progression (PD) and survival. For patients who are off the treatment due to reasons other than PD, every effort should be made to continue monitoring disease status by tumor imaging Q9±1W in the first year and Q16±3W in the second year until PD event occurs. Patients will be contacted by telemedicine Q16±3W until the patient's death or end of the study, whichever occurs first.

2.2.2 Rationale for the Trial and Selected Patient Population: Unmet Medical Need in PD-(L)1-resistant NSCLC

The past decade has witnessed a paradigm shift in cancer treatment, with the advent of novel therapeutic approaches that target or manipulate the immune system (immunotherapy) [34] demonstrating unprecedented results in solid tumors.

As of today, several anti-PD-1 (nivolumab, pembrolizumab, cemiplimab), anti-PD-L1 (atezolizumab, durvalumab, avelumab) antibodies, and a combination of anti-PD-1 and anti-CTLA-4 antibodies (nivolumab and ipilimumab) have been approved as first line therapy for metastatic NSCLC [34]. The anti-PD-(L)1 therapies as the first line or second line therapy after chemotherapy significantly improved overall survival (OS). Patients who respond to the immunotherapy tend to have longer duration of response [35-37]. However, despite the advancement in the shift of the treatment paradigm, there remains unmet medical need in this population. The response rates of 30-60% as the front-line treatment and ~20% as the second line treatment also translate into a significant number of patients who are expected to have no response to the treatment or have initial response followed by relapse with subsequent disease progression [38,39]. Thus, the shift in the first-line treatment to an anti-PD-(L)1 therapy also exposes the largest unmet medical need in the post-immunotherapy setting.

The current standard of care for patients who have progressed on a checkpoint inhibitor is chemotherapy, either a platinum-based doublet, if they are chemotherapy naïve, or a single-agent chemotherapy. Chemotherapy remains the only option in the absence of targetable mutations for these patients, other than best supportive care or clinical trials [40,41]. Several retrospective studies showed that the PFS and OS were ~4 months and ~7 months, respectively in the patients who received salvage chemotherapy after

progression on previous immune checkpoint inhibitors [38]. Similar OS of 4.6-12.8 months for the second-line setting and 2.8-12.0 months for the third-line setting was reported in meta-analysis of real-world observation studies [39,42].

A recent publication analyzed the real world treatment patterns and outcomes among patients with metastatic NSCLC previously treated with PD-1/PD-L1 inhibitors [43]. Depending on how PD-1/PD-L1 inhibitors were applied, the median OS for sequent chemotherapy varied from 6 to 9 months (**Table 4**). Cohort 1 patients (N=242) received PD-1/PD-L1 inhibitor monotherapy as the first line treatment without prior exposure to chemotherapy and majority of the patients (82%) had PD-L1 TPS score ⩾ 50%. Cohort 2 patients (N=145) received first line PD-1/PD-L1 inhibitor in combination with chemotherapy. Cohort 3 (N=674) patients received platinum-containing doublets first, then second line PD-1 monotherapy. Cohort 2 and Cohort 3 had more patients with low (1-49%) or negative (<1%) PD-L1 TPS scores.

The poor prognosis of the post-PD-1/PD-L1 patient population demands better treatments. Based on median survival of 5.7-7.8 months, it is proposed that a 10.7-month survival might be a meaningful threshold for null hypothesis in clinical trial setting for this population [43].

CTLA-4 is a validated target for the treatment of NSCLC and targeting CTLA-4 in combination with PD-1 inhibition (ipilimumab + nivolumab) with or without platinum-doublet chemotherapy is an approved first-line treatment for metastatic NSCLC. Targeting CTLA-4 has been tested as a second-line therapy for NSCLC patients who failed anti-PD-(L)1 therapy. Tremelimumab, an anti-CTLA-4 antibody, in combination with durvalumab, an anti-PD-L1 antibody, was studied in 58 patients with sqNSCLC who progressed on prior PD-1 therapy [44]. The results showed a response rate of 3.4% (7% in primary resistant patients [2/28] and 0% in acquired resistant patients [0/30]), a median PFS of ~2.1 months, and a median OS of ~7.6 months after receiving the combination therapy. The minimal activity of the treatment highlighted a significant challenge in the development of second-line immunotherapy for advanced NSCLC post PD-1 therapy.

Table 4. Real-world Treatment Patterns and Outcomes in PD-1/PD-L1 Resistant NSCLC

Cohort, N	PD-1/PD-L1 Tx	Time on PD-1/PD-L1 Tx (Median, IQR in months)	Index Treatment Pattern (%)	rwOS (median, 95% CI, months)	rwPFS (range of median in different tx)
Cohort 1 (N=242)	**1L Monotherapy**<br>Pembro (78%)<br>Nivo (21%)<br>Atezo (1%)	5.0 (2.9-8.8)	Platinum-based chemo combination (39%)<br>PD-1/PD-L1 based therapy (28%)<br>Single-agent chemo (15%)<br>VEGFi + chemo (17%)	9.2 (7.5 – 12.1)	4.1 – 6.0 months
Cohort 2 (N=145)	**1L PD-1/PD-L1 combination**<br>Pembro (99%)<br>Nivo (1%)	4.8 (2.8-7.6)	VEGFi + chemo (32%)<br>Single-agent chemo (28%)<br>PD-L1 based therapy (22%)	6.4 (5.3 – 7.6)	2.6 – 3.4 months
Cohort 3 (N=674)	**2L PD-1/PD-L1 monotherapy**<br>Pembro (11%)	4.3 (2.8-7.8)	Single-agent chemo (45%)<br>VEGFi + chemo (23%)	7.2 (6.4 – 7.8)	3.2 – 4.0 months

	Nivo (84%)<br>Atezo (5%)		Platinum-based chemo (13%)		

Data from reference [43]. Cohorts 2 and 3 are the targeted population of the current trial.

In this protocol, the study population includes patients with metastatic sqNSCLC who progressed during or after PD-1/PD-L1 inhibitor treatment in standard dosage for at least 12 weeks. PD-1/PD-L1 inhibitor treatment must be the most recent line of treatment. Patients must have prior platinum-based chemotherapy, either in combination with PD-1/PD-L1 inhibitor or as prior line of treatment before PD-1/PD-L1 inhibitor therapy. The study population will be consistent with cohorts 2 and 3 in **Table 4**, including those who progressed on front-line PD-1/PD-L1 inhibitors in combination with chemotherapy and those who had prior platinum-based doublets, followed by second-line PD-1/PD-L1 inhibitor monotherapy. The study population will exclude patients who have PD-1/PD-L1 inhibitor monotherapy as the only prior line of treatment (corresponding to cohort 1 in **Table 4**) as this population may benefit from platinum-based doublet chemotherapy.

The study entry criteria specified in the protocol aim to have the study population to be representative of the patient population in a real-world setting and the trial results to be generalizable while ensuring that patients enrolled are in a reasonable physical condition to tolerate treatment and have a better chance to respond to study treatment. The study requires that all patients must have received platinum-based systemic treatment prior to or with PD-1/PD-L1 therapy to be consistent with the current treatment guidelines. Due to the difference in survival prognosis among the histology types of NSCLC reported [45,46], the study excludes the NSCLC patients with non-squamous histology type or with documented targetable genomic mutations in the following genes: EGFR, ROS1, MET, BRAF, RET, or NTRK; or other targetable genomic alterations of ALK or HER2, as there is no evidence yet suggesting that these patients are responsive to CTLA-4-targeting therapy.

The selection of the study population is also supported by the data from Study PRESERVE-001 demonstrating that an [redacted] evaluable patients who had PD-1/PD-L1 inhibitor therapy as the most recent line of treatment.

2.2.3 Rational for Use of Docetaxel as the Active Control

The current standard of care for the trial targeted patients who have progressed on prior chemotherapy and checkpoint inhibitor is chemotherapy [40,41]. Chemotherapy remains largely the only option for these NSCLC patients without targetable mutations. Docetaxel is indicated for the treatment of locally advanced or metastatic NSCLC after failure of prior platinum-based chemotherapy. In two randomized trials, docetaxel at a dose of 75 mg/m^2 was tolerable and yielded a favorable outcome in overall survival and tumor response in patients previously treated with platinum-based chemotherapy [47].

Moreover, docetaxel continues to be the reference for the evaluation of other investigational agents for the 2nd line treatment of NSCLC, including PD-1/PD-L1-resistant patients. Docetaxel is being used as a control in the randomized, controlled Phase 3 trials of other agents as a second- or third-line therapy in patients with NSCLC (e.g., SAPPHIRE/NCT03906071, CONTACT-01/NCT04471428, LEAP-008/ NCT03976375, and DUBLIN-3/NCT02504489). Therefore, using docetaxel in the control arm of this study would make the comparison clinically meaningful and the results across different trials for the same indication are comparable.

2.2.4 Rationale for Stratification Factors

From Protocol Version 3.0 forth, the randomization will be stratified by presence of brain metastases (yes or no), ECOG score (0 vs. 1), and region (US and ex-US).

NSCLC consists of two major types of cancer by histopathology: adenocarcinoma and squamous cell carcinoma. Starting from Protocol Version 3.0, the study will enroll squamous NSCLC patients only [45,46].

Brain metastases are a common complication in NSCLC. Presence of brain metastases is shown to be a poor prognostic feature in NSCLC [46]. Although most patients with advanced NSCLC and brain metastases are eligible to receive a PD-1/PD-L1 inhibitor therapy in the first-line or later setting, either alone or in combination with chemotherapy, the efficacy data of ICIs for brain metastases in NSCLC patients is limited to draw a more definitive conclusion. In this study, patients with symptomatic brain metastasis or leptomeningeal disease are excluded. Stratifying patients with stable and asymptomatic brain metastases will ensure a balanced distribution of the patients in the active and control arms for proper safety and efficacy assessment.

ECOG performance score has been shown to be an independent predictor of overall survival in patients with advanced cancer [48] and of the response to cancer therapy [49].

The trial is planned to be conducted globally in the US, Canada, Europe, Australia, South Korea and China. Stratification by geographic region (US vs. ex-US) aims to control the potential difference in therapeutic response due to the differences in race, heterogeneity in disease, and standard of care.

2.2.5 Rationale for Treatment Duration

The treatment duration of 12 months (approximately 17 cycles) is planned for the study with survival follow-up. Patients may be allowed to continue the treatment beyond 12 months if in the opinion of the Investigator that continued treatment would have clinical benefit and no safety concerns (see Section 5.13). The treatment duration is supported by the MOA of ONC-392 that depletion of T regulatory cells in the tumor microenvironment is key to the anti-tumor activity and continued ONC-392 treatment may be required to prevent regeneration of T regulatory cells and to have a sustained clinical benefit. It is anticipated that most patients are able to tolerate the treatment. Our current data showed that only 6% patients in the ongoing ONC-392 monotherapy in Study PRESERVE-001 discontinued the study due to TRAEs. The ongoing ONC-392 drug development will allow us to assess the optimal ONC-392 treatment duration.

2.2.6 Rationale for Testing 6 mg/kg Q3W with 2 Loading Doses of 10 mg/kg Q3W

The selection of the 6 mg/kg Q3W with 2 loading doses of 10 mg/kg Q3W was based on exposure/response analysis for both safety and efficacy and on the safety and efficacy data accumulated from Study PRESERVE-001. CCI [redacted]. Since most of the objective responses was achieved in the first tumor assessment while the study drug related toxicity occurs throughout the entire dosing period, it is likely a loading dose of 10 mg/kg followed by 6 mg/kg may result in high ORR with manageable safety. The more rapid response may be essential for the study population which has median survival between 6.4 and 7.2 months [43]. This notion is supported by the accumulating data in the Part A and Part C of the PRESERVE-001 trial.

In Part A dose escalation and Part C dose expansion cohorts, the safety and efficacy of ONC-392 monotherapy were evaluated in cancer patients in 5 different dosing regimens with different levels of exposure, namely:

1. 3 mg/kg Q3W
2. 6 mg/kg Q3W
3. 10 mg/kg Q3W
4. 10 mg/kg Q4W
5. 6 mg/kg Q3W with 2 loading doses of 10 mg/kg Q3W

At the interim data cut of 31AUG2022, across different indications, the pooled analysis of 153 patients by dosing regimen (**Table 3**) showed a better safety profile for the 6 mg/kg Q3W with two 10 mg/kg loading doses regimen in term of grade ⩾3 TRAEs and treatment-related SAEs, as well as TRAEs leading to dose reduction or treatment discontinuation, as compared to the regimen of 10 mg/kg Q3W. Moreover, safety data from 35 patients with NSCLC treated with ONC-392 at 6 mg/kg with two 10 mg/kg loading doses (12 from Arm C and 23 from Arm I as in **Table 2**) demonstrated a favorable safety profile with a grade ⩾3 TRAE incidence of 11.4% (**Table 5**). There were 3 NSCLC patients in Part A with ONC-392 10 mg/kg Q3W. All three patients had 4 cycles of 10 mg/kg treatment. Two of the three patients had either Gr 3 immune colitis or Gr 3 immune pancreatitis.

Table 5. Summary of TEAEs in NSCLC Patients with ONC-392 Monotherapy

		Tx #2	Tx #3	Tx #4	
	**3 mg/kg Q3W (N=1)**	**10 mg/kg Q3W (N=3)**	**10 mg/kg Q4W (N=8)**	**6 mg/kg + 10 mg/kgx2 loading (N=35)**	**Total (N=47)**
Any Grade TEAEs	1 (100.0%)	3 (100.0%)	6 (75.0%)	29 (82.9%)	39 (83.0%)
≥G3 TEAEs	0	2 (66.7%)	4 (50.0%)	21 (60/0%)	27 (57.4%)
Any Grade related to ONC-392	0	2 (66.7%)	4 (50.0%)	18 (51.4%)	24 (51.1%)
≥G3 TRAEs	0	2 (66.7%)	2 (25.0%)	4 (11.4%)	8 (17.0%)
Any SAEs	0	2 (66.7%)	4 (50.0%)	19 (54.2%)	25 (53.2%)
SAEs Related to ONC-392	0	2 (66.7%)	2 (25.0%)	4 (11.4%)	8 (17.0%)
TRAEs leading to Dose Reduction	0	0	0	0	0
TRAEs leading to Permanent Discontinuation	0	1 (33.3%)	1 (12.5%)	2 (5.7%)	4 (8.5%)
Death Related to ONC-392	0	0	0	0	0

CCI [redacted] Therefore, 10 mg/kg x 2 followed by 6 mg/kg Q3W is chosen as one of the two doses in the Stage I.

2.2.7

CCI

[redacted]

CCI

[redacted]

CCI

[redacted]

CCI

[redacted]

CCI

[redacted]

CCI [redacted]

3 STUDY OBJECTIVES

3.1 Primary Objectives and Endpoint

Primary objective:

1. To assess the efficacy of ONC-392 vs. docetaxel in treatment of squamous NSCLC patients who progressed on PD-1/PD-L1 inhibitor therapy as measured by overall survival (OS).

Primary endpoint:

1. Overall survival (OS).

3.2 Secondary Objectives and Endpoints

Secondary Objectives:

1. To assess the efficacy of ONC-392 vs. docetaxel in treatment of squamous NSCLC patients who progressed on PD-(L)1 blockade as measured by objective response rate (ORR) and progression-free survival (PFS).
2. To assess safety and tolerability of ONC-392 vs. docetaxel in squamous NSCLC patients who progressed on PD-(L)1 blockade.

Secondary endpoints:

1. Objective response rate (ORR) as assessed by investigator per RECIST 1.1.
2. Progression-free survival (PFS) as assessed by investigator per RECIST 1.1.
3. Incidence of TEAEs, TRAEs, irAEs, and AEs leading to treatment discontinuation.

3.3 Exploratory Objectives and Endpoints:

Exploratory Objectives:

1. To compare DOR, DCR and other efficacy parameters between ONC-392 and docetaxel as assessed by Investigator per RECIST 1.1
2. To characterize ONC-392 PK and exposure-response relationship
3. To characterize ADA and its effect on ONC-392 PK, efficacy, and safety
4. To evaluate health-related quality-of-life (HRQoL) using the European Organisation for Research and Treatment of Cancer (EORTC) QoL-Core 30 (QLQ-C30), EORTC Lung Cancer-Specific Quality-of-Life Questionnaire (QLQ-LC29), Non-small Cell Lung Cancer Symptom Assessment Questionnaire (NSCLC-SAQ), and EuroQol-5 dimension-5 level (EQ-5D-5L).

Exploratory endpoints may include:

1. DOR, DCR and BOR by Investigator per RECIST 1.1
2. Population PK of ONC-392
3. Exposure-response relationship for efficacy and safety
4. ADA and its effect on ONC-392 PK, efficacy, and safety
5. HRQoL endpoints:
    a. Change from baseline in EORTC QLQ-C30 Global Health Status/Quality-of-Life scale, functional scales, and symptom scales/item score.

b. Change from baseline in coughing, shortness of breath, side-effects, tumor progression/existential issues, surgery-related symptoms scales and single-item scores of the QLC-LC29.

c. Change from baseline in NSCLC-SAQ total score and domain score (pain, dyspnea, cough, appetite, fatigue).

d. Change from baseline in EQ Visual Analog Scale Score and EQ 5D-5L index score.

4 ELIGIBILITY CRITERIA

4.1 Inclusion Criteria

1. Adult, all genders, age ≥ 18 years (or per country definition of adulthood) on the day of signing informed consent, voluntarily agree to participate by signing written informed consent.
2. Histologically- or cytologically- confirmed diagnosis of metastatic squamous NSCLC, metastasis can be regional lymph nodes or distant organs.
3. Radiographic disease progression after treatment with the most recent line of treatment being 3a or 3b as follows:
   a. At least 12 weeks of standard doses of PD-1/PD-L1 inhibitor in combination with platinum-containing doublet chemotherapy;
   b. Prior treatment with at least 2 cycles of a platinum-containing doublet chemotherapy, followed by at least 12 weeks of standard doses of PD-1 or PD-L1 inhibitor-based immunotherapy.

   Antibodies against CTLA-4, LAG-3, TIGIT, VEGF or VEGFR in combination with PD-1/PD-L1 inhibitor are allowed.
4. Patient must have measurable disease lesions according to RECIST 1.1.
5. Patient must have a ECOG performance status 0 or 1.
6. Patient must have adequate organ function as indicated by the laboratory values in Table 9.

Table 9. Laboratory Values Indicative of Adequate Organ Function

System	Laboratory Value
**Hematological**	
Absolute neutrophil count (ANC)	≥ 1,500 / mm^3
Platelets	≥ 100,000 / mm^3
Hemoglobin[a]	≥ 9 g/dL or ≥5.6 mmol/L
**Renal**	
Creatinine clearance as calculated per Cockcroft-Gault or MDRD formula	≥ 30 ml/minute
**Hepatic**	
Serum total bilirubin	≤ ULN or ≤1.5 x ULN in patient with liver metastasis
Aspartate transaminase (AST) and alanine transaminase (ALT)	≤ 3X ULN or ≤5 x ULN in patients with liver metastasis
Alkaline phosphatase	≤ 2.5X ULN or ≤5 x ULN in patients with liver metastasis
**Chemistry**	
LDH	≤ 2 X ULN

[a] Criteria must be met without packed red blood cell (pRBC) transfusion within last 2 weeks. Patients can be on stable dose of erythropoietin (⩾ approximately 3 months).

7. Patient must have life expectancy ⩾ 3 months.
8. Women of childbearing potential (WOCBP) and sexually active, must agree to use adequate and highly effective birth control starting from the screening visit (Visit 1) through 6 months after the last dose of study therapy. (See **Appendix 1** for definition of WOCBP and birth control methods)
9. Male patients, if sexually active, must agree to use adequate and highly effective methods of contraception starting from the screening visit (Visit 1) through 6 months after the last dose of study therapy. (See **Appendix 1** for contraception methods).

4.2 Exclusion Criteria

A patient meeting any of the following criteria is not eligible to participate in this study:

1. Patients who have not recovered to NCI CTCAE Grade 1 or better from AEs due to cancer therapeutics except chemotherapy associated peripheral neuropathy (motor or sensory), or endocrine related AE, in which recovery to ⩽ Grade 2 is allowed. Best supportive care, such as thyroxine, insulin, steroid replacement treatment and therapy for non-cancer conditions are allowed.
2. Patients who are currently enrolled in any other clinical trial testing an investigational agent or using an investigational device, or concurrently in other approved systemic anti-cancer therapy, that contains anti-PD-1/PD-L1 antibody, with the last dose of treatment within 28 days from first day of study treatment.
3. Patients who are on chronic systemic steroid therapy at doses > 10 mg/day prednisone or equivalent within 7 days of first day of study treatment. Topical steroid use is allowed.
4. Patients who have documented non-squamous histology type or with targetable mutations or genomic alterations in the following genes: EGFR, ROS1, MET, BRAF, RET, NTRK, ALK or HER2. Patients with mutation or genomic alterations in KRAS are not excluded (see **Appendix 2**).
5. Patients who have active or symptomatic brain metastases or evidence of progression within 4 weeks prior to study drug dosing. Palliative radiation or radiosurgery for brain metastases within 14 days from first day of study treatment or patients cannot achieve neurologically stable without > 10 mg daily prednisone or equivalent treatment. Patients with treated, stable brain metastases or with untreated asymptomatic brain metastases (<1 cm and without significant edema) are allowed to enroll.
6. Patients who have active GI disease, including peptic ulcer disease, pancreatitis, diverticulitis, or inflammatory bowel disease. Or patients who have abdominal fistula, bowel obstruction, GI perforation, or intra-abdominal abscess within 6 months before initiation of study treatment.
7. Patient who has any evidence of current interstitial lung disease (ILD) or non-infectious pneumonitis, or clinical significant pleural effusion within 28 days prior to C1D1, or a prior history of ILD or non-infectious pneumonitis that was associated with target therapy or ADC or radiation or anti-PD-1/PD-L1 based therapy and required high dose steroid (>60 mg/day prednisone or equivalent).
8. Patients have active infections requiring systemic IV antibiotics within 14 days, or oral antibiotics within 7 days, from first day of study treatment. Regular treatment of urinary tract infection (UTI), upper respiratory infection (URI) and/or topical treatment or prophylaxis treatment are allowed. Patients with symptomatic fungal or viral infections should be treated and symptoms must be resolved before being allowed to enroll.

9. Patients who had prior history of cardiovascular events, i.e., stroke, myocardial infarction, symptomatic pulmonary embolism, or other symptomatic ischemic event, within 6 months prior to initiation of study treatment; or history of myocarditis or congestive heart failure within 3 months prior to initiation of study treatment.
10. Patients who had history of severe hypersensitivity/infusion related reactions to ONC-392 or any of its excipients, docetaxel or to drugs formulated with polysorbate 80, or histidine buffer or trehalose dihydrate.
11. Patients who have autoimmune diseases that require immunosuppressive medications.
12. Patients who had major surgical procedure, other than for diagnosis within 4 weeks prior to initiation of study treatment or anticipate a major surgical procedure during the study. Palliative radiation therapy is allowed prior to and during study drug administration as long as there are no acute toxicities. There should be 7 days between radiation and ONC-392 or docetaxel dosing day.
13. Patients who, in the opinion of the Investigator, have a history or current evidence of any medical or psychiatric condition, therapy, or laboratory abnormality that might confound the results of the study, interfere with the patient's participation for the full duration of the study, or make study participation not in the best interest of the patient, in the opinion of the treating Investigator. Investigators should discuss the case with the Sponsor and/or study leaders.
14. Patients who are pregnant or breastfeeding.
15. Patients who have received a live virus vaccination within 28 days prior to randomization. Seasonal flu, COVID, RSV, Shingrix, or other vaccines that do not contain live virus are permitted.

5 STUDY METHODOLOGY, TREATMENT PLAN AND PROCEDURES

5.1 Study Interventions

Table 10. Study Interventions

Intervention Name	Dosage Formulation	Unit Dose Strength(s)	Route of Administration	Regimen/Treatment Period	Sourcing
ONC-392	Solution for infusion	270 mg/vial	IV infusion	Q3W for up to 17 cycles (1 yr)	OncoC4<br>(ALMAC is the vendor for storage and distribution)
Docetaxel	Solution for infusion	20 mg/vial<br>Or other dose vials.	IV infusion	Q3W for up to 17 cycles (1 yr)	Commercially available as standard of care or supplied by OncoC4 and labelled for investigational use where sites cannot locally source, if applicable.

5.2 Composition of the Drug Product

CCI [redacted]

[redacted]

[redacted]

Please refer to package insert for the composition of docetaxel [Docetaxel PI].

5.3 Drug Product Preparation/Handling/Storage

5.3.1 ONC-392

CCI [redacted]

CCI [redacted]

5.3.2 Docetaxel

CCI [redacted]

5.4 Concomitant and Prohibited Medications/Vaccinations

5.4.1 Acceptable Concomitant Medications

Patients in ONC-392 arms are permitted to use topical, ocular, intra-articular, intranasal and inhalational corticosteroids (with minimal systemic absorption). Physiologic replacement doses of systemic corticosteroids are permitted, even if > 10 mg/day prednisone equivalents. A brief course of corticosteroids for prophylaxis, premedication prior to chemotherapy, or for treatment of non-autoimmune conditions is permitted after consultation with Medical Monitor. Systemic corticosteroids are allowed to treat drug-related adverse events as per standard of care.

Patients in docetaxel arm are allowed to use all support treatment (e.g. G-CSF if judged necessary by investigator) as standard of care.

All the concomitant medications used should be recorded on the eCRF.

5.4.2 Prohibited Concomitant Medications

The following medication/treatment is not allowed during the study (unless utilized to treat a drug-related adverse event):

- Immunosuppressive agents
- Immunosuppressive doses of systemic corticosteroids (except as stated in **Section 5.4.1**)
- Any concurrent anti-neoplastic therapy, such as chemotherapy, immunotherapy, antibody drug conjugates (ADC), targeted therapy, or standard or investigational agents for treatment of NSCLC.
- Caution should be applied in the administration of over-the-counter medications and herbal preparations during the conduct of the study. Consultation with the Medical Monitor is encouraged.
- Any live virus vaccination within 28 days prior to randomization is not allowed. Seasonal flu, COVID, RSV, Shingrix, or other vaccines that do not contain live virus are permitted.
- The use of anticoagulants is known to increase the risk of gastrointestinal hemorrhage. Since gastrointestinal hemorrhage is an adverse reaction that has been observed in ONC-392, patients who require concomitant anticoagulant therapy should be monitored closely during therapy with ONC-392.
- Strong inhibitor of the CYP3A4 enzymes (e.g., ketoconazole, itraconazole, clarithromycin, atazanavir, indinavir, nefazodone, nelfinavir, ritonavir, saquinavir, telithromycin and voriconazole) should be avoided during therapy with docetaxel.
- Proton pump inhibitors (PPIs) should be stopped prior to C1D1 and during study treatment with ONC-392. Histamine H2 receptor antagonists (H2RAs) can be used to replace PPI. However,

medical decisions for treatment or prevention of drug-related adverse events are allowed after the C1D1 treatment.

- Systemic immunotherapeutic agents are generally not allowed except the following agents: TNF-alfa inhibitors (such as infliximab), alpha 4/beta 7 anti-integrins (such as vedolizumab), IL-6 inhibitor (such as tocilizumab), mycophenolate mofetil (MMF), calcineurin inhibitors as a part of AE events management are allowed.

Investigators should refer to the local product labeling of docetaxel for additional prohibited and restricted concomitant medications.

Except for the permitted procedures specified as palliative local therapies in **Section 5.11**, all other radiation therapy or surgery to any tumor lesion is not permitted during study treatment. Patients who require such non-palliative procedures must be discontinued from study treatment.

5.5 Study Procedures

The procedures for the screening, treatment period and follow-up period in this study are presented in **Section 1.5, Table 1**.

5.6 Enrollment/Screening Period

5.6.1 Enrollment

At screening/baseline (Day -28 to -1), the Investigator or suitably trained delegate will:

1. Obtain signed informed consent before any study specific procedures are performed, including separate consents for tumor biopsy in treatment, tumor biopsy procedure, and genetic research study.
2. Assign a unique patient identification (ID) number, consists of 4-digit site number and 3-digit individual number starting at 001 in each site, and go up sequentially. This number is used to identify the patient on the electronic case report forms (eCRFs).
3. Determine patient eligibility with inclusion and exclusion criteria in **Sections 4.1** and **4.2**. The eligible patients must have histologically- or cytologically- confirmed diagnosis of metastatic squamous NSCLC, metastasis can be regional lymph nodes or distant organs or consistent with unresectable locally advanced squamous NSCLC.
4. If the patient is ineligible after screening, the demographic information, primary cancer type and the reason of screen failure should be recorded in the electronic data capture (EDC) system.
5. If a patient has screen failure, the unique ID number cannot be reused.
6. Patients who withdraw consent after randomization will not be replaced. Patients who withdraw consent before randomization will be replaced, however, the assigned ID number cannot be reused.
7. In case of marginal abnormality or suspected laboratory error, hematology or chemistry testing should be repeated within the screening period to confirm the abnormality. When the reasons for screen fail are abnormal laboratory results, patients may be re-screened if the hematology or chemistry test abnormality can be corrected within 45 days. Re-screened patients are required to re-consent and a new patient identification number will be assigned.
8. Collection of patient demographic information inclusive of age, gender, and race are to be collected during screening to aid in analyzing the PK and safety data to determine if various

aspects correlate to exposure/response, however a patient has the right to decline to respond to race/ethnicity should they chose to do so.

5.6.2 Screening Period

Written informed consent and any locally required privacy act document authorization must be obtained prior to performing any protocol-specific procedures, including screening/baseline evaluations.

1. At screening, consenting patients are assessed to ensure that they meet eligibility criteria. Patients who do not meet these criteria must not be enrolled in the study. All screening and enrollment procedures will be performed according to the assessment schedules in **Table 1**, **Section 1.5**.
2. Screening/baseline evaluations may be performed over more than 1 visit. Physical examination and ECOG evaluation should be documented.
3. Medical history must include history of treatment for NSCLC, including prior systemic, radiation, and surgical treatment, and smoking history. Treatment regimen and time of last line of PD-1/PD-L1 inhibitor-based treatment and best response to treatment must be documented.
4. Report medication history for 30 days prior to the screening visit (Visit 1).
5. Screening lab tests should be done within 28 days prior to C1D1. Results of a test performed as part of routine clinical management are acceptable in lieu of a screening test if performed within 28 days prior to C1D1 except CBC with differential and the serum chemistry tests, which should be obtained within 10 days to C1D1. If CBC with differential and the serum chemistry are done more than 10 days to C1D1, the tests should be repeated with C1D1 pre-dose samples. The C1D1 treatment decision is determined by screening test results.
6. The routine laboratory tests in **Table 11** will be done by the central laboratory according to the study schedules in **Table 1**. Patient treatment and overall management decisions will be based on central laboratory data. The samples should be collected and shipped to the Sponsor designated central laboratory by following the instructions in the Laboratory Manual. Local laboratory may be used for clinically needed safety monitoring.

Table 11. Laboratory Tests

Hematology (CBC with differential)	RBC, Hb, Hct, WBC, Plt, ANC, ALC.
Serum Chemistry	AST, ALT, Alkaline Phosphatase, Glucose, Total Protein, Albumin, Total bilirubin, Creatinine, Sodium, Potassium, Chloride, LDH.
Thyroid Function	TSH (See footnote #8 in **Table 1**).
Pregnancy test	serum β-HCG

7. For women of childbearing potential (WOCBP), a pregnancy test (either urine or serum) should be performed at screening visit and at EOT visit. (Refer to **Appendix 1**). If required by regulatory authorities and/or ethics committee, a urine pregnancy test will be performed at least monthly and prior to all imaging examinations.

8. Tumor imaging, e.g., CT of chest, abdomen and pelvis (CT/CAP) or MRI, to define target lesion(s) according to RECIST 1.1 should be performed within 28 days prior to C1D1.
9. Brain MRI is required for all patients in screening. Brain MRI performed within 45 days to C1D1 may be allowed in lieu of screening brain MRI. In the case that the brain MRI scan cannot be performed (for instance in patients with implanted pacemaker), a brain CT scan is allowed.
10. A fresh biopsy of a tumor lesion is highly desirable at baseline and on treatment but not mandatory. Fresh biopsies should be limited to readily accessible tumor lesions (e.g., skin; peripheral lymph nodes, or liver metastases, which can be readily accessed using CT guidance). Biopsies of target lesions should be avoided. When feasible, another tumor biopsy should be taken approximately 8 weeks after C1D1. Ideally, the follow-up biopsy should be taken from the same tumor lesion as the baseline biopsy.

5.7 Treatment Allocation/Randomization

This is an open-label, randomized study, treatment randomization will occur centrally using the Interactive Web Response System (IWRS). Following screening, eligible participants will be randomized to receive ONC-392 or docetaxel treatment. The randomization will be performed within 7 days prior to the C1D1 visit. The randomization will be stratified by brain metastases (yes or no), ECOG score (0 or 1), and geographic region (US vs. ex-US).

5.8 Treatment Period

All procedures to be conducted during the 17 cycles (approximately 12-month) of the treatment period will be performed according to the study schedules (see **Table 1**).

5.8.1 ONC-392 IV Infusion and Post-infusion Observation

ONC-392 IV infusion will be given over a period of approximately 60 minutes. The pre-medication should be given for first treatment of ONC-392 (see **Section 7.10**) as prophylaxis on infusion reaction.

The drug product of ONC-392 has the concentration of 30 mg/mL. The drug product should be diluted with a 5% Dextrose Solution following the instruction in Pharmacy Manual.

For first treatment, patient should be closely observed for delayed infusion-related reactions and other immediate adverse event for at least 60±20 minutes post-infusion. For other cycles, the post-infusion observation time is a minimum of 15 minutes.

5.8.2 PK/ADA/Biomarker/ctDNA Sampling Schedule

PK/ADA/Biomarker/ctDNA samples will be collected at the timepoints indicated in footnote 9 of **Table 1**. All patients should have Cycle 1 pre-dose ctDNA samples. Only the patients who are randomized to ONC-392 treatment arms will have the PK/ADA/Biomarker samples collected at pre-dose at Cycle 1, 2, 4, 8, 12 and 16. The blood samples should be sent to Sponsor designated laboratory. Please refer to the Laboratory Manual for further instruction.

5.9 ONC-392 Dose Adjustment

If a patient has treatment-related adverse events (TRAEs), the dose may be adjusted to a lower level for subsequent cycles of treatment. Alternatively, the Investigator may wish to withhold the treatment until the initial TRAEs are resolved before having the patient restart the treatment at a reduced dose level. Dose adjustment for Arm 1 refers to **Table 12**. After the dose is adjusted to a lower level, the Investigator may re-evaluate the patient and adjust the dose to a higher level up to the original dose level in the study design. Please refer to **Section 7.9** for irAE management recommendation.

Table 12. ONC-392 Dose Adjustments

Dose Adjustment	ONC-392 in Cycles 1&2	ONC-392 in Cycles 3 to 17
Dose Level 0 (assigned dose)	10 mg/kg	6 mg/kg
Dose Level -1	6 mg/kg	3 mg/kg
Dose Level -2	3 mg/kg	1 mg/kg*

*ONC-392 will be administered by intravenous infusion over a period of approximately 30 minutes for dose level of 1 mg/kg.

5.10 Docetaxel IV Infusion and Dose Adjustment

Docetaxel IV infusion will be given over a period of approximately 60 minutes at 75 mg/m^2 dose.

All patients should pre-medicate with oral or injectable steroids according to the approved product label and/or standard practice.

For docetaxel handling and storage, please refer to the product package insert.

If a patient experiences either febrile neutropenia, neutrophils <500 cells/mm^3 for more than one week despite support with granulocyte stimulating factor, severe skin reactions or other grade 3 or 4 non-hematological toxicities according to CTCAE v5.0, treatment should be withheld until resolution of the toxicity and then resume docetaxel at a reduced dose in reference to **Table 13**. Patients who develop grade ≥3 peripheral neuropathy should have docetaxel treatment discontinued permanently.

Table 13. Dose Modification Standards for Adverse Events Related to Docetaxel

**Docetaxel Dose Reduction**	
**Dose Level**	**Docetaxel**
Initial Dose	75 mg/m^2
First reduction	55 mg/m^2
Second reduction	37.5 mg/m^2
Third reduction	Discontinue Docetaxel

All dose adjustments and reasons for dose adjustments should be documented on the eCRFs.

5.11 Local Cancer Treatment

During the study treatment period, patients may receive palliative treatment as part of the best supportive care. Patient may continue the ONC-392 or docetaxel treatment after the local therapy. There should be a 7-day rest period after local therapies before resuming ONC-392 or docetaxel treatment. All local treatment procedures, including surgical intervention or radiation therapy, during the study must be recorded in eCRF.

5.12 End of Treatment (EOT) Visit and Follow-up Period

When a patient exits from the study, the EOT visit should be conducted within 30 + 7 days when the decision to discontinue the study treatment is made. The Overall Survival (OS) is the primary objective for this Phase 3 study. All patients should enter the survival follow-up period after the end of treatment. Survival follow-up includes both the progression free survival (PFS) and the overall survival (OS). If there is new anti-cancer treatment, the treatment regimen should be recorded in eCRF.

1. Patients who discontinue treatment due to PD should enter the survival follow-up (Q16 ±3W) by chart review or telemedicine or visit. Next line anti-cancer treatment regimen should be recorded.
2. Patients who complete 17 cycles (approximately 1 year) of study treatment, their EOT visit will be within 30 days after Cycle 17 followed by PFS/survival follow-up (Q16 ±3W) by CT scans, chart review or telemedicine or visit.
3. Patients who discontinue from the treatment due to an AE (without documented PD) will have safety follow-up until the resolution of the AE to Grade 0-1 or baseline level and enter the PFS/survival follow-up period. Best effort should be made to follow the patients for PFS (by tumor imaging Q9±1W through 1 year and Q16±3W after 1 year) until disease progression or any new anti-cancer therapy starts. Patients should be followed up for survival (Q16 ±3W) by chart review or telemedicine or visit or by public sources. All patients will be followed for overall survival until death, withdrawal of consent to survival follow up or the end of the study.
4. Best effort should be made to follow the survival (Q16 ±3W) for patients who discontinue study treatment and elect hospice service. All patients will be followed for overall survival until death, withdrawal of consent to survival follow-up or the end of the study.
5. Patients who withdraw from the treatment (without documented PD) should enter the PFS/survival follow-up period. Best effort should be made to follow the patients for PFS (by tumor imaging Q9±1W through 1 year and Q16±3W after 1 year) until disease progression or any new anti-cancer therapy starts and for survival (Q16 ±3W) by chart review or telemedicine or visit. Any new line of anti-cancer treatment regimen patient receives should be recorded.
6. Patients who withdraw from the study, including the withdraw of consent to survival follow-up, will not be contacted for survival. Public sources are to be searched for vital status information for up to the maximal follow-up period for such patient. If vital status is determined as deceased, this will be documented in the EDC.

5.13 Optional Treatment after One Year

Patients who achieve and maintain disease control (i.e., CR, PR, or SD, or in post-PD treatment) through the end of the 17 cycles of treatment have the option to continue treatment with their prior assigned treatment (ONC-392 or docetaxel) upon the recommendation of investigators. The Clinical Investigator should send the recommendation and the request to Sponsor for extended optional treatment.

Patients who have optional treatment after one year should have AE assessment and concomitant medication recorded on every treatment visit. The assessments related to vital signs, physical examinations and drug administration will be required in every cycle. Routine laboratory tests listed in **Table 11** will be performed in every even numbered cycle afterward unless otherwise specified (see footnote #8 in **Table 1**). Tumor assessment should be done Q16 ± 3 weeks for PFS follow-up. Patients should enter the survival follow-up period when they discontinue the optional treatment.

5.14 Discontinuation of Investigational Product (IP)

Discontinuation of study treatment does not represent withdrawal from the trial. All patients who discontinue study treatment will continue to participate in the trial and be followed up for disease progression and survival as specified in **Table 1**, **Section 1.5**.

Patients may discontinue treatment at any time for any reason or be discontinued by Investigator or Sponsor, including the reasons listed below. All patients should enter the survival follow-up period. Patients may discontinue study treatment due to the reasons including but not limited to the following:

1. Withdrawal of consent from further treatment with IP. Best effort should be made to keep the consent of overall survival (OS) follow-up for the study primary objective and endpoint.
2. Lost to follow-up (for OS follow-up please see **Section 5.15** for detail).
3. Any severe or serious AE that prevents further study treatment.
4. Patient noncompliance that, in the opinion of the Investigator or Sponsor, warrants discontinuation of treatment (e.g., refusal to adhere to scheduled visits).
5. Disease progression and/or upon Investigator's determination that the patient is no longer benefiting from the treatment with IP. Continue treatment beyond disease progression is allowed at the discretion of Investigator.
6. Pregnancy or intent to become pregnant.

Patients who discontinued docetaxel treatment are not allowed to crossover to receive ONC-392 treatment.

5.15 Lost to Follow-up

A patient will be considered lost to follow-up if he or she repeatedly fails to return for scheduled visits and is unable to be contacted by the study site.

The following actions must be taken if a patient fails to return to the clinic for a required study visit:

1. The site must attempt to contact the patient and reschedule the missed visit as soon as possible and counsel the patient on the importance of maintaining the assigned visit schedule and ascertain whether or not the patient wishes to and/or should continue in the study.
2. Before a patient is deemed lost to follow-up, the investigator or designee must make every effort to regain contact with the patient (where possible, 3 telephone calls and, if necessary, a certified letter to the patient's last known mailing address or local equivalent methods). These contact attempts should be documented in the patient's medical record.
3. The patient is not considered lost to follow-up if the investigator regains the contact any time during the study.
4. Site personnel, or an independent third party, will attempt to collect the vital status of the patient within legal and ethical boundaries for all patients randomized, including those who did not get study intervention. Public sources may be searched for vital status information. If vital status is determined as deceased, this will be documented, and the patient will not be considered lost to follow-up. Sponsor personnel will not be involved in any attempts to collect vital status information. The patient is not considered lost to follow-up if the vital status is known.

5.16 Subject Replacement Strategy

A subject who discontinues from the trial treatment or withdraws consent after randomization in the trial will not be replaced. A subject who withdraws consent before randomization will be replaced.

5.17 Beginning and End of the Study

CCI [redacted]

[redacted]

[redacted]

[redacted]

5.18 Study Completion

A patient is considered to have completed the study if he/she meets the following criteria:

- Has completed the 17 cycles of treatment and has survival follow-up till death or the end of study.
- Has permanently discontinued the study treatment and has survival and/or PFS follow-up till death or the end of study.
- Has withdrawn consent specified for overall survival follow-up.
- Has OS event any time after randomization.

5.19 Options for Patients Enrolled Prior to Protocol Amendment v3.0

Patients with non-squamous NSCLC who are active in the study may continue to receive assigned treatment if the treating physician considers there is potential benefit. These patients should be given the option to withdraw from the study. The enrolled squamous NSCLC patients should continue to receive assigned treatment.

6 STUDY ASSESSMENTS

An electronic data capture (EDC) system will be used for data collection and query handling. It is the Investigator's responsibility to ensure that data are recorded on the eCRFs as specified in the study protocol and in accordance with the eCRF Completion Guidelines.

The Investigator is also responsible to ensure the accuracy, completeness, and timeliness of the data recorded and of the provision of answers to data queries according to the Clinical Study Agreement (CSA). Protocol deviations that might significantly affect the completeness, accuracy, and/or reliability of study data or that might significantly affect a subject's rights, safety, or well-being are important protocol deviations and should be reported to the sponsor expeditiously. The Investigator will sign the completed eCRFs. A copy of the completed eCRFs will be archived at the study site.

6.1 Efficacy Measurements

The following evaluations will be performed throughout the course of the study:

1. Tumor response assessments by clinical assessment and tumor imaging by CT/CAP or MRI. The investigator will provide the clinical evaluation.
2. Record of Overall Survival events.

6.2 List of Safety Measurements

Toxicity (AEs) will be graded and recorded according to NCI CTCAE v5.0 (http://ctep.cancer.gov).

The safety evaluations will be performed at baseline and during the study as indicated in **Table 1** and as medically indicated in the opinion of the treating physician(s):

1. Vital signs
2. Physical examination
3. ECOG status
4. Medical history
5. Evaluation of AEs
6. Laboratory tests: complete blood count (CBC), serum chemistry, thyroid function tests, and other tests that are clinically indicated.

Any clinically significant abnormal laboratory test should be repeated as soon as possible by either local site laboratory or by collecting samples and shipping to the Sponsor designated central laboratory by following the instructions in the Laboratory Manual (preferably within 24 to 48 hours). The initial and repeat results should be recorded on the eCRF. Situations in which laboratory safety results should be reported as AEs are described in **Section 7.**

All patients with Grade 3 or 4 laboratory values at the time of completion or discontinuation from study treatment must have further tests performed until the laboratory values have returned to Grade < 2 unless these values are not likely to improve because of the underlying disease. The safety parameters will be evaluated according to the Medical Monitoring plan and the SAE listing will be reviewed by the DMC Chairperson monthly to oversee the safety of the participants.

6.3 Efficacy Assessments

The overall survival will be the primary efficacy endpoint and be monitored for the duration of the study. After treatment completion or permanent discontinuation, overall survival will continue to be followed up every 16 weeks (± 3 weeks) by phone, email or clinical visits.

The secondary efficacy endpoints of ORR and PFS will use imaging-based disease assessment via CT or MRI within 28 days prior to C1D1 (preferably closer to C1D1) and every 9 ± 1 weeks for the first year and every 16 ± 3 weeks after first year until confirmed PD. After the PR, CR or PD is observed, a confirmatory scan should be performed within 4-6 weeks after the initial evaluation. The subsequent assessments can be adjusted to every 9±1 week after the unscheduled confirmation scan. If a patient discontinues study treatment for reasons other than disease progression, the patient should be followed for PFS with imaging every 9±1 week until PD or any new anti-cancer therapy starts. The same imaging technique should be used in an individual patient throughout the study. Images for areas of disease outside of the usual chest, abdomen or pelvis for CT scans or brain metastasis for MRI should be obtained based on the clinical indication.

RECIST 1.1 criteria will be used to assess the tumor response to treatment and derive ORR, DoR, DCR, and PFS. The RECIST 1.1 definitions for measurable, non-measurable, target, and non-target lesions, and the objective tumor response criteria (CR, PR, SD, or PD) are presented in Eisenhauer et al., 2009 [58]. All images related to the tumor assessment should be uploaded to the specified portal following the Image Manual for study image collection.

Categorization of objective tumor response assessment will be based on the RECIST 1.1 criteria of response: CR, PR, SD, and PD. Target lesion progression will be calculated in comparison to when the tumor burden is at a minimum (i.e., smallest sum of diameters previously recorded on study). In the absence of progression, tumor response (CR or PR) and SD will be calculated in comparison to the baseline tumor measurements obtained before starting treatment.

6.4 Pharmacokinetics

6.4.1 Collection of PK/ADA/Biomarker Samples and ctDNA Samples

Blood samples for determination of PK/ADA/Biomarker will be collected pre-dose at Cycles 1, 2, 4, 8, 12, and 16 (see **Section 1.5**). ctDNA samples will be collected pre-dose at Cycle 1. Details on sample processing, handling, shipment, and storage are provided in the Laboratory Manual.

6.4.2 Storage and Destruction of PK/ADA/Biomarker Samples

CCI [redacted]

[redacted]

[redacted]

[redacted]

6.4.3 Chain of Custody of Biological Samples

CCI [redacted]

[redacted]

CCI [redacted]

[redacted]

[redacted]

6.4.4 Withdrawal of Informed Consent for Donated Biological Samples

If a patient withdraws consent for the use of donated biological samples, the samples will be disposed of or destroyed, and the action will be documented. If samples have already been analyzed, OncoC4 is not obliged to destroy the results of this research.

6.5 Health-related Quality-of-life Questionnaires

Participants randomized under Protocol Version 2.0 and later will complete the HRQoL questionnaires in their entirety at specified timepoints during the study (see schedule of assessments, **Table 1**). The questionnaires, translated into the local language as appropriate, will be completed via an application using a mobile phone provided centrally or the participant's own personal device. Participants may complete these questionnaires at the study site or at home, without assistance or interpretation from others.

If questionnaires are completed at the study site, they will be completed prior to the administration of study treatment, clinical assessments, study procedures, or any information on disease status. This ensures instrument validity and compliance with health authority data standards. For participants completing questionnaires at home, they should be completed preferably either on the same day as the intended site visit before the visit or on the evening before the site visit (no earlier than 24h before the visit). Data will be electronically submitted, and completeness and consistency will be regularly monitored.

The participant will complete questionnaires preferably according to the prespecified order defined hereafter:

1. EORTC QLQ-C30
2. EORTC QLQ-LC29
3. NSCLC-SAQ
4. EQ-5D-5L

<u>EORTC QLQ-C30</u>

EORTC QLQ-C30 is the most widely used cancer-specific, HRQoL instrument containing a total of 30 items and measures five functional scales (physical, role, emotional, cognitive, and social), three symptom scales (fatigue, nausea/vomiting, and pain), six single items (dyspnea, insomnia, appetite loss, constipation, diarrhea, and financial difficulties), and a global health status/QoL scale [59]. All the scales and single-item measures range in score from 0 to 100 with a high scale score representing a higher response level (e.g., high score for functional scale is high/healthy level of functioning; high score for global health status/QoL is high QoL: high score for symptom scale/item is high symptomatology/problems). The EORTC QLQ-C30 takes approximately 10 minutes to complete and has a recall period of the previous week.

EORTC QLQ-LC29

The EORTC QLQ-LC29 is a disease-specific supplementary HRQoL questionnaire module to be employed in conjunction with the QLQ-C30 [60]. It comprises of 29 items and measures five multi-item scales (coughing, shortness of breath, side-effects, tumor progression/existential issues, surgery-related symptoms) and five single items (coughing up blood, pain in chest, arm/shoulder and other parts of the body, and weight loss). All the scales and single-item measures range in score from 0 to 100 with a high score for the scales and single items representing a high level of symptomatology or problems. The EORTC QLQ-LC29 takes approximately 10 minutes to complete and has a recall period of the previous week.

NSCLC-SAQ

The NSCLC-SAQ is a 7-item patient-reported outcome measure for use in adults to assess symptoms of advanced NSCLC [61]. It contains five domains and accompanying items that were identified as symptoms of NSCLC: cough (1 item), pain (2), dyspnea (1), fatigue (2), and appetite (1). The (total) lowest score possible is 0, and the highest (total) score possible is 20. Higher scores indicate more severe symptoms. The NSCLC-SAQ takes approximately 3-5 minutes to complete and has a recall period of the previous week.

EQ-5D-5L

The EQ-5D-5L is a self-administered questionnaire and consists of the EQ-5D descriptive system and the EQ-5D Visual Analog Scale (VAS) [62]. The descriptive system comprises of five dimensions (mobility, self-care, usual activities, pain/discomfort, and anxiety/depression), each with five levels of severity (no problems, slight problems, moderate problems, severe problems, and extreme problems). The EQ-5D VAS records the self-reported health on vertical VAS numbered from 0 to 100. On the VAS, 100 means “the best health you can imagine” and 0 means “the worst health you can imagine”. The EQ-5D-5L takes only a few minutes to complete. The self-reported health status captured by EQ-5D-5L relates to the respondent’s situation at the time of completion.

All the HRQoL questionnaires should be given to randomized patients prior to initiation of study procedures and AE evaluation.

7 ADVERSE EVENTS

The clinical PIs are responsible for ensuring that all staff involved in the study are familiar with the content of this section.

7.1 Definition of Adverse Events (AEs)

An AE is the development of an undesirable medical condition or the deterioration of a pre-existing medical condition following or during exposure to a pharmaceutical product, whether or not considered causally related to the product.

An undesirable medical condition can be signs (e.g., enlarged liver, skin rash), symptoms (e.g., nausea, headache, chest pain), or the abnormal results of an investigation (e.g., laboratory findings).

The term AE is used to include both serious and nonserious AEs.

7.2 Definition of Serious Adverse Events (SAEs)

A serious adverse event (SAE) is an AE that meets any of the following outcome criteria and may require expedited reporting by the Sponsor to regulatory authorities:

- Results in death
- Is immediately life-threatening
- Requires inpatient hospitalization ⩾ 24 hours or prolongation of existing hospitalization.
- Results in persistent or significant disability/incapacity or substantial disruption of the ability to conduct normal life functions.
- Is a congenital abnormality or birth defect.
- Is an important medical event that may jeopardize the patient or may require medical intervention to prevent one of the outcomes listed above.

7.3 Grading Adverse Events (Serious and Non-serious)

Assessment of severity for all AEs (serious and non-serious) will be made according to the NCI CTCAE v5.0. Any adverse event that changes NCI CTCAE grade over the course of a given episode will have each change of grade recorded on the AE CRFs/worksheets.

- **Grade 1**: Mild; asymptomatic or mild symptoms; clinical or diagnostic observations only; intervention not indicated.
- **Grade 2**: Moderate; minimal, local, or noninvasive intervention indicated; limiting age-appropriate instrumental activities of daily living (ADLs).
- **Grade 3**: Severe or medically significant but not immediately life-threatening; hospitalization or prolongation of hospitalization indicated; disabling; limiting self-care ADLs.
- **Grade 4**: Life-threatening consequences; urgent intervention indicated.
- **Grade 5**: Death related to AE.

7.4 Collecting and Recording Adverse Events

AEs occurring from the start of study treatment and up to 30 days after the last dose will be documented, recorded, and reported. All SAEs and immune-related AEs (irAEs) will be collected from the start of

study treatment to 90 days after the last dose of the treatment or to the date that the patient initiates new anti-cancer therapy, whichever comes first.

Any SAE considered related to study treatment that occurs from the initiation of study treatment through the end of the specified safety follow-up period, as described above, or at any time thereafter, must be reported to the Sponsor immediately. AEs occurring outside the specified safety follow-up period, other than SAEs considered related to study treatment, are not required to be collected.

Medical occurrences that begin before the start of study intervention but after obtaining informed consent will be recorded as medical History/Current Medical Conditions, not as AEs.

The Investigator will evaluate AEs with respect to **Severity** (intensity or grade) and **Causality** (relationship to study agents and relationship to study procedure). The Investigator will record nonserious and serious AEs in EDC system. The Investigator will report SAEs to the Sponsor on the SAE Report Form as described in **Section 7.5**.

Laboratory abnormalities that are clinically significant per the NCI CTCAE v5.0 will be recorded as AEs. All other laboratory abnormalities without clinical significance or without worsening from the baseline will not be recorded as AEs. If a diagnosis is clinically evident, the diagnosis, rather than the individual signs and symptoms or laboratory abnormalities will be recorded as the AE.

Events will be summarized on the basis of the date of onset for the event. A TEAE will be defined as any new or worsening AE that begins on or after the date of treatment until study completion. Summaries (number and percentage of patients) of TEAEs will be provided at study completion.

At each visit following the study schedules in **Section 1.5**, information regarding AEs will be elicited by appropriate questioning and examinations and will be immediately documented in the patient's medical record. Medical records will be reviewed in a timely manner by the research team. The onset date, the end date, the severity of each reportable event, and the Investigator's judgement of the AEs relationship to the study drug should be recorded. Patients will be followed up until the adverse event has been resolved or returned to baseline.

The AE reporting variables are:

- AE (verbatim)
- The date when the AE started and stopped
- The maximum NCI CTCAE grade reported
- Changes in NCI CTCAE grade
- Whether the AE is serious or not
- Investigator causality rating for relatedness to study drug (yes or no)
- Action taken with regard to study drug
- Administration of treatment for the AE
- Whether the AE caused the patient's withdrawal from the study (yes or no)
- Outcome

7.5 Serious Adverse Event Reporting

All SAEs and immune-mediated AEs will be collected from the time of treatment to 90 days after the last dose of the treatment or to the date that the patient initiates new anti-cancer therapy, whichever comes first.

The Investigator should report to the Sponsor/IQVIA Safety any SAE defined in **Section 7.2** immediately and within 24 hours when he/she becomes aware of it, whether or not considered study drug related, including those listed in the protocol or Investigator's Brochure and must include an assessment of whether there is a reasonable possibility that the study intervention caused the event. The initial SAE report should be submitted to the Sponsor/IQVIA Safety Management Team by email within 24 hours when the study site becomes aware of the event. Any updated SAE information will be submitted to the Sponsor/IQVIA Safety Management Team when available.

The Sponsor has a legal responsibility to notify both the local regulatory authority and other regulatory agencies about the safety of a study intervention under clinical investigation. The Sponsor will comply with country-specific regulatory requirements relating to safety reporting to the regulatory authority, EU Eudravigilance Database (as applicable), IRBs/IECs (or RA/EC), and investigators.

All SAEs will be followed until satisfactory resolution or until the Investigator deems the event to be chronic or the patient is stable. In most of the cases, for hospitalization related SAEs, the discharge date from hospital could be used as the SAE end date. Other supporting documentation of the event may be requested by the study Sponsor/IQVIA Safety and should be provided as soon as possible.

CCI [redacted]

All treatment emergent SAEs should be recorded using EDC and source documents. The SAE report should be submitted electronically to:

[redacted]

The SAE recording variables are the following, as applicable:

- Date the AE met criteria for SAE
- Date the Investigator became aware of the SAE
- Seriousness criteria fulfilled
- Date of hospitalization
- Date of discharge
- Probable cause of death
- Date of death
- Whether an autopsy was performed
- Causality assessment in relation to study procedure(s)
- Causality assessment in relation to other medication
- Description of the AE
- Any other variable required for AE reporting (see **Section 7.4**)

7.6 Assessing Causality

Investigators are required to assess whether there is a reasonable possibility that the study drug (ONC-392 or docetaxel) caused or contributed to an AE. The following general guidance may be used.

- Related: If the temporal relationship of the clinical event to study drug administration makes a causal relationship likely and other drugs, therapeutic interventions or underlying conditions do not provide a sufficient explanation for the observed event.
- Not related: If the temporal relationship of the clinical event to study drug administration makes a causal relationship unlikely, or other drugs, therapeutic interventions or underlying conditions provide a sufficient explanation for the observed event.

7.7 Disease Progression

Disease progression can be considered as a worsening of a patient's condition attributable to the disease for which the investigational product is being studied. It may be an increase in the severity of the disease under study and/or increases in the symptoms of the disease. The development of new, or progression of, existing metastasis to the primary cancer under study should be considered as disease progression and not an AE. Events that are unequivocally due to disease progression should not be reported as an AE during the study.

7.8 Deaths

Overall survival is the primary endpoint of the study. All deaths after randomization must be recorded in EDC. All deaths that occur during the study, or within the protocol-defined follow-up period after the administration of the last dose of study treatment, must be reported as follows:

- Death clearly resulting from disease progression should be reported to the Sponsor at the next monitoring visit and should be documented in the eCRF. It should not be reported as an SAE.
- Where death is not due (or not clearly due) to progression of the disease under study, the AE causing the death must be reported to the Sponsor as an SAE within 24 hours. The report should contain a comment regarding the co-involvement of PD, if appropriate, and should assign main and contributory causes of death.
- Deaths with an unknown cause should always be reported as an SAE. A post-mortem may be helpful in the assessment of the cause of death, and if performed, a copy of the post-mortem results should be forwarded to the Sponsor/CRO.

7.9 Immune-related Adverse Event (irAE) Management Plan

7.9.1 General Management of irAEs

Immune-related adverse events (irAEs) are anticipated adverse events in the study as ONC-392 being a checkpoint inhibitor. All irAEs will be evaluated and recorded from the start of the study intervention to 90 days after the last dose of the treatment or to the date that the patient initiates new anti-cancer therapy, whichever comes first.

The irAEs may occur shortly after the first treatment or several months after the last dose of ONC-392 treatment and may affect more than one body system simultaneously. Therefore, early recognition and initiation of treatment is critical to reduce complications. Based on existing clinical study data, most irAEs were reversible and could be managed with interruptions of ONC-392 treatment, administration of corticosteroids and/or other supportive care. For suspected irAEs, ensure timely and adequate evaluation to confirm etiology or exclude other causes. Additional procedures or tests such as bronchoscopy, endoscopy, skin biopsy may be included as part of the evaluation. Dose modification and toxicity

management guidelines for irAEs associated with ONC-392 are provided in **Table 14**, **Table 15** and **Table 16**.

Holding Study Interventions:

If the AE is considered immune-related, ONC-392 should be held according to recommended dose modifications (**Table 14**, **Table 15** and **Table 16**).

Restarting Study Interventions:

If the toxicity does not resolve or the criteria for resuming treatment are not met, the participant must be discontinued from all study interventions.

If the toxicities are resolved, ONC-392 treatment may be restarted at the discretion of the investigator. The dose reduction is allowed (see **Section 5.9**).

7.9.2 Management Recommendations of the Most Common ONC-392-related irAEs

During ONC-392 treatment, when irAEs are suspected, infectious cause must be ruled out before initiation of any treatment for the irAE. The treatments should follow the NCCN and ASCO guidelines for most irAE types. Based on clinical experience of ONC-392 and recent literature, treatments for study drug-related colitis and hepatitis should follow the modified guidelines.

Table 14. Recommended Management Plan for ONC-392-related Diarrhea/Colitis

Adverse Events (per CTCAE v5)	Grade 1	Grade 2	Grade 3	Grade 4
**Diarrhea/ Colitis**	Diarrhea: < 4 stools/day over baseline<br>Colitis: asymptomatic	Diarrhea: 4-6 stools per day over baseline; IV fluids indicated < 24 hrs; Limiting instrumental ADL<br>Colitis: abdominal pain; blood in stool	Diarrhea: ≥ 7 stools per day over baseline; IV fluids ≥ 24 hrs; limiting self-care ADL<br>Colitis: severe abdominal pain; peritoneal signs	Life-threatening, perforation
	• Continue ONC-392 therapy per protocol<br>• Initiate symptomatic treatment<br>• Close monitoring for worsening symptoms<br>• Educate patient to report worsening immediately<br>If worsens:<br>• Treat as Grade 2 or Grade 3-4	• Delay ONC-392 therapy per protocol<br>• Initiate symptomatic treatment<br>• 1-2 mg/kg/day methylprednisolone or oral equivalent<br>• When symptoms improve to Grade 1, taper steroids over at least 1 month, consider prophylactic antibiotics for opportunistic infections, and resume ONC-392 therapy per protocol<br>If worsens or persists > 2 – 3 days with oral steroids:<br>• Treat as Grade 3-4	• Withhold ONC-392 therapy per protocol<br>• 1-2 mg/kg/day methylprednisolone IV or IV equivalent<br>• Add prophylactic antibiotics for opportunistic infections<br>• Consider lower endoscopy<br>If persists > 3-5 days, or recurs after improvement [63]:<br>• Add 300 mg/dose of vedolizumab at 0, 2, 6 and 14 weeks.<br>• Consider testing CMV for steroid refractory colitis<br>If improves:<br>• Continue steroids until grade 1, then taper over at least 1 month<br>• Add one dose of 300 mg vedolizumab within one week of resuming ONC-392 treatment per protocol	

Table 15. Recommended Management Plan for ONC-392-related Liver Function Abnormalities and Hepatitis

While standard serum liver enzyme panels should be performed, treatment guideline is based on serum enzyme tests of ALT as AST elevation is less specific to hepatocyte injuries.

**Adverse Events (per CTCAE v5)**	**Grade 1**	**Grade 2**	**Grade 3**	**Grade 4**
**Liver Function Test**	ALT > ULN to 3X ULN and/or<br>T.bili > ULN to 1.5X ULN	ALT > 3X to 5X ULN and/or<br>T.bili > 1.5X to 3X ULN	ALT > 5X ULN to 20X ULN and/or<br>T.bili > 3X ULN to 10X ULN	ALT > 20X ULN and/or<br>T.bili > 10X ULN
	• Continue ONC-392 therapy per protocol<br>• Consider increasing frequency of monitoring<br>• Continue LFT monitoring per protocol<br><br><u>If worsens</u>:<br>• Treat as Grade 2 or Grade 3-4	• Withhold ONC-392<br>• Increase frequency of monitoring to every 3 days<br><u>If returns to baseline</u>:<br>• Resume routine monitoring, resume ONC-392 therapy per protocol<br><u>If elevations persist > 5-7 days or worsen</u>:<br>• 0.5-1 mg/kg/day methylprednisolone or oral equivalent<br>• When ALT return to <3X ULN and bili <1.5X ULN, taper steroids over at least 1 month, consider prophylactic antibiotics for opportunistic infections, and resume ONC-392 therapy per protocol	• Withhold ONC-392<br>• Increase frequency of monitoring to every 1-2 days<br>• 1-2 mg/kg/day methylprednisolone IV or IV equivalent*<br>• Add prophylactic antibiotics for opportunistic infections<br>• Consult gastroenterologist or hepatologist<br><br><u>If ALT return to <5X ULN and bili <3X ULN</u>:<br>• Taper steroids over at least 1 month, and follow the Grade 2 management.<br><br><u>If does not improve in >3-5 days, worsens, or rebounds</u>:<br>• Add mycophenolate mofetil (MMF) 1 g BID<br>• If no response within an additional 3-5 days, perform liver biopsy and consider tacrolimus or other immunosuppressants per local guidelines<br><br>Note: Avoid infliximab due to potential risk of liver failure	

Patients on IV steroids may be switched to an equivalent dose of oral corticosteroids (e.g., Prednisone) at start of tapering or earlier, after sustained clinical improvement is observed. Lower bioavailability of oral corticosteroids should be taken into account when switching to the equivalent dose of oral corticosteroids.

*The recommended starting dose for ALT > 20X ULN or bilirubin > 10X ULN is 2 mg/kg/day methylprednisolone IV.

7.9.3 Other irAEs

In addition to immune-mediated colitis and immune-mediated hepatitis, other irAEs may occur after initiation of ONC-392 treatment. Early recognition, diagnosis, and prompt treatment of these events are key to preventing complications or fatal outcomes.

When patients experience a new onset of headache, fatigue, weakness, or hypotension, there should be a suspicion of immune-mediated endocrinopathy in addition to neuropathy and disorders of other systems. Workup should be done to rule out hypothyroidism, adrenal insufficiency, or hypophysitis, including thyroid function test and serum ACTH and cortisol test as well as consultation with an endocrinologist. Hormone replacement therapy can effectively manage these conditions, allowing patients to continue their study drug treatment. Table 16 provides detailed recommendations on management of these events as well as other irAEs. ONC-392 treatment may re-start when endocrinopathy ≤ Grade 2 and the symptoms are controlled by hormone replacement therapy. The steroid replacement daily dose is allowed to be higher than 10 mg/day prednisone or equivalent for re-start ONC-392 treatment.

Table 16. Recommended Dose Modifications for irAEs

General instructions:

1. Severe and life-threatening irAEs should be treated with IV corticosteroids followed by oral steroids. Other immunosuppressive treatment should begin if the irAEs are not controlled by corticosteroids.
2. The corticosteroid taper should begin when the irAE is ≤ Grade 2 and continue at least 4 weeks.
3. ONC-392 may resume after the irAE decreased to ≤ Grade 1 after corticosteroid taper. Dose reduction should be considered. If control achieved or ≤ Grade 2 for endocrinopathy, ONC-392 may be resumed.

irAEs	Toxicity grade (CTCAE V5.0)	Action with ONC-392	Corticosteroid and/or other therapies	Monitoring and follow-up
Myocarditis	Asymptomatic cardiac enzyme elevation or Grade 2 or 3	Withhold	Based on severity of AE administer corticosteroids	Ensure adequate evaluation to confirm etiology and/or exclude other causes. Cardiologist consultation.
	Grade 4	Permanently discontinue		
Hyperthyroidism	Grade 2 or 3	Continue	Treat with nonselective beta-blockers (eg, propranolol) or thionamides as appropriate	Monitor for signs and symptoms of thyroid disorders. Endocrinologist consultation.
	Grade 4	Withhold or permanently discontinue[a]		
Hypothyroidism	Grade 2, 3 or 4	Continue	Initiate thyroid replacement hormones (eg, levothyroxine or liothyronine) per standard of care	Monitor for signs and symptoms of thyroid disorders
Adrenal Insufficiency[b]	Grade 2, 3 or 4	Continue	Initiate hydrocortisone replacement per standard of care	Monitor for signs and symptoms of adrenal insufficiency. Test serum ACTH and cortisol levels for new onset of headache,

				fatigue, weakness, or hypotension. Endocrinologist consultation.
Pneumonitis	Grade 2	Withhold	Administer corticosteroids (initial dose of 1 to 2 mg/kg prednisone or equivalent) followed by taper<br>Add prophylactic antibiotics for opportunistic infections	Monitor participants for signs and symptoms of pneumonitis<br>Evaluate participants with suspected pneumonitis with radiographic imaging and initiate corticosteroid treatment
	Grade 3 or 4	Permanently discontinue		
Neurological Toxicities	Grade 2 or 3	Withhold	Based on severity of AE administer corticosteroids, IVIG treatment.	Ensure adequate evaluation to confirm etiology and/or exclude other causes
	Grade 4	Permanently discontinue		
Nephritis: grading according to increased creatinine or acute kidney injury, not corrected by fluid replacement or hydration	Grade 2 or 3	Withhold	Administer corticosteroids (prednisone 1 to 2 mg/kg or equivalent) followed by taper	Monitor changes of renal function
	Grade 4	Permanently discontinue		
Exfoliative Dermatologic Conditions	Suspected SJS, TEN, or DRESS	Withhold	Based on severity of AE administer corticosteroids	Ensure adequate evaluation to confirm etiology or exclude other causes
	Confirmed SJS, TEN, or DRESS	Permanently discontinue		
All other irAEs	Grade 2 or 3	Withhold	Based on severity of AE administer corticosteroids	Ensure adequate evaluation to confirm etiology or exclude other causes
	Grade 4	Case by case evaluation		

AE(s)=adverse event(s); ALT= alanine aminotransferase; AST=aspartate aminotransferase; CTCAE=Common Terminology Criteria for Adverse Events; DRESS=Drug Rash with Eosinophilia and Systemic Symptom; GI=gastrointestinal; IO=immuno-oncology; ir=immune-related; IV=intravenous; SJS=Stevens-Johnson Syndrome; TEN=Toxic Epidermal Necrolysis; ULN=upper limit of normal.

Note: Non-irAE will be managed as appropriate, following clinical practice recommendations.

[a] The decision to withhold or permanently discontinue ONC-392 is at the discretion of the investigator or treating physician. If control achieved or ≤ Grade 2 for endocrinopathy, ONC-392 may be resumed.

[b] Patient with adrenal insufficiency should be treated with adequate dose of hormone replacement. The study treatment may resume when the symptoms resolved. There is no restriction on the amount of steroid for hormone replacement.

7.10 Recommended Prophylactic and Acute Management of Infusion Reactions

Please refer to product label for patients receiving docetaxel.

Infusion-related reactions (IRRs) have been reported with administration of ONC-392 monotherapy with severity of grade 1 or grade 2. Most IRRs occurred at the ONC-392 dose level of 10 mg/kg with severity of grade 1 or grade 2. The onset of infusion reaction was mostly within 30-40 minutes after the infusion. The infusion reaction required treatment and resolved in about 30 minutes.

Prophylactic pre-medication should be given prior to the administration of ONC-392 in first treatment (C1D1), recommended to be approximately 30-60 minutes before ONC-392 infusion depending on the type of pre-medication and route of administration of the agent and/or as per local standards of care. The recommended pre-medications for ONC-392 (clinical investigators may make adjustment to prescribe one or more from the list according to local institutional guideline) are:

- Antipyretics (e.g., acetaminophen 325 to 1,000 mg PO or NSAIDs PO)
- Antihistamine (e.g., diphenhydramine 50 mg PO)
- H2 blocker (e.g., famotidine 20 mg PO)

Post-infusion management:

- Vigilant and active surveillance
- Continued IV fluids
- Treatment medication as indicated

Infusion reactions should be graded according to NCI CTCAE v5.0 infusion-related reaction. These reactions may manifest with signs and symptoms that may include, but are not limited to, fever, chills, rigor, headache, rash, pruritus, arthralgias, hypo- or hypertension, bronchospasm or other symptoms. Treatment recommendations are provided below in **Table 17**.

Table 17. Recommended Dose Modification and Toxicity Management Guidelines on ONC-392 Associated Infusion Reactions

NCI CTCAE Grade	Treatment	Premedication at Subsequent Dosing
**Grade 1**<br>Mild reaction; infusion interruption not indicated; intervention not indicated	Increase monitoring of vital signs as medically indicated until the participant is deemed medically stable in the opinion of the investigator.	None
**Grade 2**<br>Requires therapy or infusion interruption but responds promptly to symptomatic treatment (e.g., antihistamines, NSAIDs, narcotics, IV fluids); prophylactic medications indicated for ≤24 hrs.	• **Stop Infusion.**<br>• Additional appropriate medical therapy may include but is not limited to:<br>• IV fluids<br>• Antihistamines<br>• NSAIDs<br>• Acetaminophen<br>• Narcotics<br>• Increase monitoring of vital signs as medically indicated until the participant is deemed medically stable in the opinion of the investigator.<br>• If symptoms resolve within 1 hour of stopping drug infusion, the infusion may be restarted at 50% of the	Participant may be premedicated approximately 30-60 minutes prior to infusion of study intervention with:<br>Diphenhydramine 50 mg po (or equivalent dose of antihistamine).<br>Acetaminophen 500-1000 mg po (or equivalent dose of analgesic).

	original infusion rate (e.g. from 100 mL/hr. to 50 mL/hr.). Otherwise dosing will be held until symptoms resolve and the participant should be premedicated for the next scheduled dose.	
**Grades 3 or 4**<br>**Grade 3:**<br>Prolonged (i.e., not rapidly responsive to symptomatic medication and/or brief interruption of infusion); recurrence of symptoms following initial improvement; hospitalization indicated for other clinical sequelae (e.g., renal impairment, pulmonary infiltrates)<br>**Grade 4:**<br>Life-threatening; pressor or ventilator support indicated	• **Stop Infusion.**<br>• Additional appropriate medical therapy may include but is not limited to:<br>• Epinephrine**<br>• IV fluids<br>• Antihistamines<br>• NSAIDs<br>• Acetaminophen<br>• Narcotics<br>• Oxygen<br>• Pressors<br>• Corticosteroids<br>• Increase monitoring of vital signs as medically indicated until the participant is deemed medically stable in the opinion of the investigator.<br>• Hospitalization may be indicated.<br>**In cases of anaphylaxis, epinephrine should be used immediately.<br>Participant is permanently discontinued from further study drug treatment.	Grade 3 that is resolved in less than 6 hours may have subsequent dosing with pre-medications.<br>**Grade 4: no subsequent dosing.**

8 STATISTICAL CONSIDERATIONS

8.1 Sample Size Rationale

[redacted]

8.2 Statistical Analyses

[redacted]

The procedure to account for missing, unused and spurious data will be detailed in the SAP. Any deviation from the SAP will be recorded and justified in the CSR. This section is a summary of the planned statistical analyses. All descriptions in this section are for the ONC-392 6 mg/kg with 2 loading doses of 10 mg/kg Q3W arm and docetaxel arm only. Analyses for the ONC-392 3 mg/kg arm will be described in the SAP.

8.2.1 General Considerations

The analyses proposed cover the analyses for efficacy and safety based on the data cut-off date for the primary analysis. Statistical analyses will be performed using cleaned eCRF data as well as data collected by the external vendor. All data will be included up to a cut-off date which is determined by the requested number of events.

Due to data cleaning activities, the final number of events might deviate from the planned number. In that case, the actual alpha spent and p-value boundaries at each analysis will be calculated based on the exact number of events at the time of analysis and required number of events for the final analysis. All remaining alpha will be spent at the final analysis.

For OS analysis, a survival sweep will be performed, and any data collected after the cut-off date (but before the database lock) will be used to determine if a participant was alive at cut-off date.

8.2.2 Definition of Baseline

For efficacy endpoints, the Baseline is defined as the last non-missing measurement before randomization. For safety endpoints, the Baseline is defined as the last non-missing measurement before the first dose of study drug.

8.2.3 Analysis Sets

[redacted]

[redacted]

[redacted]

[redacted]

[redacted]

[redacted]

8.2.4 Primary Efficacy Analyses

[redacted]

Results will be presented by treatment arm. The Kaplan-Meier method will be used to provide estimates of the OS curves, including the median, 25th and 75th percentiles and their corresponding 95% CIs. The number and percentage of patients with an OS event and those who are censored will be presented along with the reasons for censoring. The Kaplan-Meier curves will also be plotted. [redacted]

The OS rate at 6 months (OS6), 12 months (OS12), 18 months (OS18) and 24 months (OS24) will be calculated.

8.2.5 [redacted]

[redacted]

[redacted]

[redacted]

[redacted]

8.2.6 Secondary Efficacy Analysis

[redacted] Other efficacy endpoints will be considered exploratory and will be described in the SAP.

8.2.6.1 Analysis of ORR

The confirmed objective response rate (ORR) is defined as the proportion of subjects having reached a confirmed CR or PR as assessed by investigators according to RECIST 1.1.

[redacted] The ORR by treatment arm will be calculated along with the two-sided 95% CI using the Clopper -Pearson method (exact CI for a binomial proportion as computed by default by the FREQ procedure using the EXACT option).

8.2.6.2 Analysis of PFS

PFS is defined as the time from the date of randomization till the date of the first documentation of PD or death by any cause (whichever occurs first). The tumor response will be determined according to RECIST 1.1 as assessed by investigators.

The analysis method for PFS will be the same as that for OS, as specified in **Section 8.2.4**.

All efforts should be made to keep following patients for disease progression. The censoring rule for PFS includes: for subjects who have no disease progression or alive at the time of analysis, the date of the last adequate tumor assessment will be taken as the censored date; for subjects who started new anti-cancer therapy prior to the disease progression or death, the date of the last tumor assessment on or before initiation of new anti-cancer therapy will be taken as the censored date; for subjects who have progression or death immediately after more than one missed visit, the date of the last tumor assessment before the missed visit will be taken as the censored date.

8.2.7 Exploratory Efficacy Analyses and Subgroup Analyses

Additional exploratory efficacy analyses and subgroup analyses will be described in the SAP.

8.3 Safety Analyses

All safety analyses will be made on the Safety Population. All safety analyses will be descriptive; no formal statistical testing will be performed and further described in the SAP.

8.3.1 Adverse Event Analysis

AEs will be coded using the latest version of MedDRA. If a patient has multiple events with the same PT occurring in different time, the highest grade of AE will be analyzed in the table summarizing incidence at the subject level and all events will be included in the analysis summarizing event rate at the event level.

The incidence and event rate of TEAEs, TRAEs, irAEs, AEs leading to study treatment discontinuation and SAEs will be summarized overall, by treatment arm, by MedDRA system organ class (SOC) and preferred term (PT), by NCI CTCAE v5.0 grade, and by relationship to study treatment administration. Additionally, data presentations of the rate of AEs per person-years at risk may be produced.

Treatment-emergent adverse events (TEAEs) are defined as below:

- AEs that started or worsened after the first dose date and up to 30 days after the last dose, or initiation of new anti-cancer therapy, whichever is earlier.
- SAEs and irAEs that started or worsened after the first dose date and up to 90 days after last dose, or initiation of new anti-cancer therapy, whichever is earlier.
- All new or worsening AEs related to treatment, with the onset date on or after the first dose date.

8.3.2 Other Safety Analyses

Other safety analyses are described in detail in the SAP for clinical laboratory tests, vital signs, ECGs, and ECOG performance status, etc. Exposure to study treatment will be summarized.

8.4 Exploratory Analyses

8.4.1 Pharmacokinetic Analysis

The actual sampling times will be used in the PK calculations. PK concentration data and summary statistics will be tabulated. Individual and mean blood concentration-time profiles will be generated. The following PK parameters will be determined after the first and steady-state doses: peak and trough concentration (as data allow).

8.4.2 Population Pharmacokinetics and Exposure-Response/Safety Analyses

A population PK analysis will be performed using a nonlinear mixed-effects modelling approach. The impact of physiologically relevant patient characteristics (covariates) and disease on PK will be evaluated. The relationship between the ONC-392 exposure and the effect on safety and efficacy endpoints will be evaluated. The results of such an analysis will be reported in a separate report. The PK, PD, demographic, safety, and efficacy data collected in this study may also be combined with similar data from other studies and explored using population PK and/or PK-PD methods.

8.4.3 Immunogenicity Analysis

Immunogenicity results will be listed by patient and a summary will be provided of the number and percentage of patients who develop detectable anti-ONC-392 antibodies. The immunogenicity titer and neutralizing ADA data will be listed for samples confirmed positive for the presence of anti-ONC-392 antibodies. The effect of immunogenicity on PK, PD, efficacy, and safety will be evaluated, if the data allow.

8.4.4 Health-related Quality-of-life Questionnaires

The analyses for the HRQoL endpoints (EORTC QLQ-C30, EORTC QLQ-LC29, NSCLC-SAQ, EQ-5D-5L) will be further detailed in the SAP.

8.4.5 Other Exploratory Analyses

All other exploratory analyses, including characterization of regulatory and effector T cells in tumor biopsies and peripheral circulation, etc. may be evaluated if the data allow. These may be reported in separate biomarker data report as applicable.

9 STUDY OVERSIGHT AND DATA REPORTING/REGULATORY REQUIREMENTS

9.1 Regulatory and Ethical Considerations

1. This study will be conducted in accordance with the protocol and with the following:
    - Consensus ethical principles derived from international guidelines including the Declaration of Helsinki and Council for International Organizations of Medical Sciences (CIOMS) International Ethical Guidelines
    - Applicable ICH Good Clinical Practice (GCP) Guidelines
    - Applicable laws and regulations
2. The protocol, protocol amendments, ICF, IB, and other relevant documents (e.g., advertisements) must be submitted to an IRB/IEC by the investigator and reviewed and approved by the IRB/IEC and national regulatory authority (as applicable), before the study is initiated.
3. Any amendments to the protocol will require IRB/IEC and national regulatory authority (where required) approval before implementation of changes made to the study design, except for changes necessary to eliminate an immediate hazard to study participants.
    - Study Design is based on patient populations to diversify regionally the range of patients participating.
4. Protocols and any substantial amendments to the protocol will require IRB/IEC and national regulatory authority approval prior to initiation, except for changes necessary to eliminate an immediate hazard to study participants.
5. The investigator will be responsible for the following:
    - Providing written summaries of the status of the study to the IRB/IEC annually or more frequently in accordance with the requirements, policies, and procedures established by the IRB/IEC.
    - Notifying the IRB/IEC of SAEs or other significant safety findings as required by IRB/IEC procedures.
    - Providing oversight of the conduct of the study at the site and adherence to requirements of 21 CFR, ICH guidelines, the IRB/IEC, European regulation 536/2014 for clinical studies (if applicable), European Medical Device Regulation 2017/745 for clinical device research (if applicable), and all other applicable local regulations.

9.2 Financial Disclosure

Investigators and sub-investigators will provide the Sponsor with sufficient, accurate financial information as requested to allow the Sponsor to submit complete and accurate financial certification or disclosure statements to the appropriate regulatory authorities. Investigators are responsible for providing information on financial interests during the course of the study and for 1 year after completion of the study.

9.3 Informed Consent Process

The investigator or his/her representative will explain the nature of the study to the participant and answer all questions regarding the study.

Participants must be informed that their participation is voluntary. Participants will be required to sign informed consent and other related documents that meets the requirements of 21 CFR 50, local regulations, ICH guidelines, Health Insurance Portability and Accountability Act (HIPAA) requirements and other RA/EC, where applicable, and the IRB/IEC or study center.

The medical record must include a statement that written informed consent was obtained before the participant was enrolled in the study and the date the written consent was obtained. The authorized person obtaining the informed consent must also sign the ICF.

Participants must be re-consented to the most current version of the ICF(s) during their participation in the study.

A copy of the ICF(s) must be provided to the participant.

Participants who are rescreened (**Section 5.6.1**) are required to sign a new ICF.

If participants will be asked to consent to optional exploratory research using the remainder of mandatory samples, include text that addresses the use of remaining mandatory samples for optional exploratory research. The ICF will contain a separate section that addresses the use of remaining mandatory samples for optional exploratory research which may include genetic tests conducted on cDNA samples. The investigator or authorized designee will explain to each participant the objectives of the exploratory research. Participants will be told that they are free to refuse to participate and may withdraw their consent at any time and for any reason during the storage period. A separate signature will be required to document a participant's agreement to allow any remaining specimens to be used for exploratory research. Participants who decline to participate in this optional research will not provide this separate signature.

9.4 Data Protection

Participants will be assigned a unique identifier by the Sponsor. Any participant records or datasets that are transferred to the Sponsor will contain the identifier only; participant names or any information that would make the participant identifiable will not be transferred.

The participant must be informed that his/her personal study-related data will be used by the Sponsor in accordance with local data protection law. The level of disclosure must also be explained to the participant who will be required to give consent for their data to be used as described in the informed consent.

The participant must be informed that his/her medical records may be examined by Clinical Quality Assurance auditors or other authorized personnel appointed by the Sponsor, by appropriate IRB/IEC members, and by inspectors from regulatory authorities.

9.5 Committees Structure

Independent Data Monitoring Committee

An independent data monitoring committee (DMC) is planned to review safety data and CCI [redacted] make recommendations to the Sponsor. The members of the DMC are not involved with the trial or the trial investigators. The DMC will include oncologists who are experienced in lung cancer and a statistician. The composition and procedures of the DMC will be described in detail in the DMC Charter.

9.6 Dissemination of Clinical Study Data

As the Sponsor of the study, OncoC4 is solely responsible for disclosing results on ClinicalTrials.gov, EudraCT, and other public registries in accordance with applicable global laws and regulations. By

signing this protocol, the investigator acknowledges that all posting requirements are solely the responsibility of the Sponsor and agrees not to submit any information about the study or its results.

9.7 Data Quality Assurance

Investigators and site staff will be trained on protocol procedures and electronic case report form (eCRF) completion prior to enrolling participants in the study.

All participant data relating to the study will be recorded on printed or eCRF unless transmitted to the Sponsor or designee electronically (e.g., laboratory data). The investigator is responsible for verifying that data entries are accurate and correct by physically or electronically signing the CRF.

Guidance on completion of CRFs will be provided in data handling and entry guidelines.

The investigator must permit study-related monitoring, audits, IRB/IEC review, and regulatory agency inspections and provide direct access to source data documents.

Monitoring details describing strategy (e.g., risk-based initiatives in operations and quality such as Risk Management and Mitigation Strategies and Analytical Risk-Based Monitoring), methods, responsibilities, and requirements, including handling of noncompliance issues, and monitoring techniques (central, remote, or on-site monitoring) are provided in the Monitoring Plan. Cumulative monitoring of all study patients will occur at least every 6 months (semi-annual review).

The Sponsor or designee is responsible for the data management of this study, including quality checking of the data.

The Sponsor assumes accountability for actions delegated to other individuals (e.g., Contract Research Organizations).

Records and documents, including signed ICFs, pertaining to the conduct of this study must be retained by the investigator or institution/site as applicable, for the period of time established in the clinical study agreement entered into by the investigator's study site unless local regulations or institutional policies require a longer retention period. No records may be destroyed during the retention period without the written approval of the Sponsor. No records may be transferred to another location or party without written notification to the Sponsor.

The Sponsor or designee has implemented appropriate technical and organizational measures to protect information and personal data processed against unauthorized or unlawful access, disclosure, dissemination, alteration, destruction, or accidental loss, in particular where the processing involves the transmission. In case of data security breach, the Sponsor's data protection representative shall without undue delay and, where feasible, not later than 72 hours after having become aware of it, notify the data breach to the data protection authority, unless the data breach is unlikely to result in a risk to the rights and freedoms of participants. Subjects who are personally concerned and potentially endangered by a data protection breach are informed immediately.

In the event direct inspection is requested, the Investigator, Sponsor, or designee is to notify all affected parties of request within 24 hours, and records are to be made available for direct inspection, verification, and copying, as required by applicable laws and regulations, by officials of the regulatory health authorities. The investigator will comply with applicable privacy and security laws for use and disclosure of information.

9.8 Source Documents

Source documents provide evidence for the existence of the participant and substantiate the integrity of the data collected. Source documents are filed at the investigator's site.

Data reported on the CRF or entered in the eCRF that are transcribed from source documents must be consistent with the source documents or the discrepancies must be explained. The investigator may need to request previous medical records or transfer records. Also, current medical records must be available.

Definition of what constitutes source data can be found in data handling and entry guidelines.

The investigator must maintain accurate documentation (source data) that supports the information entered in the CRF.

Study monitors will perform ongoing source data verification to confirm that data entered into the CRF by authorized site personnel are accurate, complete, and verifiable from source documents; that the safety and rights of participants are being protected; and that the study is being conducted in accordance with the currently approved protocol, monitoring plans and any other study agreements, ICH GCP, and all applicable regulatory requirements.

9.9 Study and Site Start and Closure

9.9.1 First Act of Recruitment

The study start date is the date on which the first participant is randomized to treatment arm.

9.9.2 Study/Site Termination

The Sponsor or designee reserves the right to close the study site or terminate the study at any time for any reason at the sole discretion of the Sponsor. Study sites will be closed upon study completion. A study site is considered closed when all required documents and study supplies have been collected and a study site closure visit has been performed.

The investigator may initiate study site closure at any time, provided there is reasonable cause and sufficient notice is given in advance of the intended termination.

Reasons for the early closure of a study site by the Sponsor or investigator may include but are not limited to:

For study termination:

1. Discontinuation of further study intervention development

For site termination:

2. Failure of the investigator to comply with the protocol, the requirements of the IRB/IEC or local health authorities, the Sponsor's procedures, or GCP guidelines
3. Inadequate or no recruitment (evaluated after a reasonable amount of time) of participants by the investigator
4. Total number of participants included earlier than expected

If the study is prematurely terminated or suspended, the Sponsor shall promptly inform the investigators, the IECs/IRBs, the regulatory authorities, and any contract research organization(s) used in the study of the reason for termination or suspension, as specified by the applicable regulatory requirements. The investigator shall promptly inform the participant and should assure appropriate participant therapy and/or follow-up.

9.10 Publication Policy

Please refer to individual site contracts for specific contractual obligations and requirements.

All information concerning ONC-392, OncoC4's operations, patent applications, formulas, manufacturing processes, basic scientific data, and formulation information supplied by OncoC4 to the investigator and not previously published, are considered confidential and remain the sole property of OncoC4. eCRFs also remain the property of OncoC4. The investigator agrees to use this information only to complete this study and will not use it for other purposes without written consent of OncoC4 as further detailed in the Clinical Study Agreement signed by the investigator and/or institution.

It is understood by the investigator that OncoC4 will use the information obtained in this clinical trial in connection with the study of ONC-392, and therefore may disclose this information as required to other OncoC4 investigators; appropriate international regulatory agencies; or others. In agreeing to participate in this study, the investigator understands that he/she has an obligation to provide complete test results and all data developed during this trial to OncoC4. OncoC4 requires that permission to publish details of this study must be obtained in writing as further detailed in the Clinical Study Agreement signed by the investigator and/or institution. It is intended that the results of this trial will be published in scientific literature. The conditions noted here are intended to protect commercial confidential materials (patents, etc.) and not to restrict publication.

9.11 Study Medication Accountability

Study drug ONC-392 and all centrally sourced docetaxel required for completion of this study will be provided by the Sponsor. The proof of Investigational Product (IP) and Comparator drug delivery to the site is automatically sent to Almac via the courier, while the site is responsible for uploading the temperature monitoring data to TempEZ. Damaged supplies will be replaced.

Study drug accountability records should be maintained by the site in accordance with the regulations. The original drug supply request of ONC-392 will be submitted to the Sponsor or Sponsor's representative along with the form "Approval for Drug Re-Supply", indicating which personnel will be able to submit drug re-supply requests.

All subsequent drug re-supply requests will be directly submitted to the IP distributor, Almac, and the CRA from the Sponsor will be notified. At the time of study closure, the unused, used and expired study drug will be destroyed at the site per institutional SOPs or returned to the Sponsor or Sponsor's representative.

10 REFERENCES

1. Linsley, P.S.; Brady, W.; Urnes, M.; Grosmaire, L.S.; Damle, N.K.; Ledbetter, J.A. CTLA-4 Is a Second Receptor for the B Cell Activation Antigen B7. *J Exp Med* **1991**, *174*, 561–569, doi:10.1084/jem.174.3.561.

2. Freeman, G.J.; Gribben, J.G.; Boussiotis, V.A.; Ng, J.W.; Restivo, V.A.; Lombard, L.A.; Gray, G.S.; Nadler, L.M. Cloning of B7-2: A CTLA-4 Counter-Receptor That Costimulates Human T Cell Proliferation. *Science* **1993**, *262*, 909–911, doi:10.1126/science.7694363.

3. Wu, Y.; Guo, Y.; Liu, Y. A Major Costimulatory Molecule on Antigen-Presenting Cells, CTLA4 Ligand A, Is Distinct from B7. *J Exp Med* **1993**, *178*, 1789–1793, doi:10.1084/jem.178.5.1789.

4. Wing, K.; Onishi, Y.; Prieto-Martin, P.; Yamaguchi, T.; Miyara, M.; Fehervari, Z.; Nomura, T.; Sakaguchi, S. CTLA-4 Control over Foxp3+ Regulatory T Cell Function. *Science* **2008**, *322*, 271–275, doi:10.1126/science.1160062.

5. Kuehn, H.S.; Ouyang, W.; Lo, B.; Deenick, E.K.; Niemela, J.E.; Avery, D.T.; Schickel, J.-N.; Tran, D.Q.; Stoddard, J.; Zhang, Y.; et al. Immune Dysregulation in Human Subjects with Heterozygous Germline Mutations in CTLA4. *Science* **2014**, *345*, 1623–1627, doi:10.1126/science.1255904.

6. Tivol, E.A.; Borriello, F.; Schweitzer, A.N.; Lynch, W.P.; Bluestone, J.A.; Sharpe, A.H. Loss of CTLA-4 Leads to Massive Lymphoproliferation and Fatal Multiorgan Tissue Destruction, Revealing a Critical Negative Regulatory Role of CTLA-4. *Immunity* **1995**, *3*, 541–547, doi:10.1016/1074-7613(95)90125-6.

7. Waterhouse, P.; Penninger, J.M.; Timms, E.; Wakeham, A.; Shahinian, A.; Lee, K.P.; Thompson, C.B.; Griesser, H.; Mak, T.W. Lymphoproliferative Disorders with Early Lethality in Mice Deficient in Ctla-4. *Science* **1995**, *270*, 985–988, doi:10.1126/science.270.5238.985.

8. Hou, T.Z.; Verma, N.; Wanders, J.; Kennedy, A.; Soskic, B.; Janman, D.; Halliday, N.; Rowshanravan, B.; Worth, A.; Qasim, W.; et al. Identifying Functional Defects in Patients with Immune Dysregulation Due to LRBA and CTLA-4 Mutations. *Blood* **2017**, *129*, 1458–1468, doi:10.1182/blood-2016-10-745174.

9. Leach, D.R.; Krummel, M.F.; Allison, J.P. Enhancement of Antitumor Immunity by CTLA-4 Blockade. *Science* **1996**, *271*, 1734–1736, doi:10.1126/science.271.5256.1734.

10. Kocak, E.; Lute, K.; Chang, X.; May, K.F.; Exten, K.R.; Zhang, H.; Abdessalam, S.F.; Lehman, A.M.; Jarjoura, D.; Zheng, P.; et al. Combination Therapy with Anti-CTL Antigen-4 and Anti-4-1BB Antibodies Enhances Cancer Immunity and Reduces Autoimmunity. *Cancer Res* **2006**, *66*, 7276–7284, doi:10.1158/0008-5472.CAN-05-2128.

11. Mokyr, M.B.; Kalinichenko, T.; Gorelik, L.; Bluestone, J.A. Realization of the Therapeutic Potential of CTLA-4 Blockade in Low-Dose Chemotherapy-Treated Tumor-Bearing Mice. *Cancer Res* **1998**, *58*, 5301–5304.

12. Amaria, R.N.; Reddy, S.M.; Tawbi, H.A.; Davies, M.A.; Ross, M.I.; Glitza, I.C.; Cormier, J.N.; Lewis, C.; Hwu, W.-J.; Hanna, E.; et al. Neoadjuvant Immune Checkpoint Blockade in High-Risk Resectable Melanoma. *Nat Med* **2018**, *24*, 1649–1654, doi:10.1038/s41591-018-0197-1.

13. Hellmann, M.D.; Ciuleanu, T.-E.; Pluzanski, A.; Lee, J.S.; Otterson, G.A.; Audigier-Valette, C.; Minenza, E.; Linardou, H.; Burgers, S.; Salman, P.; et al. Nivolumab plus Ipilimumab in Lung Cancer with a High Tumor Mutational Burden. *N Engl J Med* **2018**, *378*, 2093–2104, doi:10.1056/NEJMoa1801946.

14. Wei, S.C.; Duffy, C.R.; Allison, J.P. Fundamental Mechanisms of Immune Checkpoint Blockade Therapy. *Cancer Discov* **2018**, *8*, 1069–1086, doi:10.1158/2159-8290.CD-18-0367.

15. Weber, J.; Mandala, M.; Del Vecchio, M.; Gogas, H.J.; Arance, A.M.; Cowey, C.L.; Dalle, S.; Schenker, M.; Chiarion-Sileni, V.; Marquez-Rodas, I.; et al. Adjuvant Nivolumab versus Ipilimumab in Resected Stage III or IV Melanoma. *N Engl J Med* **2017**, *377*, 1824–1835, doi:10.1056/NEJMoa1709030.

16. Blank, C.U.; Rozeman, E.A.; Fanchi, L.F.; Sikorska, K.; van de Wiel, B.; Kvistborg, P.; Krijgsman, O.; van den Braber, M.; Philips, D.; Broeks, A.; et al. Neoadjuvant versus Adjuvant Ipilimumab plus Nivolumab in Macroscopic Stage III Melanoma. *Nat Med* **2018**, *24*, 1655–1661, doi:10.1038/s41591-018-0198-0.

17. Maio, M.; Grob, J.-J.; Aamdal, S.; Bondarenko, I.; Robert, C.; Thomas, L.; Garbe, C.; Chiarion-Sileni, V.; Testori, A.; Chen, T.-T.; et al. Five-Year Survival Rates for Treatment-Naive Patients with Advanced Melanoma Who Received Ipilimumab plus Dacarbazine in a Phase III Trial. *J Clin Oncol* **2015**, *33*, 1191–1196, doi:10.1200/JCO.2014.56.6018.

18. Wolchok, J.D.; Kluger, H.; Callahan, M.K.; Postow, M.A.; Rizvi, N.A.; Lesokhin, A.M.; Segal, N.H.; Ariyan, C.E.; Gordon, R.-A.; Reed, K.; et al. Nivolumab plus Ipilimumab in Advanced Melanoma. *N Engl J Med* **2013**, *369*, 122–133, doi:10.1056/NEJMoa1302369.

19. Larkin, J.; Chiarion-Sileni, V.; Gonzalez, R.; Grob, J.J.; Cowey, C.L.; Lao, C.D.; Schadendorf, D.; Dummer, R.; Smylie, M.; Rutkowski, P.; et al. Combined Nivolumab and Ipilimumab or Monotherapy in Untreated Melanoma. *N Engl J Med* **2015**, *373*, 23–34, doi:10.1056/NEJMoa1504030.

20. Motzer, R.J.; Tannir, N.M.; McDermott, D.F.; Arén Frontera, O.; Melichar, B.; Choueiri, T.K.; Plimack, E.R.; Barthélémy, P.; Porta, C.; George, S.; et al. Nivolumab plus Ipilimumab versus Sunitinib in Advanced Renal-Cell Carcinoma. *N Engl J Med* **2018**, *378*, 1277–1290, doi:10.1056/NEJMoa1712126.

21. Schadendorf, D.; Hodi, F.S.; Robert, C.; Weber, J.S.; Margolin, K.; Hamid, O.; Patt, D.; Chen, T.-T.; Berman, D.M.; Wolchok, J.D. Pooled Analysis of Long-Term Survival Data From Phase II and Phase III Trials of Ipilimumab in Unresectable or Metastatic Melanoma. *J Clin Oncol* **2015**, *33*, 1889–1894, doi:10.1200/JCO.2014.56.2736.

22. Korman, A.J.; Peggs, K.S.; Allison, J.P. Checkpoint Blockade in Cancer Immunotherapy. *Adv Immunol* **2006**, *90*, 297–339, doi:10.1016/S0065-2776(06)90008-X.

23. Du, X.; Liu, M.; Su, J.; Zhang, P.; Tang, F.; Ye, P.; Devenport, M.; Wang, X.; Zhang, Y.; Liu, Y.; et al. Uncoupling Therapeutic from Immunotherapy-Related Adverse Effects for Safer and Effective Anti-CTLA-4 Antibodies in CTLA4 Humanized Mice. *Cell Res* **2018**, *28*, 433–447, doi:10.1038/s41422-018-0012-z.

24. CCI [redacted]

25. Simpson, T.R.; Li, F.; Montalvo-Ortiz, W.; Sepulveda, M.A.; Bergerhoff, K.; Arce, F.; Roddie, C.; Henry, J.Y.; Yagita, H.; Wolchok, J.D.; et al. Fc-Dependent Depletion of Tumor-Infiltrating Regulatory T Cells Co-Defines the Efficacy of Anti-CTLA-4 Therapy against Melanoma. *J Exp Med* **2013**, *210*, 1695–1710, doi:10.1084/jem.20130579.

26. Bulliard, Y.; Jolicoeur, R.; Windman, M.; Rue, S.M.; Ettenberg, S.; Knee, D.A.; Wilson, N.S.; Dranoff, G.; Brogdon, J.L. Activating Fc γ Receptors Contribute to the Antitumor Activities of Immunoregulatory Receptor-Targeting Antibodies. *J Exp Med* **2013**, *210*, 1685–1693, doi:10.1084/jem.20130573.

27. Lute, K.D.; May, K.F.; Lu, P.; Zhang, H.; Kocak, E.; Mosinger, B.; Wolford, C.; Phillips, G.; Caligiuri, M.A.; Zheng, P.; et al. Human CTLA4 Knock-in Mice Unravel the Quantitative Link between Tumor Immunity and Autoimmunity Induced by Anti-CTLA-4 Antibodies. *Blood* **2005**, *106*, 3127–3133, doi:10.1182/blood-2005-06-2298.

28. May, K.F.; Roychowdhury, S.; Bhatt, D.; Kocak, E.; Bai, X.-F.; Liu, J.-Q.; Ferketich, A.K.; Martin, E.W.; Caligiuri, M.A.; Zheng, P.; et al. Anti-Human CTLA-4 Monoclonal Antibody Promotes T-Cell Expansion and Immunity in a Hu-PBL-SCID Model: A New Method for Preclinical Screening of Costimulatory Monoclonal Antibodies. *Blood* **2005**, *105*, 1114–1120, doi:10.1182/blood-2004-07-2561.

29. Shields, R.L.; Namenuk, A.K.; Hong, K.; Meng, Y.G.; Rae, J.; Briggs, J.; Xie, D.; Lai, J.; Stadlen, A.; Li, B.; et al. High Resolution Mapping of the Binding Site on Human IgG1 for Fc Gamma RI, Fc Gamma RII, Fc Gamma RIII, and FcRn and Design of IgG1 Variants with Improved Binding to the Fc Gamma R. *J Biol Chem* **2001**, *276*, 6591–6604, doi:10.1074/jbc.M009483200.

30. Zhang, Y.; Du, X.; Liu, M.; Tang, F.; Zhang, P.; Ai, C.; Fields, J.K.; Sundberg, E.J.; Latinovic, O.S.; Devenport, M.; et al. Hijacking Antibody-Induced CTLA-4 Lysosomal Degradation for Safer and More Effective Cancer Immunotherapy. *Cell Res* **2019**, *29*, 609–627, doi:10.1038/s41422-019-0184-1.

31. Linsley, P.S.; Bradshaw, J.; Greene, J.; Peach, R.; Bennett, K.L.; Mittler, R.S. Intracellular Trafficking of CTLA-4 and Focal Localization towards Sites of TCR Engagement. *Immunity* **1996**, *4*, 535–543, doi:10.1016/s1074-7613(00)80480-x.

32. Lo, B.; Zhang, K.; Lu, W.; Zheng, L.; Zhang, Q.; Kanellopoulou, C.; Zhang, Y.; Liu, Z.; Fritz, J.M.; Marsh, R.; et al. AUTOIMMUNE DISEASE. Patients with LRBA Deficiency Show CTLA4 Loss and Immune Dysregulation Responsive to Abatacept Therapy. *Science* **2015**, *349*, 436–440, doi:10.1126/science.aaa1663.

33. FDA Guidance for Industry Cancer Clinical Trial Eligibility Criteria: Available Therapy in Non-Curative Settings. 2022.

34. Pauken, K.E.; Wherry, E.J. Overcoming T Cell Exhaustion in Infection and Cancer. *Trends Immunol* **2015**, *36*, 265–276, doi:10.1016/j.it.2015.02.008.

35. Brahmer, J.; Reckamp, K.L.; Baas, P.; Crinò, L.; Eberhardt, W.E.E.; Poddubskaya, E.; Antonia, S.; Pluzanski, A.; Vokes, E.E.; Holgado, E.; et al. Nivolumab versus Docetaxel in Advanced Squamous-Cell Non-Small-Cell Lung Cancer. *N Engl J Med* **2015**, *373*, 123–135, doi:10.1056/NEJMoa1504627.

36. Borghaei, H.; Paz-Ares, L.; Horn, L.; Spigel, D.R.; Steins, M.; Ready, N.E.; Chow, L.Q.; Vokes, E.E.; Felip, E.; Holgado, E.; et al. Nivolumab versus Docetaxel in Advanced Nonsquamous Non–Small-Cell Lung Cancer. *N Engl J Med* **2015**, *373*, 1627–1639, doi:10.1056/NEJMoa1507643.

37. Herbst, R.S.; Baas, P.; Kim, D.-W.; Felip, E.; Pérez-Gracia, J.L.; Han, J.-Y.; Molina, J.; Kim, J.-H.; Arvis, C.D.; Ahn, M.-J.; et al. Pembrolizumab versus Docetaxel for Previously Treated, PD-L1-Positive, Advanced Non-Small-Cell Lung Cancer (KEYNOTE-010): A Randomised Controlled Trial. *Lancet* **2016**, *387*, 1540–1550, doi:10.1016/S0140-6736(15)01281-7.

38. Cortellini, A.; Cannita, K.; Tiseo, M.; Cortinovis, D.L.; Aerts, J.G.J.V.; Baldessari, C.; Giusti, R.; Ferrara, M.G.; D'Argento, E.; Grossi, F.; et al. Post-Progression Outcomes of NSCLC Patients with PD-L1 Expression ≥ 50% Receiving First-Line Single-Agent Pembrolizumab in a Large Multicentre Real-World Study. *Eur J Cancer* **2021**, *148*, 24–35, doi:10.1016/j.ejca.2021.02.005.

39. Bersanelli, M.; Buti, S.; Giannarelli, D.; Leonetti, A.; Cortellini, A.; Russo, G.L.; Signorelli, D.; Toschi, L.; Milella, M.; Pilotto, S.; et al. Chemotherapy in Non-Small Cell Lung Cancer Patients after Prior Immunotherapy: The Multicenter Retrospective CLARITY Study. *Lung Cancer* **2020**, *150*, 123–131, doi:10.1016/j.lungcan.2020.10.008.

40. Ettinger, D.S.; Wood, D.E.; Aisner, D.L.; Akerley, W.; Bauman, J.R.; Bharat, A.; Bruno, D.S.; Chang, J.Y.; Chirieac, L.R.; D'Amico, T.A.; et al. Non-Small Cell Lung Cancer, Version 3.2022, NCCN Clinical Practice Guidelines in Oncology. *J Natl Compr Canc Netw* **2022**, *20*, 497–530, doi:10.6004/jnccn.2022.0025.

41. Singh, N.; Temin, S.; Baker, S.; Blanchard, E.; Brahmer, J.R.; Celano, P.; Duma, N.; Ellis, P.M.; Elkins, I.B.; Haddad, R.Y.; et al. Therapy for Stage IV Non-Small-Cell Lung Cancer Without Driver Alterations: ASCO Living Guideline. *J Clin Oncol* **2022**, *40*, 3323–3343, doi:10.1200/JCO.22.00825.

42. Davies, J.; Patel, M.; Gridelli, C.; de Marinis, F.; Waterkamp, D.; McCusker, M.E. Real-World Treatment Patterns for Patients Receiving Second-Line and Third-Line Treatment for Advanced Non-Small Cell Lung Cancer: A Systematic Review of Recently Published Studies. *PLoS One* **2017**, *12*, e0175679, doi:10.1371/journal.pone.0175679.

43. Bains, S.; Kalsekar, A.; Amiri, K.I.; Weiss, J. Real-World Treatment Patterns and Outcomes Among Patients With Metastatic NSCLC Previously Treated With Programmed Cell Death Protein-1/Programmed Death-Ligand 1 Inhibitors. *JTO Clin Res Rep* **2022**, *3*, 100275, doi:10.1016/j.jtocrr.2021.100275.

44. Leighl, N.B.; Redman, M.W.; Rizvi, N.; Hirsch, F.R.; Mack, P.C.; Schwartz, L.H.; Wade, J.L.; Irvin, W.J.; Reddy, S.C.; Crawford, J.; et al. Phase II Study of Durvalumab plus Tremelimumab as Therapy for Patients with Previously Treated Anti-PD-1/PD-L1 Resistant Stage IV Squamous Cell Lung Cancer (Lung-MAP Substudy S1400F, NCT03373760). *J Immunother Cancer* **2021**, *9*, e002973, doi:10.1136/jitc-2021-002973.

45. Postoperative T1 N0 Non-Small Cell Lung Cancer. Squamous versus Nonsquamous Recurrences. The Lung Cancer Study Group. *J Thorac Cardiovasc Surg* **1987**, *94*, 349–354.

46. Ashworth, A.B.; Senan, S.; Palma, D.A.; Riquet, M.; Ahn, Y.C.; Ricardi, U.; Congedo, M.T.; Gomez, D.R.; Wright, G.M.; Melloni, G.; et al. An Individual Patient Data Metaanalysis of Outcomes and Prognostic Factors after Treatment of Oligometastatic Non-Small-Cell Lung Cancer. *Clin Lung Cancer* **2014**, *15*, 346–355, doi:10.1016/j.cllc.2014.04.003.

47. Fidias, P.M.; Dakhil, S.R.; Lyss, A.P.; Loesch, D.M.; Waterhouse, D.M.; Bromund, J.L.; Chen, R.; Hristova-Kazmierski, M.; Treat, J.; Obasaju, C.K.; et al. Phase III Study of Immediate Compared with Delayed Docetaxel after Front-Line Therapy with Gemcitabine plus Carboplatin in Advanced Non-Small-Cell Lung Cancer. *J Clin Oncol* **2009**, *27*, 591–598, doi:10.1200/JCO.2008.17.1405.

48. Dolan, R.D.; Daly, L.; Sim, W.M.J.; Fallon, M.; Ryan, A.; McMillan, D.C.; Laird, B.J. Comparison of the Prognostic Value of ECOG-PS, mGPS and BMI/WL: Implications for a Clinically Important Framework in the Assessment and Treatment of Advanced Cancer. *Clinical Nutrition* **2020**, *39*, 2889–2895, doi:10.1016/j.clnu.2019.12.024.

49. Hoang, T.; Xu, R.; Schiller, J.H.; Bonomi, P.; Johnson, D.H. Clinical Model to Predict Survival in Chemonaive Patients with Advanced Non-Small-Cell Lung Cancer Treated with Third-Generation Chemotherapy Regimens Based on Eastern Cooperative Oncology Group Data. *J Clin Oncol* **2005**, *23*, 175–183, doi:10.1200/JCO.2005.04.177.

50. CCI [redacted]

51. CCI [redacted]

52. CCI [redacted]

53. CCI [redacted]

54. CCI [redacted]

55. Malinou, J.N.; Mehta, G.; Vellanki, P.J.; Larkins, E.A.; Kluetz, P.G.; Singh, H. FDA Analysis of Immune Checkpoint Inhibitors in Combination with Vascular Endothelial Growth Factor Tyrosine Kinase Inhibitors in the Second-Line Treatment of Patients with Advanced Non-Small Cell Lung Cancer. *JCO* **2024**, *42*, 8595–8595, doi:10.1200/JCO.2024.42.16_suppl.8595.

56. Han, B.; Shi, Y.-K.; Feinstein, T.; Feng, D.; Mitchell, D.; Lelorier, Y.; Du, L.; Huang, L.; Mohanlal, R.; Sun, Y. LBA48 DUBLIN-3 (BPI-2358-103): A Global Phase (Ph) III Trial with the Plinabulin/Docetaxel (Plin/Doc) Combination vs. Doc in 2nd/3rd Line NSCLC Patients (Pts) with EGFR-Wild Type (Wt) Progressing on a Prior Platinum-Based Regimen. *Annals of Oncology* **2021**, *32*, S1326, doi:10.1016/j.annonc.2021.08.2127.

57. CCI [redacted]

58. Eisenhauer, E.A.; Therasse, P.; Bogaerts, J.; Schwartz, L.H.; Sargent, D.; Ford, R.; Dancey, J.; Arbuck, S.; Gwyther, S.; Mooney, M.; et al. New Response Evaluation Criteria in Solid Tumours: Revised RECIST Guideline (Version 1.1). *Eur J Cancer* **2009**, *45*, 228–247, doi:10.1016/j.ejca.2008.10.026.

59. Aaronson, N.K.; Bullinger, M.; Ahmedzai, S. A Modular Approach to Quality-of-Life Assessment in Cancer Clinical Trials. *Recent Results Cancer Res* **1988**, *111*, 231–249, doi:10.1007/978-3-642-83419-6_27.

60. Koller, M.; Hjermstad, M.J.; Tomaszewski, K.A.; Tomaszewska, I.M.; Hornslien, K.; Harle, A.; Arraras, J.I.; Morag, O.; Pompili, C.; Ioannidis, G.; et al. An International Study to Revise the

EORTC Questionnaire for Assessing Quality of Life in Lung Cancer Patients. *Ann Oncol* **2017**, *28*, 2874–2881, doi:10.1093/annonc/mdx453.

61. McCarrier, K.P.; Atkinson, T.M.; DeBusk, K.P.A.; Liepa, A.M.; Scanlon, M.; Coons, S.J.; Patient-Reported Outcome Consortium, Non–Small Cell Lung Cancer Working Group Qualitative Development and Content Validity of the Non-Small Cell Lung Cancer Symptom Assessment Questionnaire (NSCLC-SAQ), A Patient-Reported Outcome Instrument. *Clin Ther* **2016**, *38*, 794–810, doi:10.1016/j.clinthera.2016.03.012.

62. Herdman, M.; Gudex, C.; Lloyd, A.; Janssen, M.; Kind, P.; Parkin, D.; Bonsel, G.; Badia, X. Development and Preliminary Testing of the New Five-Level Version of EQ-5D (EQ-5D-5L). *Qual Life Res* **2011**, *20*, 1727–1736, doi:10.1007/s11136-011-9903-x.

63. Zou, F.; Faleck, D.; Thomas, A.; Harris, J.; Satish, D.; Wang, X.; Charabaty, A.; Ernstoff, M.S.; Glitza Oliva, I.C.; Hanauer, S.; et al. Efficacy and Safety of Vedolizumab and Infliximab Treatment for Immune-Mediated Diarrhea and Colitis in Patients with Cancer: A Two-Center Observational Study. *J Immunother Cancer* **2021**, *9*, e003277, doi:10.1136/jitc-2021-003277.

64. CTFG. Recommendations Related to Contraception and Pregnancy Testing in Clinical Trials, Version 1.1 2014.

11 Appendix 1

Contraceptive and Barrier Guidance and Collection of Pregnancy Information

Definitions

Woman of Childbearing Potential (WOCBP)

A woman is considered fertile following menarche and until becoming postmenopausal unless permanently sterile (see below).

Women in the following categories are not considered WOCBP

1. Premenarchal
2. Premenopausal female with 1 of the following:
   a) Documented hysterectomy.
   b) Documented bilateral salpingectomy.
   c) Documented bilateral oophorectomy.

NOTE: Documentation can come from the study site personnel's: review of the participant's medical records, medical examination, or medical history interview.

3. Postmenopausal female:
   a) A postmenopausal state is defined as no menses for 12 months without an alternative medical cause. A high follicle-stimulating hormone (FSH) level in the postmenopausal range may be used to confirm a postmenopausal state in women not using hormonal contraception or hormonal replacement therapy (HRT). However, in the absence of 12 months of amenorrhea, a single FSH measurement is insufficient.
   b) Females on HRT and whose menopausal status is in doubt will be required to use 1 of the non-estrogen hormonal highly effective contraception methods if they wish to continue their HRT during the study. Otherwise, they must discontinue HRT to allow confirmation of postmenopausal status before study enrollment.

Contraception Guidance

Male participants

- Male participants with female partners of childbearing potential are eligible to participate if they agree to ONE of the following from the screening visit to 6 months after last treatment on study drug:
  - Are abstinent from penile-vaginal intercourse as their usual and preferred lifestyle (abstinent on a long-term and persistent basis) and agree to remain abstinent for duration of study and for 6 months after last treatment on study drug.
  - Agree to use a male condom and have their partner use a contraceptive method with a failure rate of <1% per year as described in Table below when having penile-vaginal intercourse with a woman of childbearing potential who is not currently pregnant.
- In addition, male participants must refrain from donating sperm for the duration of the study and up to 6 months after the last dose of study intervention.
- Male participants with a pregnant or breastfeeding partner should not be enrolled in the study.

- Male participants are advised to seek guidance on the conservation of sperm prior to treatment.

Female participants

Female participants of childbearing potential are eligible to participate if they agree to use a highly effective method of contraception as per local guidelines and requirement [64] consistently and correctly as described in the table below from the screening visit to 6 months after last treatment on study drug.

Highly Effective Contraceptive Methods

**Highly Effective Contraceptive Methods That Are User Dependent[a]**<br>*Failure rate of <1% per year when used consistently and correctly.*
Combined (estrogen and progestogen containing) hormonal contraception associated with inhibition of ovulation<br>• Oral.<br>• Intravaginal.<br>• Transdermal.
Progestogen only hormonal contraception associated with inhibition of ovulation<br>• Oral.<br>• Injectable.<br>• Implantable.
**Highly Effective Methods That Are User Independent[a]**
Implantable progestogen only hormonal contraception associated with inhibition of ovulation<br>• Intrauterine device.<br>• Intrauterine hormone-releasing system.<br>• Bilateral tubal occlusion.
**Vasectomized partner**<br>*A vasectomized partner is a highly effective birth control method provided that the partner is the sole male sexual partner of the WOCBP and the absence of sperm has been confirmed. If not, an additional highly effective method of contraception should be used.*
**Sexual abstinence**<br>*Sexual abstinence is considered a highly effective method only if defined as refraining from heterosexual intercourse during the entire period of risk associated with the study intervention. The reliability of sexual abstinence needs to be evaluated in relation to the duration of the study and the preferred and usual lifestyle of the participant.*
NOTES:<br>[a] Typical use failure rates may differ from those when used consistently and correctly. Use should be consistent with local regulations regarding the use of contraceptive methods for participants participating in clinical studies.

Pregnancy Testing:

- Urine or serum testing are acceptable for WOCBP. Serum pregnancy test should be performed at screening.

- Urine pregnancy test will be done locally. Central testing of serum β-HCG should be done for an indeterminate test or to confirm a positive urine test.
- The pregnancy test should be performed at the screening and the end of study (EOT) visit, if applicable.
- WOCBP should only be included after a confirmed menstrual period and a negative serum pregnancy test.
- Pregnancy testing will be performed whenever a menstrual cycle is missed or when pregnancy is otherwise suspected.
- If required by regulatory authorities and/or ethics committee, pregnancy test will be performed at least monthly and prior to all imaging examination for female subjects with child-bearing potential.

Collection of Pregnancy Information

Male participants with partners who become pregnant

- The investigator will attempt to collect pregnancy information on any male participant's female partner who becomes pregnant while the male participant is in this study. This applies only to male participants who receive study drug.
- After obtaining the necessary signed informed consent from the pregnant female partner directly, the investigator will record pregnancy information on the appropriate form and submit it to the Sponsor or designee within 24 hours of learning of the partner's pregnancy. The female partner will also be followed to determine the outcome of the pregnancy. Information on the status of the mother and child will be forwarded to the Sponsor. Generally, the follow-up will be no longer than 6 to 8 weeks following the estimated delivery date. Any termination of the pregnancy will be reported regardless of fetal status (presence or absence of anomalies) or indication for the procedure.

Female Participants who become pregnant

- The investigator will collect pregnancy information on any female participant who becomes pregnant while participating in this study. Information will be recorded on the appropriate form and submitted to the Sponsor or designee within 24 hours of learning of a participant's pregnancy. The participant will be followed to determine the outcome of the pregnancy. The investigator will collect follow-up information on the participant and the neonate and the information will be forwarded to the Sponsor or designee. Generally, follow-up will not be required for longer than 6 to 8 weeks beyond the estimated delivery date. Any termination of pregnancy will be reported, regardless of fetal status (presence or absence of anomalies) or indication for the procedure.
- While pregnancy itself is not considered to be an AE or SAE, any pregnancy complication will be reported as an AE or SAE. If the outcome of the pregnancy meets the criteria for immediate classification as an SAE (i.e., spontaneous abortion, stillbirth, neonatal death, or congenital anomaly [including that in an aborted fetus, stillbirth, or neonatal death]), the investigator will report according to the SAE reporting procedures described in **Section 7.5** in protocol.
- Any post-study pregnancy-related SAE considered reasonably related to the study treatment by the investigator will be reported to the Sponsor or designee. While the investigator is not obligated to actively seek this information in former participants, he or she may learn of an SAE through spontaneous reporting.
- Any female participant who becomes pregnant while participating in the study will be discontinued in the treatment.

- Reporting Pregnancy Information

Email transmission of the paper Pregnancy Report Form is the preferred method to transmit safety event information to IQVIA Biotech with facsimile as a back-up method, if necessary.

Safety events should be reported to IQVIA Biotech at:

Email: [redacted]

Fax: [redacted]

12 Appendix 2

The exclusion criteria #4 from **Section 4.2** states the exclusion on patients carrying several targetable mutations or genomic alterations.

"#4. Patients who have documented non-squamous histology type or with targetable mutations or genomic alterations in the following genes: EGFR, ROS1, MET, BRAF, RET, NTRK, ALK or HER2. Patients with mutations or genomic alterations in KRAS are not excluded".

Here we clarify that we would follow the NCCN Guidelines (www.nccn.org) Non-small Cell Lung Cancer for definition of "targetable mutations or genomic alterations" except for KRAS G12C mutation.

Patients with mutations or genomic alterations in KRAS are not excluded. KRAS inhibitor treatment is optional and can be the line of treatment prior or after the line of treatment containing anti-PD-1 or anti-PD-L1 antibody.

13 Summary of Changes

[redacted]

CCI

[redacted]

CCI

[redacted]

CCI

[redacted]

CCI [redacted]

CCI [redacted]

CCI

[redacted]
