# Supplementary material for: Gotistobart or docetaxel in metastatic squamous non-small cell lung cancer: stage 1 of the randomized phase 3 PRESERVE-003 trial
Source: Nat Med. 2026 Mar 27;32(6):2245–53. doi: 10.1038/s41591-026-04323-8 (PMC13278949; doi:10.1038/s41591-026-04323-8)
Supplement: Supplementary file 2 — Reporting Summary [file 41591_2026_4323_MOESM2_ESM.pdf]

nature portfolio

Corresponding author(s): Yi-Long Wu

Last updated by author(s): 5/3/2026

Reporting Summary

Nature Portfolio wishes to improve the reproducibility of the work that we publish. This form provides structure for consistency and transparency in reporting. For further information on Nature Portfolio policies, see our Editorial Policies and the Editorial Policy Checklist.

Statistics

For all statistical analyses, confirm that the following items are present in the figure legend, table legend, main text, or Methods section.

n/a	Confirmed	
☐	☒	The exact sample size (*n*) for each experimental group/condition, given as a discrete number and unit of measurement
☐	☒	A statement on whether measurements were taken from distinct samples or whether the same sample was measured repeatedly
☐	☒	The statistical test(s) used AND whether they are one- or two-sided<br>*Only common tests should be described solely by name; describe more complex techniques in the Methods section.*
☐	☒	A description of all covariates tested
☒	☐	A description of any assumptions or corrections, such as tests of normality and adjustment for multiple comparisons
☐	☒	A full description of the statistical parameters including central tendency (e.g. means) or other basic estimates (e.g. regression coefficient) AND variation (e.g. standard deviation) or associated estimates of uncertainty (e.g. confidence intervals)
☐	☒	For null hypothesis testing, the test statistic (e.g. *F*, *t*, *r*) with confidence intervals, effect sizes, degrees of freedom and *P* value noted<br>*Give P values as exact values whenever suitable.*
☒	☐	For Bayesian analysis, information on the choice of priors and Markov chain Monte Carlo settings
☒	☐	For hierarchical and complex designs, identification of the appropriate level for tests and full reporting of outcomes
☒	☐	Estimates of effect sizes (e.g. Cohen's *d*, Pearson's *r*), indicating how they were calculated

Our web collection on statistics for biologists contains articles on many of the points above.

Software and code

Policy information about availability of computer code

Data collection	Data collection and query handling was managed using an electronic data capture (EDC) system (CONFORMTM, EDETEK, New Jersey, USA)
Data analysis	SAS (version 9.4 or later)

For manuscripts utilizing custom algorithms or software that are central to the research but not yet described in published literature, software must be made available to editors and reviewers. We strongly encourage code deposition in a community repository (e.g. GitHub). See the Nature Portfolio guidelines for submitting code & software for further information.

Data

Policy information about availability of data

All manuscripts must include a data availability statement. This statement should provide the following information, where applicable:

- Accession codes, unique identifiers, or web links for publicly available datasets
- A description of any restrictions on data availability
- For clinical datasets or third party data, please ensure that the statement adheres to our policy

This trial is currently ongoing. Upon completion of this clinical trial, the data that support the findings of this study will be made available to qualified researchers. Proposals should be directed to pzheng@oncoc4.com. To gain access, data requestors will need to sign a data access agreement. All data provided will be anonymized to respect the privacy of patients who have participated in the trial, in line with applicable laws and regulations. Data will be made available, upon request to qualified researchers, within 12 months of the study completion data and for a period of 2 years thereafter.

Research involving human participants, their data, or biological material

Policy information about studies with human participants or human data. See also policy information about sex, gender (identity/presentation), and sexual orientation and race, ethnicity and racism.

Reporting on sex and gender	Sex was patient-reported and based on medical records. Sex and gender were not considered in the study design; sex-based analyses were not performed as the study was not powered to do so.
Reporting on race, ethnicity, or other socially relevant groupings	Patient demographic information inclusive of race was collected at screening to aid in analyzing pharmacokinetic and safety data to determine if various aspects correlate to exposure/response. Patients had the right to decline to respond to race/ethnicity should they chose to do so. The submitted manuscript does not present any analyses by race.
Population characteristics	The population was as defined in the protocol-specified inclusion and exclusion criteria: eligible patients were ≥18 years of age with histologically or cytologically confirmed diagnosis of metastatic non-squamous NSCLC (Stage 1) or squamous NSCLC (Stage 1 and Stage 2). NSCLC with targetable mutations or genomic alterations in epidermal growth factor receptor (EGFR), proto-oncogene tyrosine-protein kinase-1 (ROS1), mesenchymal-to-epithelial transition protein (MET), B-raf proto-oncogene (BRAF), rearranged during transfection (RET), neurotrophic tyrosine receptor kinase (NTRK), anaplastic lymphoma kinase protein (ALK) or human epidermal growth factor receptor 2 (HER2) was not permitted. Patients must have had radiographic disease progression after the most recent treatment of either standard dose PD-(L)1 inhibitor (for ≥12 weeks) in combination with PBC or ≥2 cycles of PBC followed by standard dose PD-(L)1 inhibitor (for ≥12 weeks). Prior treatment with PD-(L)1 inhibitor combinations with anti-CTLA-4, anti-lymphocyte activation gene-3 (LAG-3), T-cell immunoreceptor with Ig and immunoreceptor tyrosine-based inhibitory motif domains (TIGIT), VEGF or VEGFR antibodies was permitted. Patients must have had measurable disease per Response Evaluation Criteria in Solid Tumors (RECIST) 1.1 and Eastern Cooperative Oncology Group performance status (ECOG PS) 0 or 1, with adequate organ function and a life expectancy of at least 3 months. The presence of active or symptomatic brain metastases or evidence of progression within 4 weeks prior to study drug dosing excluded patients from the study. Current interstitial lung disease or non-infectious pneumonitis, or clinically significant pleural effusion within 28 days prior to study drug dosing excluded patients from the study, as did history of interstitial lung disease or non-infectious pneumonitis associated with prior therapy and that required management with a high-dose steroid. Other exclusion criteria included AEs due to cancer treatment that had not recovered to National Cancer Institute Common Terminology for Adverse Events v5.0 (NCI CTCAE) Grade ≤1 except endocrinopathy or peripheral neuropathy in which recovery to Grade ≤2 was allowed, last anti-PD-1/PD-L1 dose within 28 days of first dose of study treatment, and receipt of systemic steroid therapy with >10 mg/day prednisone or equivalent within 7 days of the first dose of study treatment.
Recruitment	Patients were recruited by the Study Investigators according to the protocol-specified inclusion and exclusion criteria (see Population Characteristics section above). All patients provided written informed consent before enrolment.Because participation required eligibility under these criteria, willingness to enroll in an international randomized trial, and access to participating centers, selection and self selection biases are possible. Enrolled patients may be fitter, more treatment motivated and more closely monitored than the broader metastatic NSCLC population, and the exclusion of patients with targetable oncogenic drivers and significant comorbidities further limits generalizability. However, within the enrolled population, randomization and stratification (by histology, brain metastases, ECOG PS and region) minimize selection bias between treatment arms, so internal validity of the comparative efficacy and safety results is preserved, while external validity is primarily affected.
Ethics oversight	PRESERVE-003 was conducted in accordance with local and national regulations, together with consensus ethical principles derived from the Declaration of Helsinki and the Council for International Organizations of Medical Sciences International Ethical Guidelines and International Council Harmonisation Good Clinical Practice Guidelines. All study procedures, protocols and other relevant documents were approved by the institutional review board/ethics committee and national regulatory authority. The central Western Institutional Review Board provided approval in the United States. In Australia, China, Korea, and the United Kingdom the independent review boards or ethics committees at each study site provided approval.

Note that full information on the approval of the study protocol must also be provided in the manuscript.

Field-specific reporting

Please select the one below that is the best fit for your research. If you are not sure, read the appropriate sections before making your selection.

☒ Life sciences ☐ Behavioural & social sciences ☐ Ecological, evolutionary & environmental sciences

For a reference copy of the document with all sections, see nature.com/documents/nr-reporting-summary-flat.pdf

Life sciences study design

All studies must disclose on these points even when the disclosure is negative.

Sample size	The non-pivotal Stage 1 efficacy analysis included 87 with squamous NSCLC. One patients in the docetaxel arm did not receive treatment and so was not included in the analysis of safety. Data are also reported for 126 patients had non-sqNSCLC and patients with mixed histology who received gotistobart 3 mg/kg Q3W (n=10). The reported Stage 1 of the study was exploratory; sample size was not based on statistical requirements.

Data exclusions	No data were excluded. One patient in the docetaxel arm (sq NSCLC) did not receive treatment and so was not included in the analysis of safety.
Replication	This is a prospective two-stage clinical trial and no replication was planned or conducted.
Randomization	Eligible patients were randomized using the Interactive Web Response System to receive gotistobart or docetaxel treatment stratified by histology (squamous vs non-squamous [Stage 1 only]), presence of brain metastases (yes or no), ECOG PS score (0 vs. 1) and region (US and ex-US).
Blinding	The PRESERVE-003 trial is open-label. Assessment of tumor response for efficacy endpoints was conducted by Investigators.

Reporting for specific materials, systems and methods

We require information from authors about some types of materials, experimental systems and methods used in many studies. Here, indicate whether each material, system or method listed is relevant to your study. If you are not sure if a list item applies to your research, read the appropriate section before selecting a response.

Materials & experimental systems

n/a	Involved in the study
☐	☒ Antibodies
☐	☐ Eukaryotic cell lines
☐	☐ Palaeontology and archaeology
☐	☐ Animals and other organisms
☐	☒ Clinical data
☐	☐ Dual use research of concern
☐	☐ Plants

Methods

n/a	Involved in the study
☐	☐ ChIP-seq
☐	☐ Flow cytometry
☐	☐ MRI-based neuroimaging

Antibodies

Antibodies used	Gotistobart (ONC-392/BNT316). an investigational humanized anti-CTLA-4 IgG1 monoclonal antibody
Validation	Supplied by OncoC4 Inc. who is the study sponsor.

Eukaryotic cell lines

Policy information about cell lines and Sex and Gender in Research

Cell line source(s)	*State the source of each cell line used and the sex of all primary cell lines and cells derived from human participants or vertebrate models.*
Authentication	*Describe the authentication procedures for each cell line used OR declare that none of the cell lines used were authenticated.*
Mycoplasma contamination	*Confirm that all cell lines tested negative for mycoplasma contamination OR describe the results of the testing for mycoplasma contamination OR declare that the cell lines were not tested for mycoplasma contamination.*
Commonly misidentified lines (See ICLAC register)	*Name any commonly misidentified cell lines used in the study and provide a rationale for their use.*

Palaeontology and Archaeology

Specimen provenance	*Provide provenance information for specimens and describe permits that were obtained for the work (including the name of the issuing authority, the date of issue, and any identifying information). Permits should encompass collection and, where applicable, export.*
Specimen deposition	*Indicate where the specimens have been deposited to permit free access by other researchers.*
Dating methods	*If new dates are provided, describe how they were obtained (e.g. collection, storage, sample pretreatment and measurement), where they were obtained (i.e. lab name), the calibration program and the protocol for quality assurance OR state that no new dates are provided.*

☐ Tick this box to confirm that the raw and calibrated dates are available in the paper or in Supplementary Information.

Ethics oversight	*Identify the organization(s) that approved or provided guidance on the study protocol, OR state that no ethical approval or guidance was required and explain why not.*

Note that full information on the approval of the study protocol must also be provided in the manuscript.

Animals and other research organisms

Policy information about studies involving animals; ARRIVE guidelines recommended for reporting animal research, and Sex and Gender in Research

Laboratory animals	*For laboratory animals, report species, strain and age OR state that the study did not involve laboratory animals.*
Wild animals	*Provide details on animals observed in or captured in the field; report species and age where possible. Describe how animals were caught and transported and what happened to captive animals after the study (if killed, explain why and describe method; if released, say where and when) OR state that the study did not involve wild animals.*
Reporting on sex	*Indicate if findings apply to only one sex; describe whether sex was considered in study design, methods used for assigning sex. Provide data disaggregated for sex where this information has been collected in the source data as appropriate; provide overall numbers in this Reporting Summary. Please state if this information has not been collected. Report sex-based analyses where performed, justify reasons for lack of sex-based analysis.*
Field-collected samples	*For laboratory work with field-collected samples, describe all relevant parameters such as housing, maintenance, temperature, photoperiod and end-of-experiment protocol OR state that the study did not involve samples collected from the field.*
Ethics oversight	*Identify the organization(s) that approved or provided guidance on the study protocol, OR state that no ethical approval or guidance was required and explain why not.*

Note that full information on the approval of the study protocol must also be provided in the manuscript.

Clinical data

Policy information about clinical studies

All manuscripts should comply with the ICMJE guidelines for publication of clinical research and a completed CONSORT checklist must be included with all submissions.

Clinical trial registration	ClinicalTrials.gov: NCT05671510
Study protocol	Submitted with manuscript for oversight by peer review
Data collection	Patients with metastatic NSCLC were randomized into Stage 1 of the study from June 27, 2023, to September 3, 2024, in study centers in the United States, Australia, China, Korea, and the United Kingdom. Patients were enrolled from participating oncology centres (academic hospitals and community clinics).
Outcomes	The study primary endpoint was OS, and secondary endpoints included investigator-assessed PFS and ORR per RECIST 1.1, and safety in terms of the incidence of AEs (treatment-emergent), TRAEs, irAEs, and rates of treatment discontinuation due to AEs. .<br>OS was monitored for the duration of the study. Tumor response and PFS per RECIST 1.1 were determined by conducting computed tomography or magnetic resonance imaging at baseline (within 28 days prior to day 1 of cycle 1) and every 9 ± 1 weeks for the first year and every 16 ± 3 weeks after the first year until confirmed progressive disease. Upon observation of response or progression, a confirmatory scan was conducted within 4–6 weeks of the initial evaluation. Patients discontinuing treatment for any reason other than disease progression were scanned every 9 ± 1 weeks until disease progression or start of another anticancer treatment for determination of PFS.<br>AEs were reported and graded according to NCI CTCAE v5.0 by the study investigator, who also assessed potential relatedness to study treatment. Serious AEs and irAEs were collected from the start of study treatment to 90 days after the last dose of the treatment or to the date that the patient initiated new anticancer therapy, whichever came first. Gotistobart dosing was delayed for any AE considered to be immune-related, and restarted at investigator's discretion once toxicity had resolved, with dose modifications as per protocol-specified management recommendations for irAEs. Other safety evaluations (vital signs, physical examination, ECOG PS, medical history, and laboratory tests) were performed at baseline and at regular intervals throughout the study or whenever medically indicated.<br>OS was defined as time from randomization to death due to any cause. Patients without reported death were censored at the last date known to be alive. OS curves were generated using Kaplan-Meier estimates. Hazard ratios and associated 95% CIs were calculated from a Cox proportional-hazards model. ORR was determined per RECIST 1.1 and the corresponding 95% confidence intervals were calculated using the exact binomial distribution. PFS was defined as the time from the date of randomization till the date of the first documentation of progressive disease or death by any cause (whichever occurred first); for patients who had no disease progression and were alive at the time of analysis, the date of the last tumor assessment was taken as the censored date; for patients who started new anticancer therapy prior to disease progression or death, the date of the last tumor assessment on or before initiation of new anticancer therapy was taken as the censored date; for patients with disease progression or who died after more than one missed visit, the date of the last tumor assessment before the missed visit was taken as the censored date. PFS was analyzed using the same method as OS.<br>Safety analyses were conducted in all patients with squamous NSCLC who received at least 1 dose of study medication and were summarized using descriptive statistics.

Dual use research of concern

Policy information about dual use research of concern

Hazards

Could the accidental, deliberate or reckless misuse of agents or technologies generated in the work, or the application of information presented in the manuscript, pose a threat to:

No	Yes	
☐	☐	Public health
☐	☐	National security
☐	☐	Crops and/or livestock
☐	☐	Ecosystems
☐	☐	Any other significant area

Experiments of concern

Does the work involve any of these experiments of concern:

No	Yes	
☐	☐	Demonstrate how to render a vaccine ineffective
☐	☐	Confer resistance to therapeutically useful antibiotics or antiviral agents
☐	☐	Enhance the virulence of a pathogen or render a nonpathogen virulent
☐	☐	Increase transmissibility of a pathogen
☐	☐	Alter the host range of a pathogen
☐	☐	Enable evasion of diagnostic/detection modalities
☐	☐	Enable the weaponization of a biological agent or toxin
☐	☐	Any other potentially harmful combination of experiments and agents

Plants

Seed stocks

Report on the source of all seed stocks or other plant material used. If applicable, state the seed stock centre and catalogue number. If plant specimens were collected from the field, describe the collection location, date and sampling procedures.

Novel plant genotypes

Describe the methods by which all novel plant genotypes were produced. This includes those generated by transgenic approaches, gene editing, chemical/radiation-based mutagenesis and hybridization. For transgenic lines, describe the transformation method, the number of independent lines analyzed and the generation upon which experiments were performed. For gene-edited lines, describe the editor used, the endogenous sequence targeted for editing, the targeting guide RNA sequence (if applicable) and how the editor was applied.

Authentication

Describe any authentication procedures for each seed stock used or novel genotype generated. Describe any experiments used to assess the effect of a mutation and, where applicable, how potential secondary effects (e.g. second site T-DNA insertions, mosiacism, off-target gene editing) were examined.

ChIP-seq

Data deposition

☐ Confirm that both raw and final processed data have been deposited in a public database such as GEO.

☐ Confirm that you have deposited or provided access to graph files (e.g. BED files) for the called peaks.

Data access links
May remain private before publication.

For "Initial submission" or "Revised version" documents, provide reviewer access links. For your "Final submission" document, provide a link to the deposited data.

Files in database submission

Provide a list of all files available in the database submission.

Genome browser session
(e.g. UCSC)

Provide a link to an anonymized genome browser session for "Initial submission" and "Revised version" documents only, to enable peer review. Write "no longer applicable" for "Final submission" documents.

Methodology

Replicates	*Describe the experimental replicates, specifying number, type and replicate agreement.*
Sequencing depth	*Describe the sequencing depth for each experiment, providing the total number of reads, uniquely mapped reads, length of reads and whether they were paired- or single-end.*
Antibodies	*Describe the antibodies used for the ChIP-seq experiments; as applicable, provide supplier name, catalog number, clone name, and lot number.*
Peak calling parameters	*Specify the command line program and parameters used for read mapping and peak calling, including the ChIP, control and index files used.*
Data quality	*Describe the methods used to ensure data quality in full detail, including how many peaks are at FDR 5% and above 5-fold enrichment.*
Software	*Describe the software used to collect and analyze the ChIP-seq data. For custom code that has been deposited into a community repository, provide accession details.*

Flow Cytometry

Plots

Confirm that:

- [ ] The axis labels state the marker and fluorochrome used (e.g. CD4-FITC).
- [ ] The axis scales are clearly visible. Include numbers along axes only for bottom left plot of group (a 'group' is an analysis of identical markers).
- [ ] All plots are contour plots with outliers or pseudocolor plots.
- [ ] A numerical value for number of cells or percentage (with statistics) is provided.

Methodology

Sample preparation	*Describe the sample preparation, detailing the biological source of the cells and any tissue processing steps used.*
Instrument	*Identify the instrument used for data collection, specifying make and model number.*
Software	*Describe the software used to collect and analyze the flow cytometry data. For custom code that has been deposited into a community repository, provide accession details.*
Cell population abundance	*Describe the abundance of the relevant cell populations within post-sort fractions, providing details on the purity of the samples and how it was determined.*
Gating strategy	*Describe the gating strategy used for all relevant experiments, specifying the preliminary FSC/SSC gates of the starting cell population, indicating where boundaries between "positive" and "negative" staining cell populations are defined.*

- [ ] Tick this box to confirm that a figure exemplifying the gating strategy is provided in the Supplementary Information.

Magnetic resonance imaging

Experimental design

Design type	*Indicate task or resting state; event-related or block design.*
Design specifications	*Specify the number of blocks, trials or experimental units per session and/or subject, and specify the length of each trial or block (if trials are blocked) and interval between trials.*
Behavioral performance measures	*State number and/or type of variables recorded (e.g. correct button press, response time) and what statistics were used to establish that the subjects were performing the task as expected (e.g. mean, range, and/or standard deviation across subjects).*

Acquisition

Imaging type(s) | *Specify: functional, structural, diffusion, perfusion.*

Field strength | *Specify in Tesla*

Sequence & imaging parameters | *Specify the pulse sequence type (gradient echo, spin echo, etc.), imaging type (EPI, spiral, etc.), field of view, matrix size, slice thickness, orientation and TE/TR/flip angle.*

Area of acquisition | *State whether a whole brain scan was used OR define the area of acquisition, describing how the region was determined.*

Diffusion MRI ☐ Used ☐ Not used

Preprocessing

Preprocessing software | *Provide detail on software version and revision number and on specific parameters (model/functions, brain extraction, segmentation, smoothing kernel size, etc.).*

Normalization | *If data were normalized/standardized, describe the approach(es): specify linear or non-linear and define image types used for transformation OR indicate that data were not normalized and explain rationale for lack of normalization.*

Normalization template | *Describe the template used for normalization/transformation, specifying subject space or group standardized space (e.g. original Talairach, MNI305, ICBM152) OR indicate that the data were not normalized.*

Noise and artifact removal | *Describe your procedure(s) for artifact and structured noise removal, specifying motion parameters, tissue signals and physiological signals (heart rate, respiration).*

Volume censoring | *Define your software and/or method and criteria for volume censoring, and state the extent of such censoring.*

Statistical modeling & inference

Model type and settings | *Specify type (mass univariate, multivariate, RSA, predictive, etc.) and describe essential details of the model at the first and second levels (e.g. fixed, random or mixed effects; drift or auto-correlation).*

Effect(s) tested | *Define precise effect in terms of the task or stimulus conditions instead of psychological concepts and indicate whether ANOVA or factorial designs were used.*

Specify type of analysis: ☐ Whole brain ☐ ROI-based ☐ Both

Statistic type for inference | *Specify voxel-wise or cluster-wise and report all relevant parameters for cluster-wise methods.*

(See Eklund et al. 2016)

Correction | *Describe the type of correction and how it is obtained for multiple comparisons (e.g. FWE, FDR, permutation or Monte Carlo).*

Models & analysis

n/a	Involved in the study
☐	☐ Functional and/or effective connectivity
☐	☐ Graph analysis
☐	☐ Multivariate modeling or predictive analysis

Functional and/or effective connectivity | *Report the measures of dependence used and the model details (e.g. Pearson correlation, partial correlation, mutual information).*

Graph analysis | *Report the dependent variable and connectivity measure, specifying weighted graph or binarized graph, subject- or group-level, and the global and/or node summaries used (e.g. clustering coefficient, efficiency, etc.).*

Multivariate modeling and predictive analysis | *Specify independent variables, features extraction and dimension reduction, model, training and evaluation metrics.*
